# Ligand Redox Activity of Organonickel Radical Complexes Governed by the Geometry

**DOI:** 10.1021/jacs.3c07031

**Published:** 2023-09-11

**Authors:** Gregory A. Dawson, Qiao Lin, Michelle C. Neary, Tianning Diao

**Affiliations:** † Department of Chemistry, New York University, 100 Washington Square East, New York, New York 10003, United States; ‡ Department of Chemistry, CUNY − Hunter College, 695 Park Avenue, New York, New York 10065, United States

## Abstract

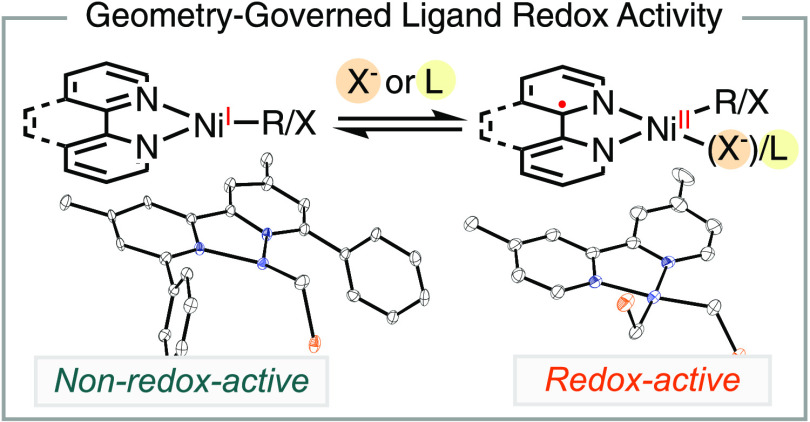

Nickel-catalyzed cross-coupling reactions often employ bidentate π-acceptor *N*-ligands to facilitate radical pathways. This report presents the synthesis and characterization of a series of organonickel radical complexes supported by bidentate *N*-ligands, including bpy, phen, and pyrox, which are commonly proposed and observed intermediates in catalytic reactions. Through a comparison of relevant analogues, we have established an empirical rule governing the electronic structures of these nickel radical complexes. The *N*-ligands exhibit redox activity in four-coordinate, square-planar nickel radical complexes, leading to the observation of ligand-centered radicals. In contrast, these ligands do not display redox activity when supporting three-coordinate, trigonal planar nickel radical complexes, which are better described as nickel-centered radicals. This trend holds true irrespective of the nature of the actor ligands. These results provide insights into the beneficial effect of coordinating salt additives and solvents in stabilizing nickel radical intermediates during catalytic reactions by modulating the redox activity of the ligands. Understanding the electronic structures of these active intermediates can contribute to the development and optimization of nickel catalysts for cross-coupling reactions.

## Introduction

**Scheme 1:**
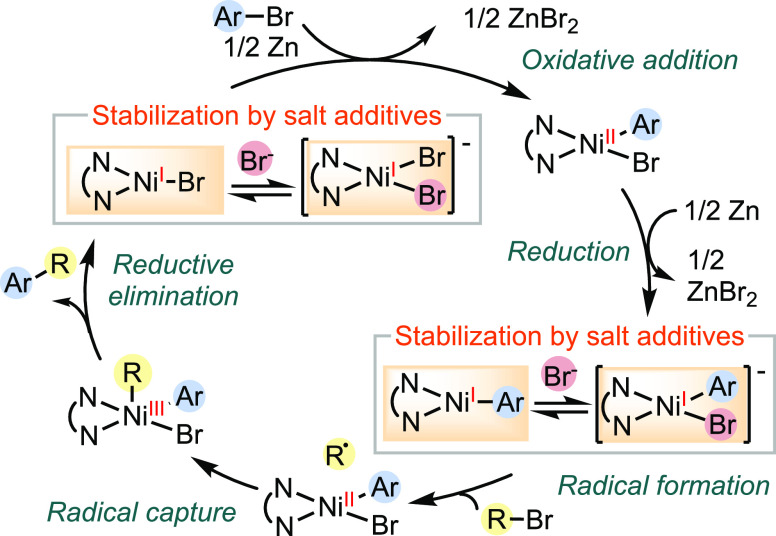
Mechanism of Nickel-Catalyzed Cross-Electrophile Coupling Reactions, Key Nickel(I) Radical Intermediates, and Their Stabilization by Halide Coordination

Recent advancements in the field of cross-coupling reactions have harnessed the reactivity of nickel catalysts to initiate and propagate radical reactions^1−6^ facilitated by nickel radical intermediates.^7−9^ Radical pathways involving nickel(I) and (III) intermediates for generating and regulating carbon radicals have paved the way for the application of nickel catalysts in stereo-convergent coupling,^10^ photoredox, and electrocatalytic reactions,^11^ significantly broadening the scope and application of cross-coupling reactions. Notably, the cross-electrophile coupling reaction exemplifies the crucial role of distinct nickel(I)-halide^12^ and nickel(I)-aryl^13^ species in activating C(sp^2^) and C(sp^3^) electrophiles, respectively, through diverse mechanisms (Scheme 1).^14,15^ Recent research has also revealed the formation of nickel(III) intermediates through the rapid capture of carbon radicals by nickel(II) complexes, with a barrier of 7–9 kcal/mol, enabling efficient reductive elimination.^16^

The development of transition metal-catalyzed reactions relies on the careful selection of appropriate ligands to enhance reactivity and stabilize intermediates. In many cross-coupling reactions, strong σ-donor and π-acceptor ligands, such as terpyridine (terpy),^17^ bipyridine (bpy),^18−20^ 1,10-phenanthroline (phen),^18^ pyridine-bis(oxazoline) (pybox),^21^ α-diimine,^22^ and pyridine-oxazoline (pyrox),^23^ have proven instrumental. These ligands can be redox-active, allowing for the stabilization of low-valent nickel radical species by delocalizing the unpaired electron into the π* orbital of the ligand.^24^ This redox activity plays a pivotal role in promoting radical pathways and differentiates the reactivity of nickel catalysts from the traditional two-electron pathways mediated by palladium catalysts. The lack of redox activity in ligands can lead to significant differences in the redox potentials of the nickel intermediates, resulting in notable changes in the reaction mechanism.^25^ Therefore, it is crucial to characterize the redox activity of ligands with respect to various nickel intermediates for comprehensive understanding of the reaction mechanism and informing catalyst optimization.

Moreover, the presence of anionic ligands can influence the speciation, complexation, and electronic structure of catalytic nickel intermediates, adding complexity to the catalyst effect and the mechanistic profile (Scheme 1). Additives such as MgCl_2_ and KI have proven crucial in promoting catalytic reactions, such as cross-electrophile coupling reactions.^14^ While recent studies have started to elucidate the beneficial effects of additives on these reactions,^26^ the influence of anionic ligands on the electronic structure of nickel radical species and, consequently, the stability and reactivity of nickel intermediates remains unexplored.

**Scheme 2:**
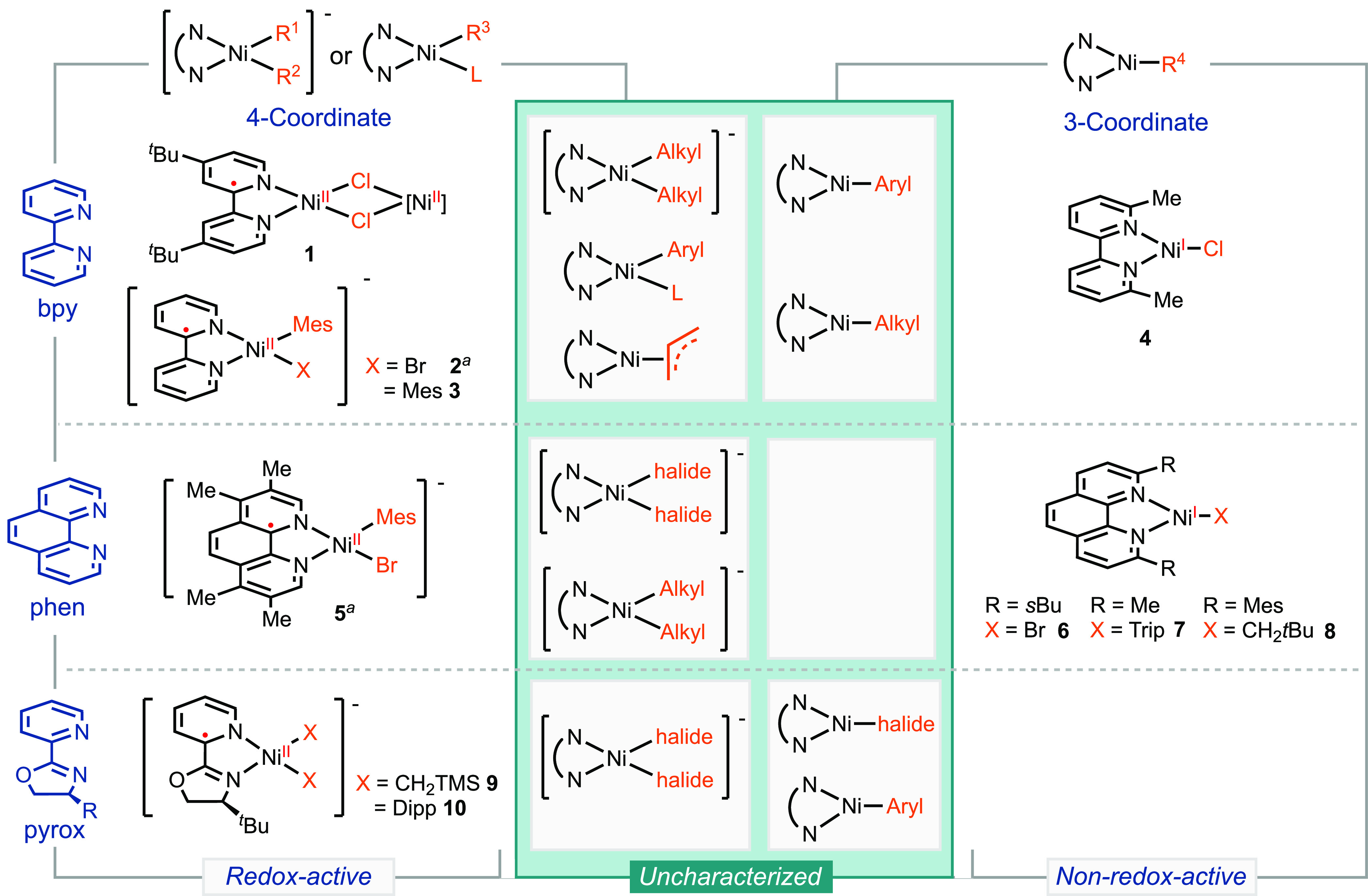
Electronic Structures of Precedent and Uncharacterized Organonickel Radical Complexes Structure characterization by X-ray crystallography is not available.

Surveying organometallic studies on nickel radical complexes ligated with bidentate *N*-ligands reveals that the redox activity of a ligand can vary among different complexes (Scheme 2, Table S1). The well-defined four-coordinate [(dtbpy)Ni(μ-Cl)]_2_**1** (dtbpy = 4,4′-di-*tert*-butyl-2,2′-bipyridine) adopts a distorted square-planar geometry with a dihedral angle of 26.3° (0° for square planar and 90° for tetrahedral) and has been characterized as a Ni(II) complex ligated with [dtbpy]^•–^.^18^ Similarly, two other four-coordinate nickel radical complexes, [(bpy)Ni(Mes)(Br)]^−^ (Mes = 2,4,6-mesityl) **2**(19) and [(bpy)Ni(Mes)_2_]^−^**3**,^20^ feature redox-active ligands. In contrast, the three-coordinate (^Me^bpy)NiCl **4** (^Me^bpy = 6,6′-dimethyl-2,2′-bpy) exhibits a nickel-centered radical, with ^Me^bpy being non-redox-active.^27^ In other examples, the formation of oligomers can alter the geometry of the complexes and impact the assignment of the electronic structure.^13,28^

Phen-ligated nickel complexes also display variations in redox activity. [(Tmphen)Ni(Mes)Br]^−^**5** (tmphen = 3,4,7,8-tetramethyl-1,10-phenanthroline) has been characterized as redox-active based on electrochemical and spectroscopic studies,^19,29^ while three-coordinate nickel complexes, such as **6**,^14^**7**,^30^ and **8**,^18^ exhibit nickel-centered radicals. However, the investigation of pyrox complexes remains limited, with only (pyrox)Ni complexes, **9** and **10**,^27^ having been fully characterized as ligand-centered radicals. A comprehensive study on three-coordinate (pyrox)Ni-halide and -aryl/alkyl complexes is still lacking.

A common perception is that the redox activity of a ligand is influenced by the nature of the actor ligands. Strong-field ligands, such as alkyl and aryl groups, can promote redox activity of the auxiliary ligands, whereas weak-field ligands, such as halides, are expected to result in nonredox activity of the auxiliary ligands.^31,32^ Data summarized in Scheme 2, however, reveal a potential correlation between the redox activity of a ligand and the coordination number and geometry of the complexes. Four-coordinate, square-planar complexes demonstrate ligand redox activity, whereas the same ligands in three-coordinate complexes do not display redox activity. Nevertheless, several classes of nickel radical complexes lack structural characterization, which prevents the verification and generalization of this postulate. Such uncharacterized molecules include three-coordinate (bpy)Ni-aryl and -alkyl complexes, four-coordinate (phen)Ni-dihalide and -dialkyl complexes, and three-coordinate (pyrox)Ni-halide and -aryl complexes (Scheme 2).

To address this knowledge gap, this report presents a synthesis and spectroscopic study focused on the electronic structures of nickel radical complexes. By completing the missing pieces and expanding the series of organonickel radical complexes, we provide compelling evidence that establishes the correlation between the redox activity of the ligand and the geometry and coordination number of nickel radical complexes bearing bidentate *N-*ligands. This finding is significant as the coordination number and geometry of nickel complexes can be modulated by the selection of reaction conditions and additives. Insights into the electronic structures of the active intermediates will inform the development and optimization of catalysts.

## Results

### Bpy-Ligated Nickel Radical Complexes

**Scheme 3:**
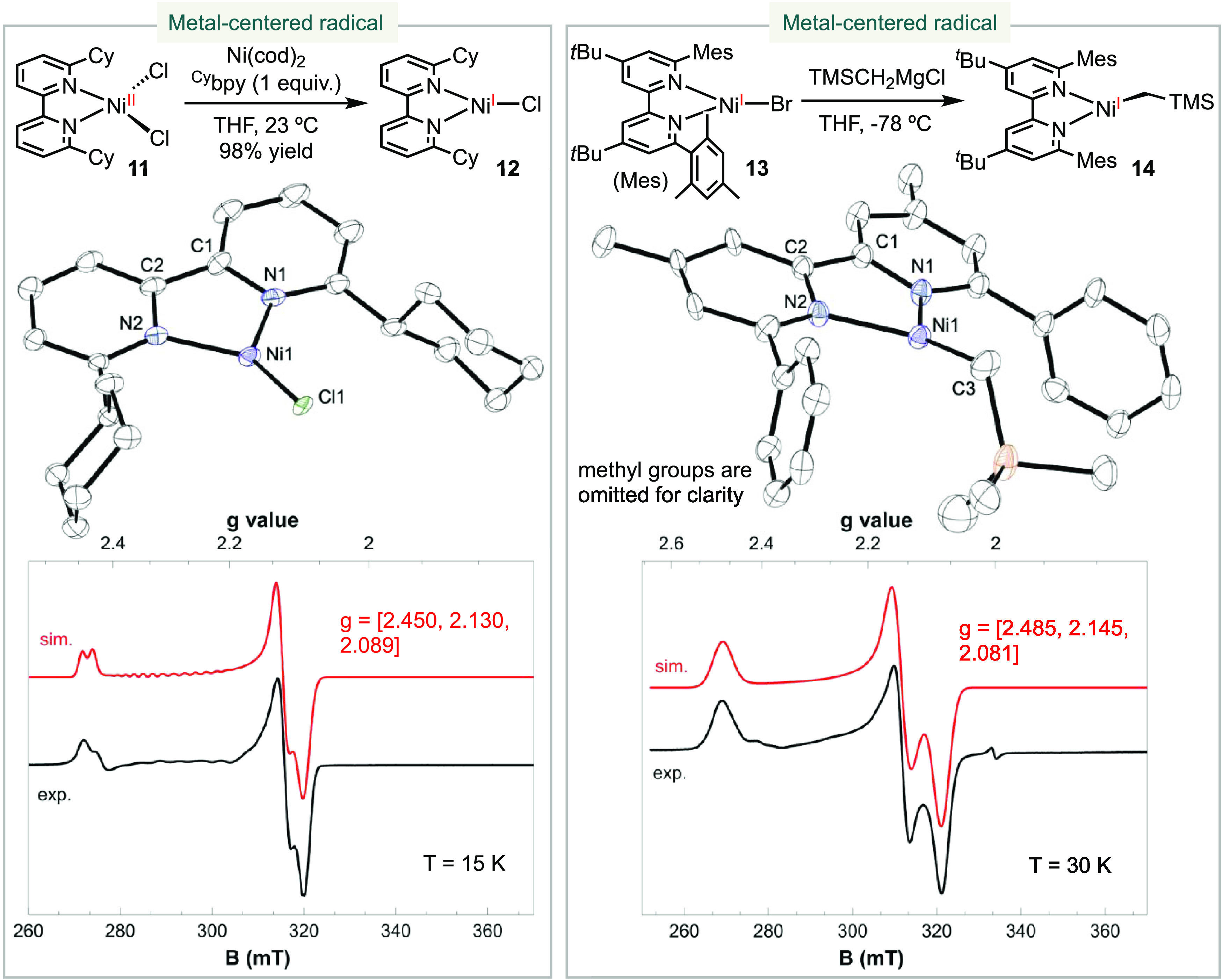
Synthesis and Characterization of Three-Coordinate (bpy)Ni(I)-Halide and -Alkyl Complexes

Our investigation focused on characterizing the electronic structures of three-coordinate (bpy)Ni(I)-halide, -alkyl, and -aryl complexes. We first synthesized 6,6′-dicyclohexyl-2,2′-bpy (^Cy^bpy) and the corresponding (^Cy^bpy)NiCl_2_**11**, with the hypothesis that the steric bulkiness of ^Cy^bpy could stabilize a three-coordinate Ni(I) halide (Scheme 3). Comproportionation of (^Cy^bpy)NiCl_2_**11** with Ni(cod)_2_ in the presence of ^Cy^bpy generated (^Cy^bpy)NiCl **12** as an emerald crystalline solid in 98% yield. Single-crystal X-ray crystallography analysis confirmed a trigonal planar geometry, similar to the previously reported analogous complex (^Me^bpy)NiCl **4**.^27^ The bond angles of N1–Ni–Cl and N2–Ni–Cl were determined to be 136.99(13) and 139.58(13)°, respectively, indicating a symmetrical structure. However, the dihedral angel of N1–Ni–N2–Cl was measured to be 170.8°, revealing a slight deviation from perfect trigonal planar geometry. The EPR spectrum of **12** at 15 K exhibited a rhombic signal with *g* values of [2.450, 2.130, 2.089], consistent with a nickel-centered radical.

Subsequently, we synthesized (^Mes^bpy)Ni(I) bromide **13** (^Mes^bpy = 4,4′-di-*tert*-butyl-6,6′-dimesityl-2,2′-bpy) as a dark yellow-green complex through the same comproportionation reaction between Ni(cod)_2_ and (^Mes^bpy)NiBr_2_. While **13** decomposed upon storage at room temperature, immediate transmetalation of TMSCH_2_MgCl with **13** led to the formation of (^Mes^bpy)Ni(CH_2_TMS) **14** as an orange solid (Scheme 3). The dihedral angles of N1–N2–Ni–C3 were 165.70 and 165.99°. Despite the disorder, both dihedral angles are comparable and suggest a slightly distorted trigonal planar geometry. This geometry resembles that of compound **7**, which exhibited a N1–N2–Ni–C3 dihedral angle of 167.84°.^30^ The EPR spectrum of **14** at 30 K displayed a rhombic signal with *g* values of [2.485, 2.145, 2.081], indicating a nickel-centered radical.

**Scheme 4:**
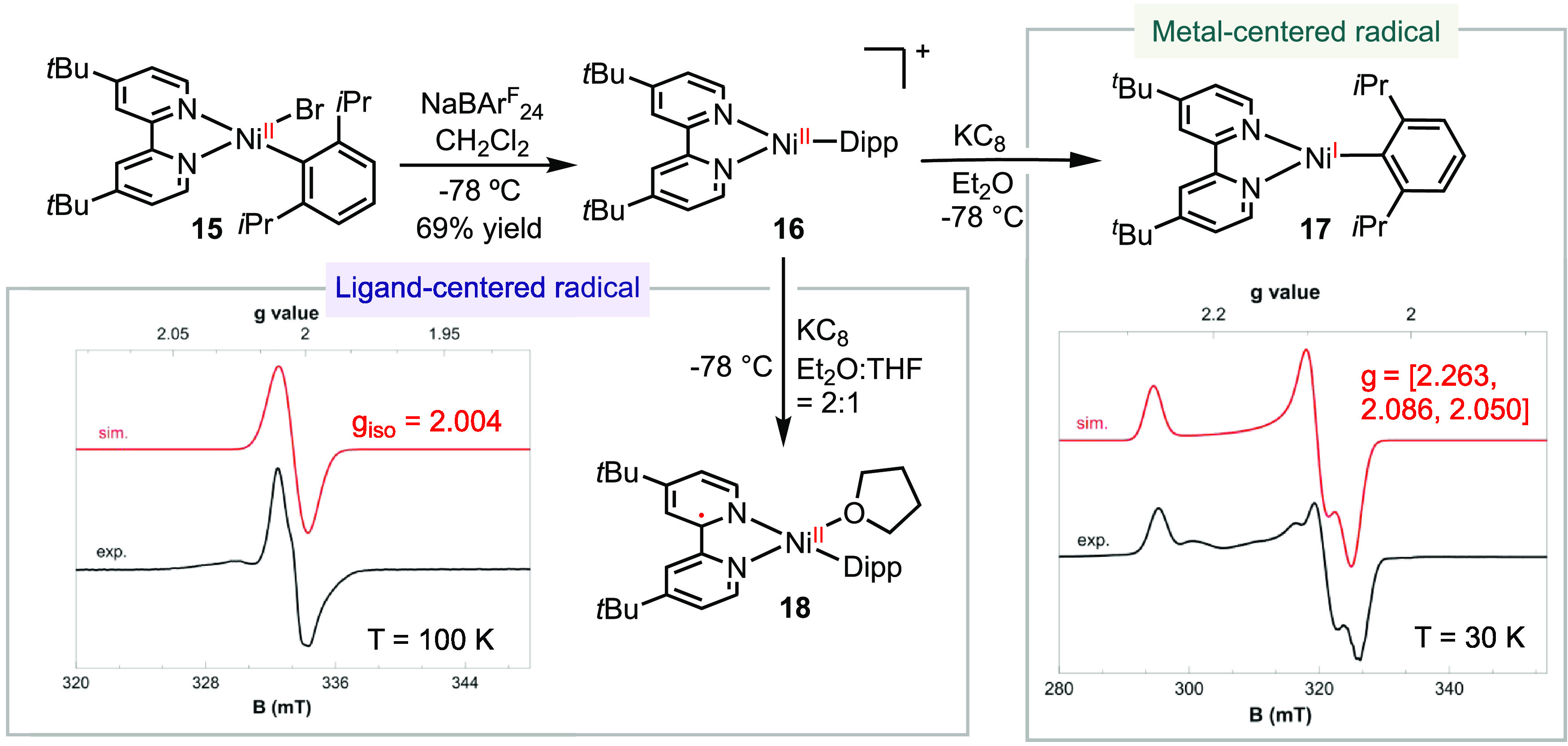
Electronic Structures of [(bpy)Ni(I)-Aryl] Radical Complexes and the Effect of Solvent Coordination

We then investigated the electronic structure of radical (bpy)Ni-aryl derivatives. Previous electrochemical and spectrochemical studies suggested the formation of four-coordinate, halide-bound species from the one-electron reduction of Ni(II) aryl halide complexes, but structural characterization was insufficient.^19^ Our attempts to isolate such an intermediate by chemically reducing (bpy)Ni(Mes)Br or (dtbpy)Ni(Mes)Br resulted in decomposition, leading to the formation of bis-mesityl Ni(II) complexes. Therefore, we adopted a different approach by abstracting the halide from nickel(II) aryl halide complexes prior to reduction. By oxidative addition of DippBr (Dipp = 2,6-diisopropylphenyl) to Ni(cod)_2_ in the presence of dtbpy, we successfully generated (dtbpy)Ni(Dipp)(Br) **15** as a dark red complex with a yield of 66% (Scheme 4). Alternatively, **15** could be synthesized through transmetalation of DippMgBr with (dtbpy)Ni(acac)_2_, resulting in a similar yield. Treatment of **15** with NaBAr_24_^F^ (BAr_24_^F^ = tetrakis(3,5-bis(trifluoromethyl)phenyl)borate) in dichloromethane resulted in bromide abstraction and the formation of the cationic [(dtbpy)Ni(II)Dipp]^+^[BAr_24_^F^]^−^ complex **16**. This assignment was supported by observed shifts in the ^1^H NMR resonances (Figure S29) and HRMS data, confirming the absence of bromide in the resulting molecule.

The reduction of **16** with KC_8_ at −78 °C in Et_2_O generated an olive-green species **17** (Scheme 4). The EPR spectrum of **17** at 30 K exhibited a rhombic signal with g values of [2.263, 2.086, 2.050]. In contrast, when the reduction by KC_8_ was conducted in the presence of THF as a cosolvent, the resulting product **18** appeared as a darker forest green solution compared to **17**. The EPR spectrum of **18** displayed an isotropic signal with a *g*_iso_ value of 2.004 at 100 K. These distinct EPR data suggest that **17** corresponds to a nickel-centered radical, while **18** is a ligand-centered radical.

**Scheme 5:**
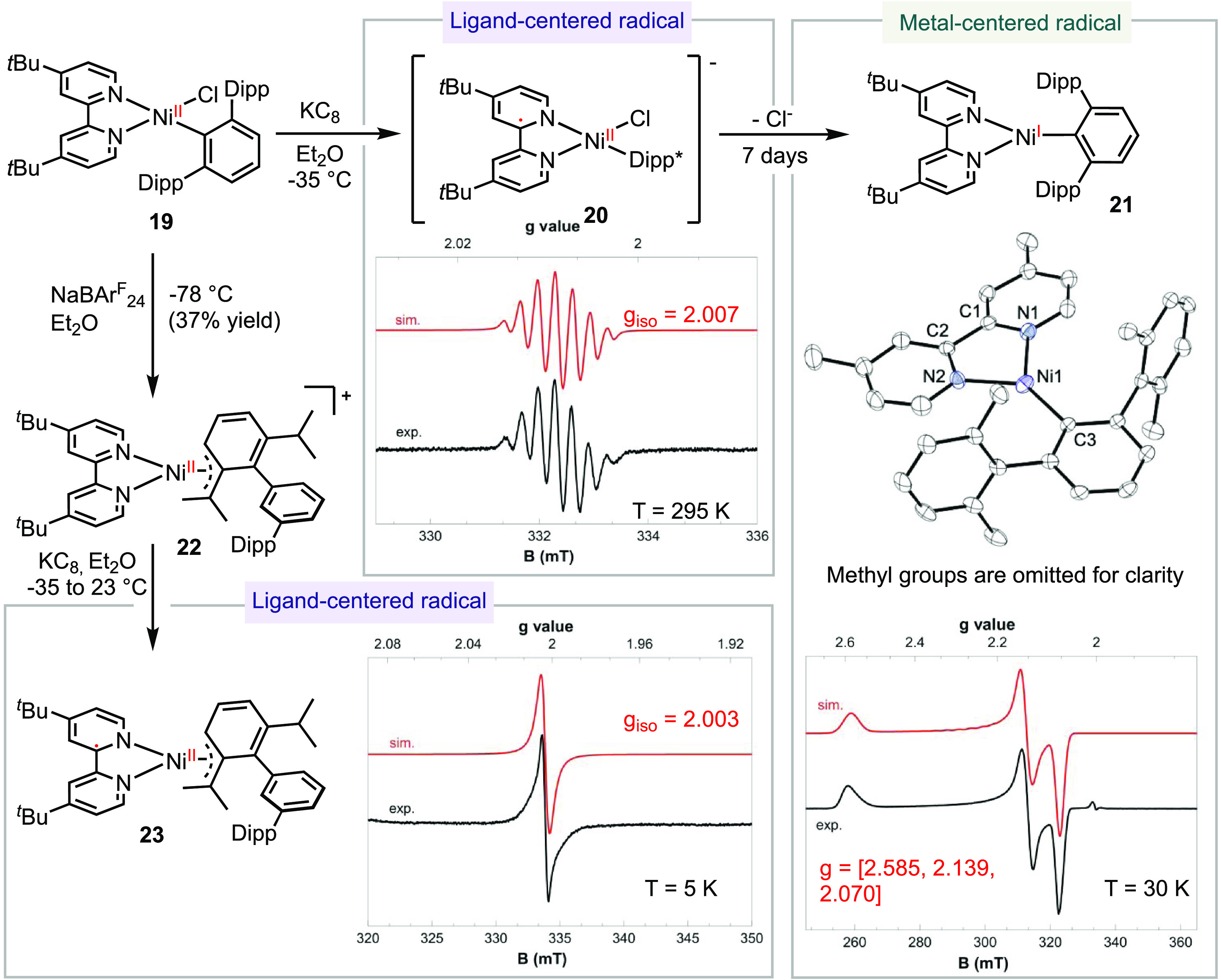
Synthesis and Characterization of Three- and Four-Coordinate (bpy)Ni(I)-Aryl and -Allyl Complexes

To further investigate the electronic structures of (dtbpy)Ni-aryl radical complexes, we employed a bulkier aryl ligand, 2,6-bis-Dipp-phenyl (Dipp*), to stabilize the complexes without introducing substituents on dtbpy (Scheme 5). We successfully synthesized (dtbpy)Ni(Dipp*)Cl **19** by adding (Dipp*)Li to [(dtbpy)Ni(μ-Cl)]_2_.^12,33^ Reduction of **19** with KC_8_ yielded intermediate **20**, as a forest green solution. The EPR spectrum of **20** exhibited an isotropic signal at 295 K with a *g*_iso_ value of 2.007, indicating a ligand-centered radical. The observed hyperfine splitting was attributed to the coupling of the radical with two *N*- and two *H-*atoms on the dtbpy ligand. Further crystallization of a solution of **20** in pentane led to the formation of a new species **21**. X-ray crystallography established that **21** was a tri-coordinate (dtbpy)Ni(Dipp*) complex with a trigonal planar geometry (Scheme 5). The dihedral angles of N1–N2–Ni–C3 in the two crystallographically unique molecules were measured to be 171.12° and 158.37°. The EPR spectrum of **21** at 30 K exhibited a rhombic signal with *g* values of [2.585, 2.139, 2.070]. Based on the structure of **21** and a comparison of the EPR spectra of **20** and **21**, we assigned the structure of **20** as the (dtbpy)Ni(Dipp*)Cl radical anion, featuring a ligand-centered radical coordinated to a Ni(II) center.

Our attempt to abstract chloride from **19** using 1 equiv of NaBAr_24_^F^ resulted in the formation of a π-allyl Ni(II) cationic species **22**. The structure of **22** was determined through single-crystal X-ray crystallography, revealing that Dipp* underwent a rearrangement to form η^3^-coordination, possibly via a 1,5-H shift (cf. Figure S69). Reduction of **22** with KC_8_ generated complex **23**. The isotropic EPR signal of **23**, with a *g*_iso_ value of 2.003, led us to assign its electronic structure as a π-allyl Ni(II) complex coordinated by [dtbpy]^•–^.

**Scheme 6:**
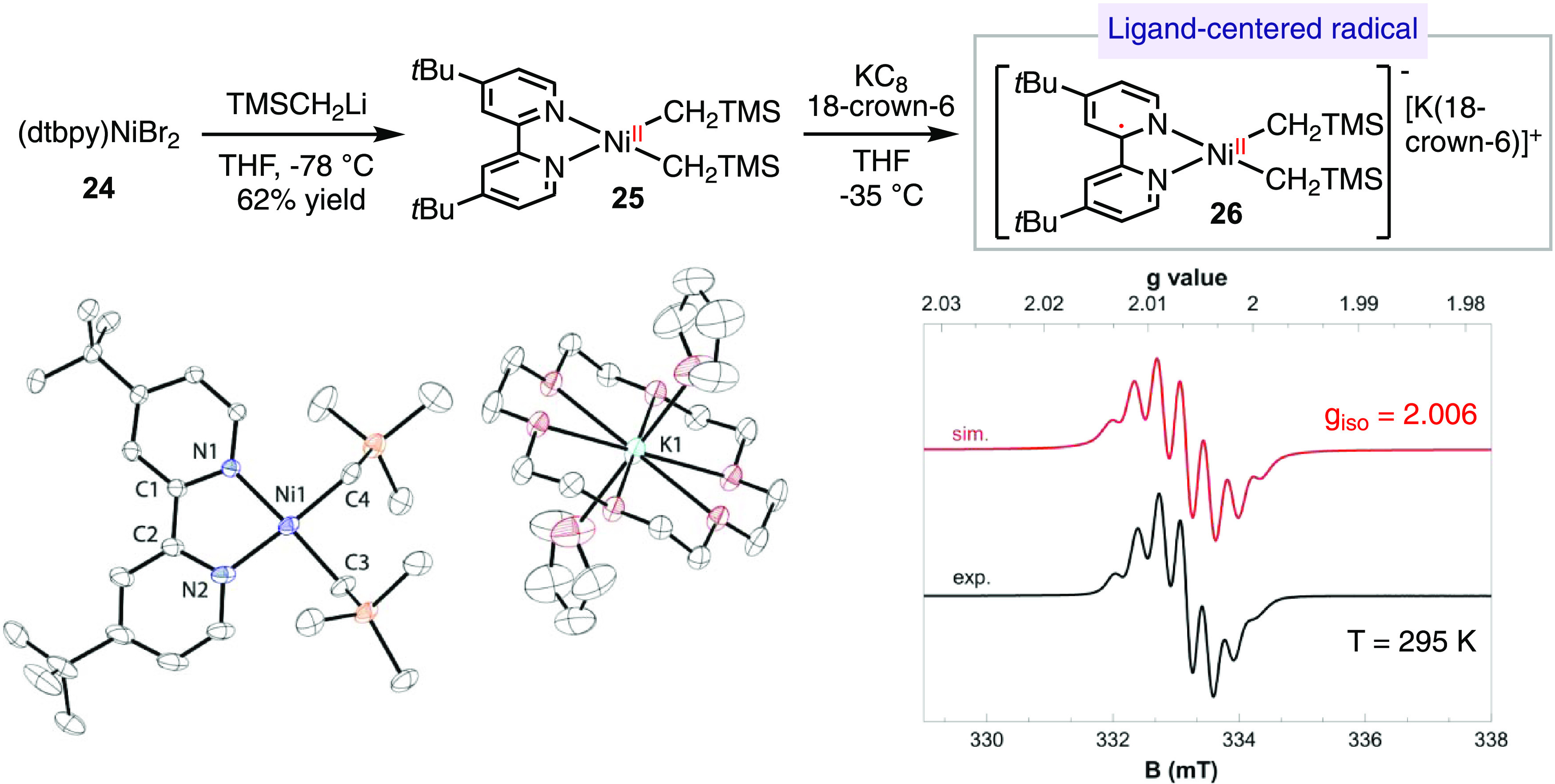
Electronic Structure of the Four-Coordinate (dtbpy)Ni-Dialkyl Radical Anion Complex

We also investigated the electronic structure of (bpy)Ni-dialkyl radical complexes by conducting the reduction of their Ni(II) analogues (Scheme 6). By adding 2 equiv of TMSCH_2_Li to (dtbpy)NiBr_2_**24** in a mixture of THF and pentane, we obtained (dtbpy)Ni(CH_2_TMS)_2_**25** in a yield of 62% as a dark green solid. Further reduction of **25** with KC_8_ in THF in the presence of 18-crown-6 furnished [(dtbpy)Ni(CH_2_TMS)_2_]^-^[18-crown-6(K)]^+^**26** as a dark red crystalline solid. The single-crystal X-ray structure of **26** displayed a square-planar geometry, reminiscent of a previously reported analogous complex, [(^*t*Bu^pyrox)Ni(CH_2_TMS)_2_]^-^[18-crown-6(K)]^+^**9**.^23^ The EPR spectrum of complex **26** exhibited an isotropic signal with a *g*_iso_ value of 2.006 and showed hyperfine splitting, which was attributed to the interaction of the radical with two *N-* and two *H-*atoms. Therefore, we assigned the electronic structure of complex **26** as a ligand-centered radical coordinated to a Ni(II) center.

The collective analysis of EPR data and single-crystal X-ray diffraction crystallography allowed for the unambiguous assignment of the electronic structures of a series of (bpy)Ni radical complexes (Table 1). Complexes **12**, **14**, **17**, and **21**, characterized as three-coordinate trigonal planar complexes, exhibited rhombic EPR signals at low-temperatures, with *g* values reflecting nickel-centered radicals. The bond lengths of the C_py_–N_py_ and the C_py_–C_py_ bonds in these complexes were similar to those observed in the (bpy)Ni(II) complex **19**, indicating the lack of redox activity of the bpy ligands in **12**, **14**, **17**, and **21**. In contrast, complexes **18**, **20**, **23**, and **26**, identified as four-coordinate square-planar complexes, displayed EPR spectra indicative of organic radicals at higher temperatures, suggesting the presence of ligand-centered radicals. Notably, the C_py_–N_py_ bond lengths in **26** were elongated compared to the nickel(I) complexes, while the C_py_–C_py_ bond length was considerably shorter. Based on these observations, we assigned the electronic structures of **18**, **20**, **23**, and **26** as nickel(II)-ligated with [bpy]^•–^.

**Table 1:** EPR and Single-Crystal X-ray Structure Parameters of (bpy)Nickel Complexes

complex	*g* values of the EPR signal	C1–N1 and C2–N2 (N_py_–C_py_) (Å)	C1–C2 (C_py_–C_py_) (Å)	location of the radical
**12**	2.450, 2.130, 2.089	1.359(6), 1.361(6)	1.466(7)	metal
**14**	2.485, 2.145, 2.081	1.364(2), 1.364(2)	1.484(3)	metal
**17**	2.263, 2.086, 2.050			metal
**18**	2.004			ligand
**19**^a^		1.364(3), 1.348(3); 1.364(3), 1.341(3)	1.472(3); 1.473(3)	
**20**	2.007			ligand
**21**^a^	2.585, 2.139, 2.070	1.354(5), 1.342(5); 1.353(5), 1.343(5)	1.470(5); 1.472(6)	metal
**23**	2.003			ligand
**26**	2.006	1.387(3), 1.383(4)	1.414(4)	ligand

a Two crystallographically independent molecules.

### Phen-Ligated Nickel Radical Complexes

**Scheme 7:**
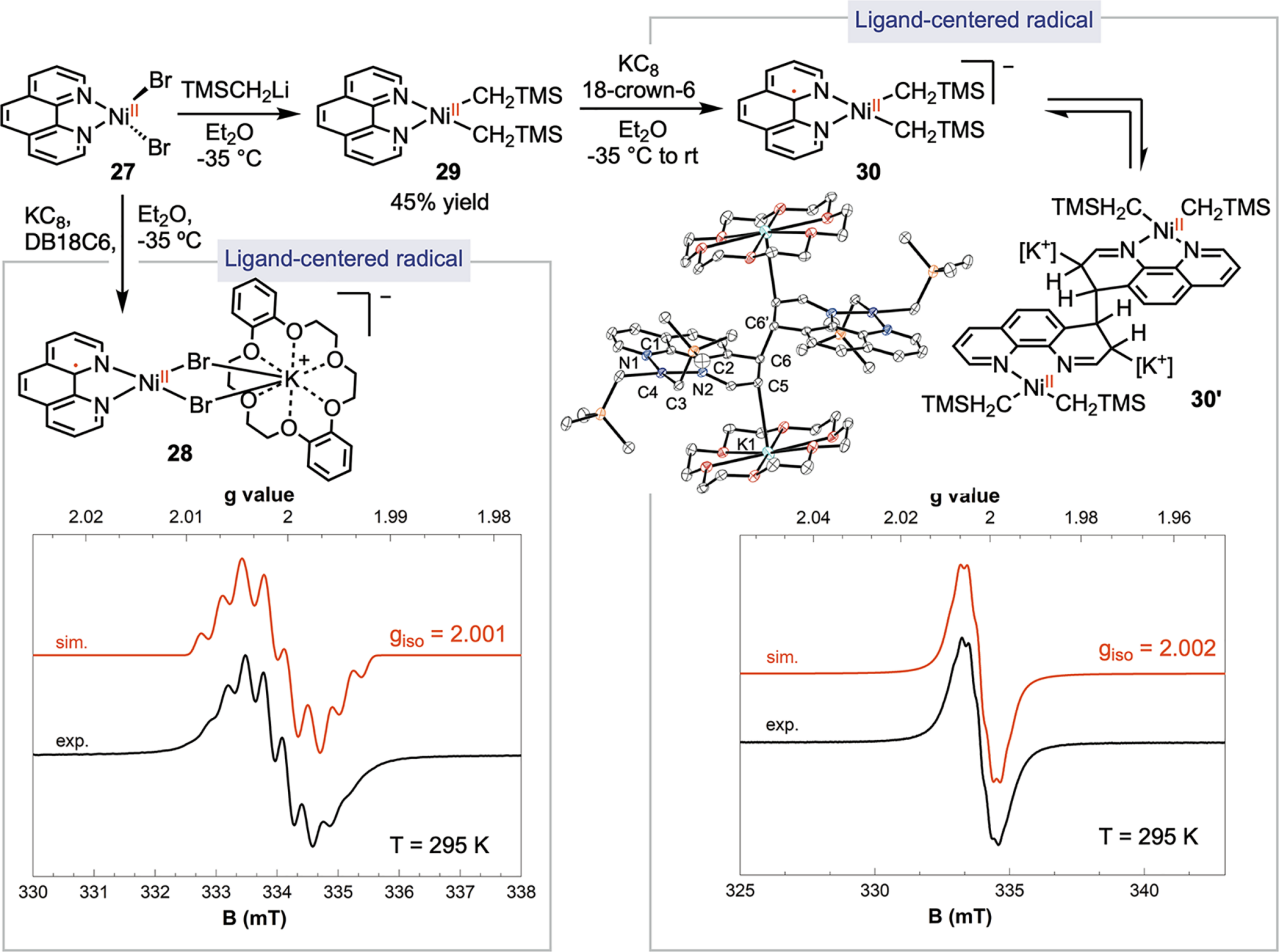
Synthesis and Electronic Structures of Four-Coordinate (phen)Nickel Radical Complexes

Phen has commonly been considered as a bpy analogue in the advancement of catalytic reactions. However, the conjugation between the two pyridine rings in phen can substantially influence its redox potentials.^34^ While a few examples of three-coordinate phen-ligated Ni(I) complexes have been documented,^12,18,30^ our investigation focused on the relatively unexplored four-coordinate phen radical complexes (Scheme 7). The reduction of (phen)NiBr_2_**27** with KC_8_ in the presence of dibenzo-18-crown-6 (DB18C6) yielded a dark violet species, **28**. The EPR spectrum displayed the presence of an organic radical with a *g*_iso_ value of 2.001 and hyperfine couplings corresponding to the interaction of the radical with two *N*- and one *H-*atoms. Despite some similarities with [phen]^•–^, the EPR signal of **28** exhibited more pronounced line-broadening, which can be attributed to the quadrupole moment of bromide, leading to faster relaxation.^35^ The EPR signal, including both intensity and hyperfine pattern, was significantly influenced by the nature of the crown ether, suggesting the association of [crown]K^+^ with **28** (cf. Figures S58 and S59). The stability of **28** closely correlated with the nature of the halide, as the complex rapidly decomposed when bromide was replaced with chloride. Unfortunately, our attempts to obtain a single crystal of **28** were unsuccessful due to its instability. Based on the analysis of the available evidence, we tentatively propose the structure of **28** as a four-coordinate complex, [K(DB18C6)]^+^[(phen)NiBr_2_]^−^, where the Ni(II) complex is ligated with a phen-centered radical.

The transmetalation of **27** with TMSCH_2_Li resulted in the formation of (phen)Ni(CH_2_TMS)_2_**29**, a navy blue solid with a yield of 47%. Reduction of **29** with 1 equiv of KC_8_ in the presence of 18-crown-6 in Et_2_O produced a dark teal solution, **30**. The EPR spectrum of **30** in THF at 295 K displayed an isotropic signal with a *g*_iso_ value of 2.002, accompanied by hyperfine coupling corresponding to the interaction of the radical with two *N-* and two *H-*nuclei. We assigned the structure of **30** as [(18-crown-6)K]^+^[(phen)Ni(CH_2_TMS)_2_]^−^. Single-crystal X-ray crystallographic analysis revealed a dimeric structure, **30**′, where a single bond was formed between the carbons at the 4-positions of two phen ligands (C6–C6′ = 1.594(7) Å). Additionally, there was a close interaction between the carbon at the 3-position (C5) of phen and [K(18-crown-6)]^+^, evidenced by a C–K distance of 3.105(4) Å. The formation of bonds between C6, C5, and K disrupted aromaticity, resulting in a puckered geometry of the pyridine ring involved in the bond formation, reflecting sp^3^ hybridization of C5 and C6.

The presence of a spin = 1/2 nickel species and the X-ray structure of the dimer indicated an equilibrium between **30** and **30**′. The facile formation and cleavage of the C–C bond between the two phen ligands at the 4-position align with the electronic structure of [phen]^•–^ Ni(II) and highlight the significant population of the radical at the 4-position of phen.

### Pyrox-Ligated Nickel Radical Complexes

**Scheme 8:**
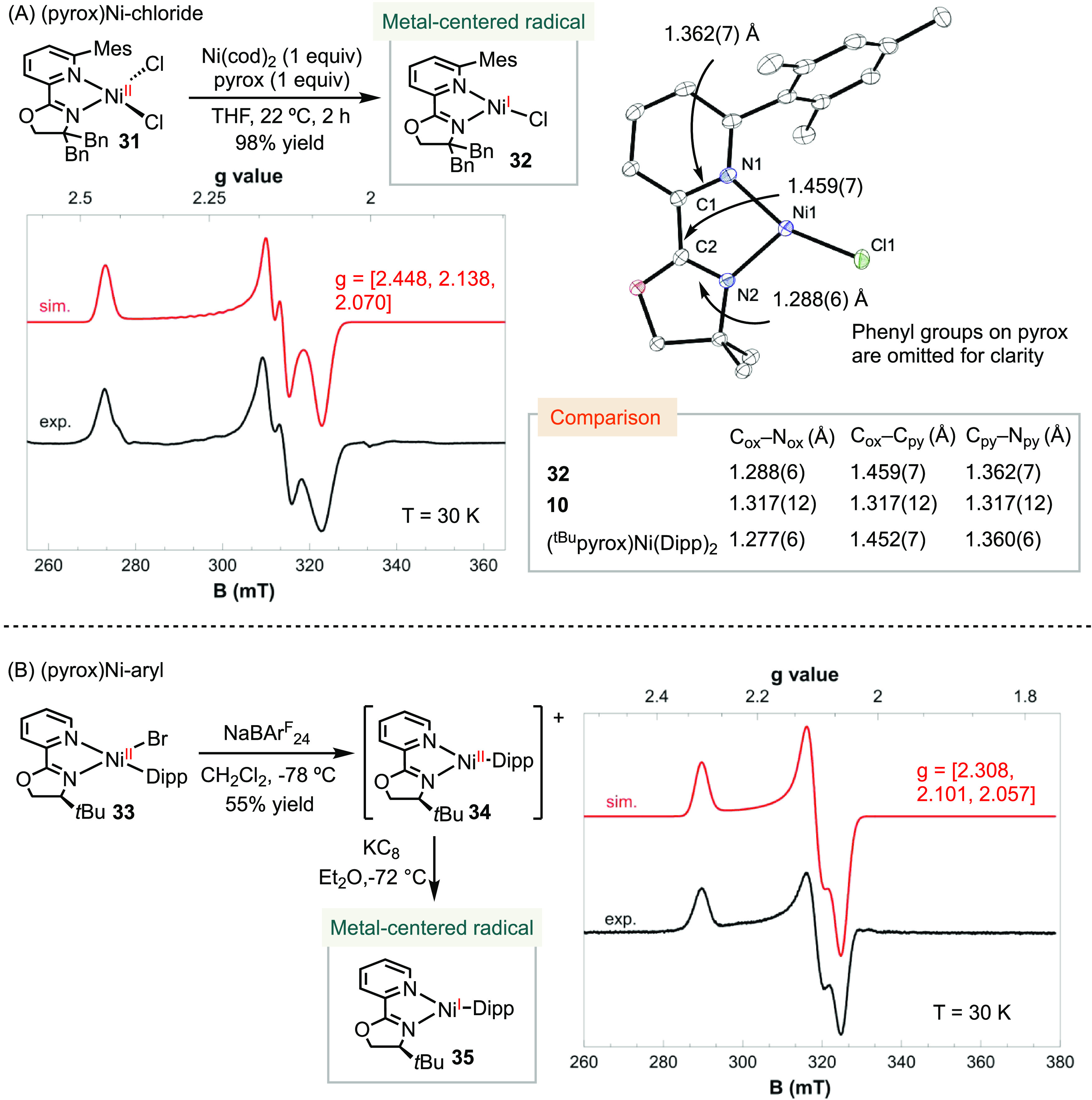
Synthesis and Characterization of Three-Coordinate (pyrox)Ni Radical Complexes

We conducted further investigations on tri-coordinate (pyrox)Ni radical complexes, which have received limited attention in previous studies.^23^ Our previous attempts in their synthesis encountered challenges due to the relatively facile dissociation of pyrox compared to bpy or phen, resulting in the formation of undesired (pyrox)_2_Ni complexes. To stabilize the molecules, we introduced di-benzyl substituents on the oxazoline and a mesityl group on the pyridine to increase the steric bulk of the pyrox ligand (Scheme 8A). By comproprotionation of (pyrox)NiCl_2_**31** and Ni(cod)_2_, we successfully synthesized (pyrox)NiCl **32** in 98% yield as a forest green crystalline solid. The X-ray crystal structure of **32** revealed an intriguing T-shape geometry with bond angles of N1–Ni–Cl of 118.07(13)° and N2–Ni–Cl of 158.38(14)°, which can be attributed to the unsymmetrical *trans-*influence of the pyridine and oxazoline motifs. The C_ox_–N_ox_, C_ox_–C_py_, and C_py_–N_py_ bond lengths were determined as 1.288(6), 1.459(7), and 1.362(7) Å, respectively, comparable to those observed in (^*t*Bu^pyrox)Ni(Dipp)_2_.^23^ In contrast, the C_ox_–N_ox_ and C_py_–N_py_ bonds of **10**, a pyrox radical anion, are notably longer, while the C_ox_–C_py_ bond is shorter compared to those in **32**. The EPR spectrum of **32** exhibited a rhombic signal at 30 K, with g values of [2.448, 2.138, 2.070]. By comparing the bond lengths of **32** to (^*t*Bu^pyrox)Ni(Dipp)_2_ and **10** and considering the observation of a nickel(I) radical in EPR spectroscopy, we concluded that **32** is best described as a nickel-centered radical with a non-redox-active pyrox ligand.

Lastly, we employed NaBAr_24_^F^ to abstract the bromide from (^tBu^pyrox)Ni(Dipp)(Br) **33**, resulting in the formation of complex **34** using a similar synthetic protocol as in the preparation of **16** (Scheme 8B). The reaction led to a color change from maroon to amber. The ^1^H NMR spectra of **34** exhibited upfield shifts compared to those of **33** (Figure S45). HRMS analysis of **34** revealed an exact mass of 424.2081 (M-BAr_24_^F^ + H), indicating the absence of the bromide ion. In contrast, HRMS of **33** displayed a mass of 525.1379 (M + Na). Based on these findings, we assigned complex **34** as the cationic [(^*t*Bu^pyrox)Ni(Dipp)]^+^[BAr_24_^F^]^−^. Further reduction of **34** with KC_8_ yielded a dark green solution, and the corresponding EPR spectrum displayed a rhombic signal with *g* values of [2.308, 2.101, 2.057]. We assigned the structure of **35** to a three-coordinate [(^*t*Bu^pyrox)Ni(Dipp)] complex as a nickel-centered radical.

### DFT Calculations

We performed density functional theory (DFT) calculations on the electronic structures of the nickel radical complexes described in this study. The geometry optimization was found to be highly sensitive to the functional and the basis set. While the commonly used basis set, (U)B3LYP-D3/def2-TZVPP, was successful in reproducing the experimental geometries of [NiR_2_]^−^ and NiX complexes, it performed poorly with Ni–Ar and [NiX]_2_ complexes. As a result, we applied the combination of (U)B3LYP//m6-31g* for the Ni–Ar complexes.^31^ In general, the electronic structures obtained from DFT calculations are consistent with experimental data. The four-coordinate complexes were computed to be square planar with highly delocalized spin density on the ligands, and the ligand C=N bonds were elongated (Figures S75 and S77–S80). Among the three-coordinate Ni(I) complexes, the Ni(I)-halide and Ni(I)-phenyl complexes were computed to be trigonal planar with localized spin density on the Ni centers (Figures S73, S74, S76, S81, and S82).

## Discussion

**Scheme 9:**
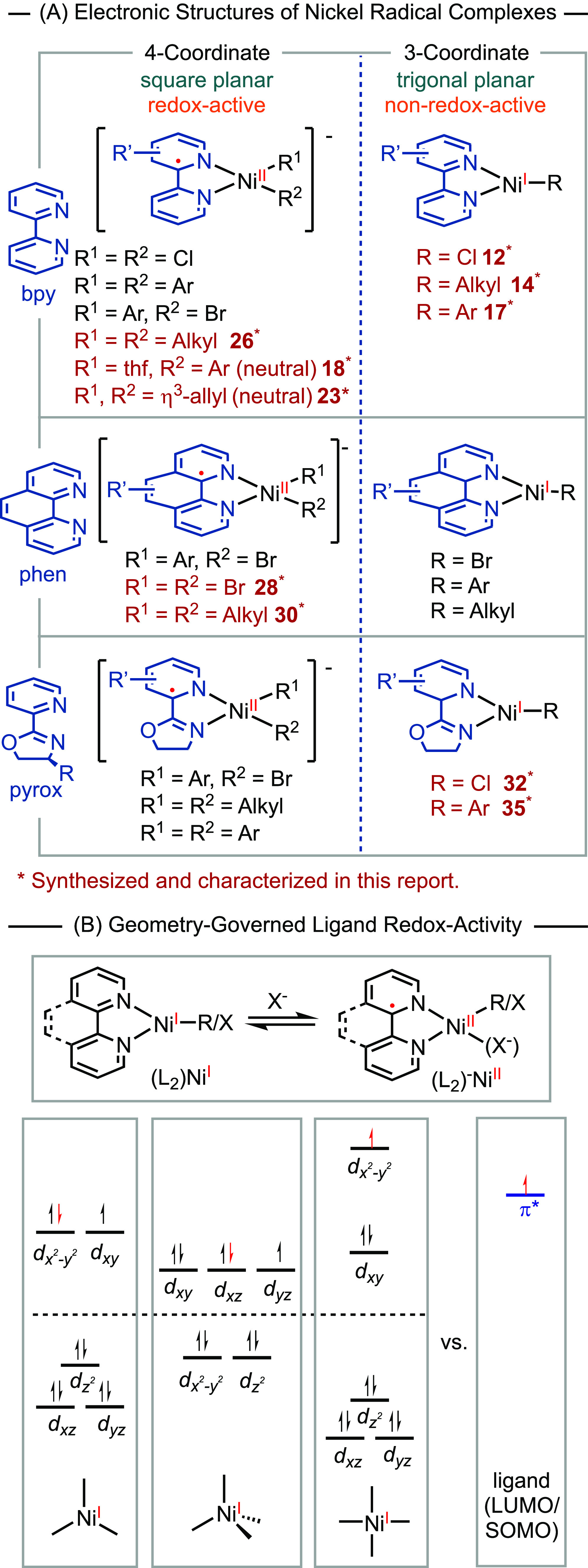
Electronic Structures of Nickel Radical Complexes (A) and the Comparison of SOMO Energy Levels in Different Geometries (B)

In this study, we synthesized a series of nickel radical complexes and characterized their electronic structures using NMR, EPR, mass spectroscopy, and X-ray crystallography. These new complexes highlighted in red in Scheme 9A, complete the range of nickel radical analogues, allowing us to draw empirical conclusions regarding the correlation between coordination geometry and redox activity of the ligand. In general, four-coordinate nickel radical complexes adopting a square-planar geometry can be best described as low-spin nickel(II) complexes coordinated with ligand radical anions. In contrast, three-coordinate nickel-halide, -alkyl, and -aryl complexes adopt trigonal planar or distorted trigonal planar geometries. These molecules are characterized as nickel(I) centers coordinated with non-redox-active ligands. This pattern remains consistent regardless of the identity of the X-ligands.

While tetrahedral nickel radical complexes are beyond the scope of this study, several such molecules have been reported within the α-diimine ligand framework, including [(α-diimine)Ni(μ-H)]_2_ and [(α-diimine)Ni(μ-halide)]_2_ complexes.^22^ In contrast to square-planar nickel complexes, most of these tetrahedral molecules are characterized as nickel-centered radicals, where the α-diimine ligand does not exhibit redox activity.

Based on the data presented in this report and literature precedents, we propose that the redox activity of bidentate *N*-ligands in low-valent organonickel radical complexes is dependent on the geometry and coordination number. The redox activity of the ligand is determined by the relative energy levels of the unfilled d orbital and the π* orbital of the ligand (Scheme 9B). Molecules of trigonal planar and tetrahedral geometries have relatively low-energy antibonding d orbitals, which can be lower than the π* orbital of the ligand. Consequently, the unpaired electron tends to occupy the d orbital, resulting in a d9 electronic configuration. In contrast, square-planar complexes have high-energy d_*x*^2^–*y*^2^_ orbitals. In this geometry, the unpaired electron prefers to occupy the π* orbital of the ligand, leading to a nickel(II) center coordinated with a radical anion ligand.

One of the most compelling pieces of evidence supporting this hypothesis is the contrasting electronic structures of **17** and **18** (Scheme 4). Introducing a coordinating solvent to shift the coordination number from three to four resulted in changes in both the redox activity of the ligand and the oxidation state of the nickel center. These results have significant implications for catalyst optimization. In nickel-catalyzed cross-coupling reactions, various additives such as MgCl_2_ and KI, have been extensively employed. Besides their role in modulating the speciation of active nickel catalysts, which has recently been elucidated,^26^ the presence of coordinating anions may also play a crucial role in stabilizing nickel(I) intermediates and tuning the redox potentials by inducing ligand redox activity. Furthermore, our data suggest that the use of coordinating solvents may benefit the reaction by exerting a similar stabilization effect.

An exception to this postulate has been reported before: a square-planar (terpy)Ni-methyl complex exhibits a ligand-centered radical, whereas square-planar (terpy)Ni-bromide^31^ and (terpy)Ni-phenolate^32^ complexes display metal-centered radicals. This observation may be attributed to various factors, including a relatively low-lying d_x2-y2_ orbital with weak-field ligands, the rigid geometry of terpy that precludes alternative geometries other than square-planar, or potential π-stacking effects caused by the planar terpy ligand. Ongoing research endeavors aim to further elucidate the underlying factors governing ligand redox activity of tridentate ligands.

## Conclusions

We have synthesized and characterized a series of nickel radical complexes that hold significant catalytic relevance. This comprehensive collection of complexes includes various (bpy), (phen), and (pyrox)nickel radical analogues, which enable us to establish a clear correlation between the coordination geometry and the redox activity of the ligands. Specifically, the four-coordinate square-planar nickel radical complexes are low-spin nickel(II) centers coordinated with radical anion ligands. In contrast, the three-coordinate nickel radical complexes exhibit a trigonal planar geometry, featuring nickel(I) centers coordinated with ligands that do not display redox activity. This trend remains consistent regardless of the identity of the X-ligands. These findings provide an account for the important role of coordinating salt additives and solvents in modulating the stability and redox potentials of nickel intermediates by altering the coordination number and inducing ligand redox activity. This understanding of the relationship between the coordination environment and ligand redox properties is crucial for the design and optimization of catalysts in the development of nickel-catalyzed cross-coupling reactions.

## Supporting Information Available

Detailed experimental procedures; characterization data, such as NMR, EPR, and UV–Visible spectra; details of DFT calculations; and spin density plots (PDF)

## References

[ref1] Hu X. Nickel-catalyzed cross coupling of non-activated alkyl halides: A mechanistic perspective. Chem. Sci. 2011, 2, 1867–1886. 10.1039/c1sc00368b.

[ref2] Tasker S. Z.; Standley E. A.; Jamison T. F. Recent advances in homogeneous nickel catalysis. Nature 2014, 509, 299–309. 10.1038/nature13274. 24828188 PMC4344729

[ref3] Choi J.; Fu G. C. Transition metal–catalyzed alkyl-alkyl bond formation: Another dimension in cross-coupling chemistry. Science 2017, 356, eaaf7230 10.1126/science.aaf7230. 28408546 PMC5611817

[ref4] Fu G. C. Transition-Metal Catalysis of Nucleophilic Substitution Reactions: A Radical Alternative to S_N_1 and S_N_2 Processes. ACS Cent. Sci. 2017, 3, 692–700. 10.1021/acscentsci.7b00212. 28776010 PMC5532721

[ref5] Diccianni J. B.; Diao T. Mechanisms of Nickel-Catalyzed Cross-Coupling Reactions. Trends Chem. 2019, 1, 830–844. 10.1016/j.trechm.2019.08.004.

[ref6] Diccianni J.; Lin Q.; Diao T. Mechanisms of Nickel-Catalyzed Coupling Reactions and Applications in Alkene Functionalization. Acc. Chem. Res. 2020, 53, 906–919. 10.1021/acs.accounts.0c00032. 32237734 PMC7958188

[ref7] Lin C.-Y.; Power P. P. Complexes of Ni(I): A ″rare″ oxidation state of growing importance. Chem. Soc. Rev. 2017, 46, 5347–5399. 10.1039/C7CS00216E. 28675200

[ref8] Zimmermann P.; Limberg C. Activation of Small Molecules at Nickel(I) Moieties. J. Am. Chem. Soc. 2017, 139, 4233–4242. 10.1021/jacs.6b12434. 28170243

[ref9] Bismuto A.; Finkelstein P.; Müller P.; Morandi B. The Journey of Ni(I) Chemistry. Helv. Chim. Acta 2021, 104, e2100177 10.1002/hlca.202100177.

[ref10] aCherney A. H.; Kadunce N. T.; Reisman S. E. Enantioselective and Enantiospecific Transition-Metal-Catalyzed Cross-Coupling Reactions of Organometallic Reagents To Construct C–C Bonds. Chem. Rev. 2015, 115, 9587–9652. 10.1021/acs.chemrev.5b00162. 26268813 PMC4566132

[ref11] Chan A. Y.; Perry I. B.; Bissonnette N. B.; Buksh B. F.; Edwards G. A.; Frye L. I.; Garry O. L.; Lavagnino M. N.; Li B. X.; Liang Y.; Mao E.; Millet A.; Oakley J. V.; Reed N. L.; Sakai H. A.; Seath C. P.; MacMillan D. W. C. Metallaphotoredox: The Merger of Photoredox and Transition Metal Catalysis. Chem. Rev. 2022, 122, 1485–1542. 10.1021/acs.chemrev.1c00383. 34793128 PMC12232520

[ref12] aTing S. I.; Williams W. L.; Doyle A. G. Oxidative Addition of Aryl Halides to a Ni(I)-Bipyridine Complex. J. Am. Chem. Soc. 2022, 144, 5575–5582. 10.1021/jacs.2c00462. 35298885

[ref13] aMohadjer Beromi M.; Banerjee G.; Brudvig G. W.; Hazari N.; Mercado B. Q. Nickel(I) Aryl Species: Synthesis, Properties, and Catalytic Activity. ACS Catal. 2018, 8, 2526–2533. 10.1021/acscatal.8b00546. 30250755 PMC6150472

[ref14] aWeix D. J. Methods and Mechanisms for Cross-Electrophile Coupling of Csp^2^ Halides with Alkyl Electrophiles. Acc. Chem. Res. 2015, 48, 1767–1775. 10.1021/acs.accounts.5b00057. 26011466 PMC4484513

[ref15] Lin Q.; Diao T. Mechanism of Ni-Catalyzed Reductive 1,2-Dicarbofunctionalization of Alkenes. J. Am. Chem. Soc. 2019, 141, 17937–17948. 10.1021/jacs.9b10026. 31589820 PMC7058187

[ref16] Lin Q.; Spielvogel E. H.; Diao T. Carbon-centered radical capture at nickel(II) complexes: Spectroscopic evidence, rates, and selectivity. Chem 2023, 9, 1295–1308. 10.1016/j.chempr.2023.02.010. PMC12333529 40786745

[ref17] Anderson T. J.; Jones G. D.; Vicic D. A. Evidence for a NiI Active Species in the Catalytic Cross-Coupling of Alkyl Electrophiles. J. Am. Chem. Soc. 2004, 126, 8100–8101. 10.1021/ja0483903. 15225035

[ref18] MohadjerBeromi M.; Brudvig G. W.; Hazari N.; Lant H. M. C.; Mercado B. Q. Synthesis and Reactivity of Paramagnetic Nickel Polypyridyl Complexes Relevant to C(sp^2^)–C(sp^3^)Coupling Reactions. Angew. Chem., Int. Ed. 2019, 58, 6094–6098. 10.1002/anie.201901866. PMC6479119 30859704

[ref19] Klein A.; Kaiser A.; Sarkar B.; Wanner M.; Fiedler J. The Electrochemical Behaviour of Organonickel Complexes: Mono-, Di- and Trivalent Nickel. Eur. J. Inorg. Chem. 2007, 2007, 965–976. 10.1002/ejic.200600865.

[ref20] Irwin M.; Doyle L. R.; Krämer T.; Herchel R.; McGrady J. E.; Goicoechea J. M. A Homologous Series of First-Row Transition-Metal Complexes of 2,2′-Bipyridine and their Ligand Radical Derivatives: Trends in Structure, Magnetism, and Bonding. Inorg. Chem. 2012, 51, 12301–12312. 10.1021/ic301587f. 23110751

[ref21] Schley N. D.; Fu G. C. Nickel-Catalyzed Negishi Arylations of Propargylic Bromides: A Mechanistic Investigation. J. Am. Chem. Soc. 2014, 136, 16588–16593. 10.1021/ja508718m. 25402209 PMC4277758

[ref22] aShao Q.; Sun H.; Shen Q.; Zhang Y. Bis{[diacetyl-bis(2,6-isopropylphenylimine)]nickel(I)(μ-chloro)}. Appl. Organomet. Chem. 2004, 18, 289–290. 10.1002/aoc.624.

[ref23] Wagner C. L.; Herrera G.; Lin Q.; Hu C. T.; Diao T. Redox activity of Pyridine-Oxazoline Ligands in the Stabilization of Low-Valent Organonickel Radical Complexes. J. Am. Chem. Soc. 2021, 143, 5295–5300. 10.1021/jacs.1c00440. 33792294 PMC8851433

[ref24] aLu C. C.; Bill E.; Weyhermüller T.; Bothe E.; Wieghardt K. Neutral Bis(α-iminopyridine)metal Complexes of the First-Row Transition Ions (Cr, Mn, Fe, Co, Ni, Zn) and Their Monocationic Analogues: Mixed Valency Involving a Redox Noninnocent Ligand System. J. Am. Chem. Soc. 2008, 130, 3181–3197. 10.1021/ja710663n. 18284242

[ref25] Ju L.; Lin Q.; LiBretto N. J.; Wagner C. L.; Hu C. T.; Miller J. T.; Diao T. Reactivity of (bi-Oxazoline)organonickel Complexes and Revision of a Catalytic Mechanism. J. Am. Chem. Soc. 2021, 143, 14458–14463. 10.1021/jacs.1c07139. 34463481 PMC8883516

[ref26] Day C. S.; Rentería-Gómez Á.; Ton S. J.; Gogoi A. R.; Gutierrez O.; Martin R. Elucidating electron-transfer events in polypyridine nickel complexes for reductive coupling reactions. Nat. Catal. 2023, 6, 244–253. 10.1038/s41929-023-00925-4. PMC11546168 39525327

[ref27] Humphrey E. L. B. J.; Kennedy A. R.; Sproules S.; Nelson D. J. Evaluating a Dispersion of Sodium in Sodium Chloride for the Synthesis of Low-Valent Nickel Complexes. Eur. J. Inorg. Chem. 2022, 2022, e202101006 10.1002/ejic.202101006.

[ref28] Sun R.; Qin Y.; Ruccolo S.; Schnedermann C.; Costentin C.; Nocera D. G. Elucidation of a Redox-Mediated Reaction Cycle for Nickel-Catalyzed Cross Coupling. J. Am. Chem. Soc. 2019, 141, 89–93. 10.1021/jacs.8b11262. 30563318

[ref29] Yakhvarov D. G.; Petr A.; Kataev V.; Büchner B.; Gómez-Ruiz S.; Hey-Hawkins E.; Kvashennikova S. V.; Ganushevich Y. S.; Morozov V. I.; Sinyashin O. G. Synthesis, structure and electrochemical properties of the organonickel complex [NiBr(Mes)(phen)] (Mes = 2,4,6-trimethylphenyl, phen = 1,10-phenanthroline). J. Organomet. Chem. 2014, 750, 59–64. 10.1016/j.jorganchem.2013.11.003.

[ref30] Somerville R. J.; Odena C.; Obst M. F.; Hazari N.; Hopmann K. H.; Martin R. Ni(I)–Alkyl Complexes Bearing Phenanthroline Ligands: Experimental Evidence for CO2 Insertion at Ni(I) Centers. J. Am. Chem. Soc. 2020, 142, 10936–10941. 10.1021/jacs.0c04695. 32520556 PMC7351122

[ref31] Ciszewski J. T.; Mikhaylov D. Y.; Holin K. V.; Kadirov M. K.; Budnikova Y. H.; Sinyashin O.; Vicic D. A. Redox Trends in Terpyridine Nickel Complexes. Inorg. Chem. 2011, 50, 8630–8635. 10.1021/ic201184x. 21797263

[ref32] Bismuto A.; Müller P.; Finkelstein P.; Trapp N.; Jeschke G.; Morandi B. One to Find Them All: A General Route to Ni(I)–Phenolate Species. J. Am. Chem. Soc. 2021, 143, 10642–10648. 10.1021/jacs.1c03763. 34251813

[ref33] Laskowski C. A.; Bungum D. J.; Baldwin S. M.; Del Ciello S. A.; Iluc V. M.; Hillhouse G. L. Synthesis and Reactivity of Two-Coordinate Ni(I) Alkyl and Aryl Complexes. J. Am. Chem. Soc. 2013, 135, 18272–18275. 10.1021/ja4095236. 24237257

[ref34] Lin Q.; Dawson G.; Diao T. Experimental Electrochemical Potentials of Nickel Complexes. Synlett 2021, 32, 1606–1620. 10.1055/s-0040-1719829.

[ref35] Sandleben A.; Vogt N.; Hörner G.; Klein A. Redox Series of Cyclometalated Nickel Complexes [Ni((R)Ph(R′)bpy)Br]^+/0/–/2^– (H–(R)Ph(R′)bpy = Substituted 6-Phenyl-2,2′-bipyridine). Organometallics 2018, 37, 3332–3341. 10.1021/acs.organomet.8b00559.

