# Supplementary material for: Ligand Redox Activity of Organonickel Radical Complexes Governed by the Geometry
Source: J Am Chem Soc. 2023 Sep 11;145(37):20551–61. doi: 10.1021/jacs.3c07031 (PMC10515493; doi:10.1021/jacs.3c07031)
Supplement: Supplementary file 1 — ja3c07031_si_001.pdf [file ja3c07031_si_001.pdf]

# Supporting Information

## Ligand Redox Activity of Organonickel Radical Complexes Governed by the Geometry

Gregory A. Dawson,[1] Qiao Lin,[1] Michelle C. Neary,[2] and Tianning Diao*

[1]Department of Chemistry, New York University, 100 Washington Square East, New York, NY 10003

[2]Department of Chemistry, CUNY – Hunter College, 695 Park Ave NY, NY 10065

*E-Mail: diao@nyu.edu

# Table of Contents

1. General Considerations ........ S3

2. Tabulated Comparison of Reported Ni(I) Complexes........ S4

3. Synthesis and Characterization of Ligands and Nickel Complexes........ S5

4. UV-Vis Spectra........ S23

5. NMR Spectra........ S32

6. EPR Spectra........ S47

7. Single-Crystal Structure Determination ........ S56

8. DFT Calculations........ S62

9. References........ S81

## 1. General Considerations

All air- and moisture-sensitive manipulations were carried out in a glove box. Solvents were dried and deoxygenated by a Grubbs' type solvent purification system. Benzene-$d_6$ and chloroform-*d* were dried over 4Å molecular sieves, filtered, and degassed using the freeze-pump-thaw method. Metals, ligands, and most substrates were obtained from commercial sources. $NiCl_2$•dme[1], $NiBr_2$•dme[1], $TMSCH_2MgCl$[2], DippBr[3] (Dipp = 2,6-diisopropylphenyl), Dipp*Li[4] (2,6-bis-Dipp-phenyl), $^{tBu}$pyrox[5] ($^{tBu}$pyrox = (S)-4-(*tert*-butyl)-2-(2-pyridyl)-4,5-dihydrooxazole), and dibenzyl glycinol[6] were synthesized via modified literature procedures.

$^1$H spectra were collected on Bruker 400, 500, and 600 MHz Avance Spectrometers. Chemical shifts are reported in ppm relative to tetramethylsilane, with the residual solvent resonances (benzene-$d_6$, $\delta$ = 7.16 ppm; chloroform-*d*, $\delta$ = 7.26 ppm; acetonitrile-$d_3$, $\delta$ = 1.94 ppm; acetone-$d_6$, $\delta$ = 2.05 ppm) as the internal reference for $^1$H NMR spectra. Spectra are reported as follows: chemical shift ($\delta$ ppm), multiplicity (bs = broad singlet, s = singlet, d = doublet, t = triplet, q = quartet, m = multiplet), coupling constant (Hz), and integration. $^{13}$C NMR spectra were collected on Bruker 400 and 500 MHz Avance Spectrometers. Chemical shifts are reported in ppm relative to tetramethylsilane, with the residual solvent resonances (benzene-$d_6$, $\delta$ = 128.06 ppm; chloroform-*d*, $\delta$ = 77.16 ppm) as the internal reference for $^{13}$C NMR spectra.

High resolution mass spectra (HRMS) were recorded on an Agilent 6224 TOF LC/MS (APCI source). UV-Vis spectra were collected on a Cary 100 Bio UV-Vis Spectrophotometer. Single crystal diffraction data were recorded on a Bruker D8 APEX-II system, (Bruker AXS Inc., Madison, WI, USA) either using graphite-monochromated and 0.5 mm MonoCap-collimated Mo K$\alpha$ radiation generated from a Siemens sealed tube and a CCD detector or Montel optics-collimated Mo-K$\alpha$ radiation ($\lambda$ = 0.71073 Å) generated from an Incotec microfocus source and a PHOTON II C14 detector, or a Bruker D8 VENTURE, using Mo K$\alpha$ or Cu K$\alpha$ radiation. EPR measurements were taken on a Bruker ELEXSYS E500 Spectrometer System in continuous-wave mode in the X-band frequency using a Bruker ER 4122SHQE resonator. The spectra were simulated with Easyspin in MATLAB or Xepr software. All DFT calculations were carried out using the Gaussian 16 program[7] or the ORCA 4.2.1[8] on NYU GREENE supercomputers. Vibrational frequency calculations were performed for all stationary points to confirm if each optimized structure is a local minimum.

## 2. Tabulated Comparison of Reported Ni(I) Complexes

### Table S1. Characterization data of reported nickel radical complexes and ligand redox activity

| Complex | Location of the radical | EPR g value | $C_{imine}$–$N_{imine}$ (Å) | $C_{\alpha\text{-imine}}$–$C_{\alpha\text{-imine}}$ (Å) |
|---|---|---|---|---|
| [(dtbpy)Ni(μ-Cl)]$_2$ **1**[9] | Ligand | - | 1.349(7), 1.349(6); 1.344(6), 1.344(6) | 1.457(8); 1.457(8) |
| [(bpy)Ni(Mes)(Br)]$^-$ **2**[10] | Ligand | 2.005 | - | - |
| [(bpy)Ni(Mes)$_2$]$^-$[K(2,2,2-crypt]$^+$ **3**[11] | Ligand | 2.010 | 1.390(2), 1.390(2) | 1.416(4) |
| ($^{Me}$bpy)NiCl **4**[12] | Metal | 2.213[a] | 1.355(3), 1.358(3) | 1.484(3) |
| [(3,4,7,8-tetramethyl-phen)Ni(Mes)(Br)]$^-$ **5**[10] | Ligand | 1.999 | - | - |
| [(3,4,7,8-tetramethyl-phen)Ni(Mes)$_2$]$^-$ [10] | Ligand | 2.007 | - | - |
| ($^{sBu}$phen)NiBr **6**[13] | Metal | 2.478, 2.120, 2.087 | 1.364(5), 1.361(6) | 1.427(6) |
| ($^{Mes}$phen)Ni($CH_2C(CH_3)_3$) **8**[14] | Metal | 2.519, 2.145, 2.065 | 1.370(4), 1.371(4) | 1.422(5) |
| ($^{Me}$phen)Ni(2,4,6-tri-isopropylphenyl) **7**[9] | Metal | 2.510, 2.130, 2.060 | 1.379(7), 1.367(8); 1.374(8), 1.361(8) | 1.423(8); 1.423(8) |
| [($^{tBu}$pyrox)Ni($CH_2$TMS)$_2$]$^-$[K(18-crown-6]$^+$ **9**[15] | Ligand | 2.009 | 1.311(7), 1.402(8) | 1.393(8) |
| [($^{tBu}$pyrox)Ni(Dipp)$_2$]-[K(18-crown-6]$^+$ **10**[15] | Ligand | 2.007 | 1.317(12), 1.378(13) | 1.423(14) |
| ($^{dBn}$biox)NiBr[16] | Metal | 2.486, 2.175, 2.062 | 1.273(6), 1.285(6)[b] | 1.438(7)[b] |
| ($^{dMe}$biox)Ni(Dipp)[16] | Metal | 2.492, 2.179, 2.070 | - | - |
| ($^{Dipp}$ADI)Ni(η-3 Bn)[17] | Ligand | 2.003 | 1.340(17), 1.346(17) | 1.411(18) |
| [($^{Dipp}$ADI)Ni(μ-H)]$_2$[18] | Ligand | 2.007 | 1.313(4), 1.318(4); 1.327(5), 1.323(5) | 1.438(5); 1.421(6) |
| ($^{ipc}$ADI)NiCl[19] | Metal | 2.203 | 1.285(10), 1.295(90 | 1.485(10) |
| ($^{ipc}$ADI)NiBr[19] | Metal | 2.212 | 1.289(7), 1.299(6) | 1.480(7) |
| ($^{ipc}$ADI)NiI[19] | Metal | 2.310, 2.205, 2.135 | 1.302(9), 1.301(9) | 1.478(9) |
| (terpy)Ni($CH_3$)[20] | Ligand | 2.021 | 1.39(3), 1.34(3), 1.37(3), 1.32(2) | 1.51(3), 1.44(3) |
| (terpy)NiBr[21] | Metal | 2.139 | 1.37(2), 1.328(18), 1.328(18), 1.37(2) | 1.46(2), 1.46(2) |
| ($^{tBu}$terpy)NiI[22] | Metal | - | 1.359(4), 1.349(4), 1.345(3), 1.346(4) | 1.487(4), 1.483(4) |
| (terpy)Ni(2,4,6-tri-*tert*-butylphenol)[23] | Metal | 2.052, 2.090, 2.207 | 1.361(6), 1.350(6), 1.352(6), 1.368(6) | 1.462(7), 1.465(7) |
| ($^{iPr}$pybox)Ni(Ph)[24] | Ligand | 2.007, 2.008, 1.989 | 1.318(4), 1.374(4), 1.348(4), 1.293(4); 1.307(4), 1.377(4), 1.345(3), 1.298(4) | 1.416(4), 1.469(4); 1.427(4), 1.448(4) |

[a] EPR measurement taken at 293 K. [b] Bond lengths taken from isolated dimeric species.

## 3. Synthesis and Characterization of Ligands and Nickel Complexes

6,6'-dicyclohexyl-2,2'-bipyridine ($^{Cy}$bpy) **S1**

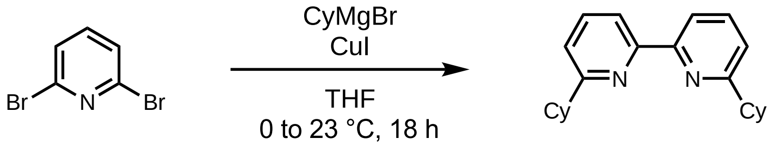


**S1**
5%

**S1** was isolated from a reported literature procedure.[25] A Schlenk flask was charged with CuI (0.10 g, 0.53 mmol, 0.05 equiv.) under a $N_2$ atmosphere. Under a positive pressure of $N_2$, 2,6-dibromopyridine (2.5 g, 11 mmol, 1 equiv.) and anhydrous THF (6.7 mL, 1.65M) were added to the flask then the mixture was cooled to 0 ºC. A solution of cyclohexylmagnesium bromide (7.2 mL, 21 mmol, 2 equiv., 2.95 M in THF) was added dropwise over an hour while maintaining cold temperature and allowed to stir overnight with warming to room temperature.

The reaction was neutralized with a saturated aqueous solution of $NH_4Cl$ (20 mL) then additional EtOAc (30 mL) was added, and organic phase collected. The aqueous phase was extracted with EtOAc (2 x 15 mL) and the combined organic extracts were washed with aqueous $NH_3$ (1 x 15 mL). After drying over $MgSO_4$ and removing solvent under reduced pressure, the crude residue was purified by flash column chromatography (hexanes/EtOAc, 0 to 5% EtOAc) to afford an off-white crystalline solid. Further purification by recrystallization from a concentrated hexanes solution at -35 °C delivered colorless crystalline solids (166 mg, 5% yield).

**$^1$H NMR (500 MHz, chloroform-*d*):** δ 8.27 (d, *J* = 7.7 Hz, 2H), 7.70 (t, *J* = 7.7 Hz, 2H), 7.13 (d, *J* = 7.7 Hz), 2.76 (t, *J* = 11.8 Hz, 2H), 2.02 (d, *J* = 12.1 Hz, 4H), 1.87 (d, *J* = 12.8 Hz, 4H), 1.77 (d, *J* = 12.3 Hz, 2H), 1.61 (q, *J* = 12.4 Hz, 4H), 1.44 (q, *J* = 12.7 Hz, 4H), 1.31 (q, *J* = 12.7 Hz, 2H).

**$^{13}$C NMR (126 MHz, chloroform-*d*):** δ 165.8, 156.0, 137.0, 120.8, 118.4, 46.6, 33.1, 26.77, 26.36, 1.1.

**HRMS (ESI-TOF):** calculated for $[C_{22}H_{28}N_2 + Na]^+$: 343.2145, found: 343.2126.

($^{Cy}$bpy)$NiCl_2$ **11**

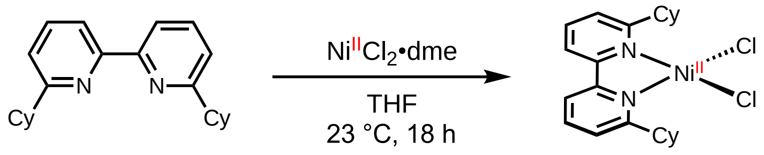


**S1**

**11**
*pale pink*
90%

In a nitrogen-filled glovebox, to a 20 mL scintillation vial was added **S1** (29.0 mg, 0.090 mmol, 1 equiv.), $NiCl_2$•dme (21.9 mg, 0.099 mmol, 5 equiv.), and THF (5 mL, 0.02M) then stirred at room temperature for 4 hours. The solution gradually turned light pink. After allowing excess $NiCl_2$•dme to settle, the suspension was filtered through a celite plug and solvent removed under reduced

pressure. The solids were washed with $Et_2O$ (3 x 3 mL) and triturated with pentane (3 x 3 mL) to afford **11** as a pale pink powder (36.5 mg, 90% yield).

**$^1$H NMR (400 MHz, acetone-*d*$_6$):** δ 78.96 (s, 1H), 63.79 (s, 1H), 24.66 (s, 2H), 23.23 (s, 1H), 16.12 (s, 2H), 7.07 (bs, 2H), 5.53 (s, 1H), 5.35 (s, 2H), 5.19 (d, *J* = 10.9 Hz, 2H), 2.80 (d, *J* = 13.4 Hz, 3H). This compound shows no $^{13}$C NMR spectrum.

**UV-Vis (λ(ε), THF, 23 °C):** 493 nm (88 $M^{-1}cm^{-1}$).

($^{Cy}$bpy)NiCl **12**

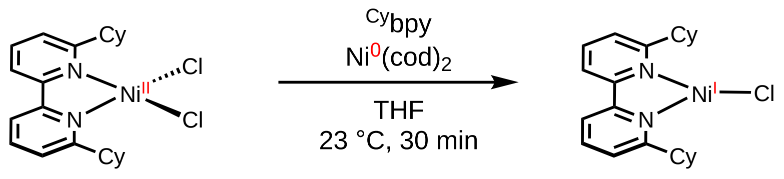


**11**
*pale pink*

**12**
*emerald green*
98%

**12** was synthesized following a reported literature procedure for an analogous compound.[19] In a nitrogen-filled glovebox, to a 20 mL scintillation vial was added **11** (10.0 mg, 0.022 mmol, 1 equiv.), $^{Cy}$bpy (7.1 mg, 0.022 mmol, 1 equiv.), and THF (3 mL, 0.01M). Separately, a solution of $Ni(cod)_2$ (6.1 mg, 0.022 mmol, 1 equiv.) in 2 mL of THF was prepared. After stirring the solution of **11** for 5 minutes, the $Ni(cod)_2$ suspension was added in one portion and the reaction was allowed to stir for 30 minutes at room temperature. After filtering through a celite plug, solvent was removed under reduced pressure and the crude residue was washed with pentane (3 x 3 mL) to afford dark green microcrystalline solids (18.0 mg, 98% yield). Dark green crystals suitable for XRD were obtained from a concentrated solution of THF layered with pentane stored in a -35 °C freezer overnight.

**$^1$H NMR (400 MHz, benzene-*d*$_6$):** δ 75.14 (s, 1H), 61.11 (s, 1H), 23.32 (s, 3H), 21.43 (s, 2H), 15.59 (s, 3H). This compound shows no $^{13}$C NMR spectrum.

**UV-Vis (λ, THF, 23 °C):** 247 nm, 297 nm, 320 nm, 382 nm, 668 nm.

($^{sBu}$bpy)NiBr2 ($^{sBu}$bpy = 6,6'-di-*sec*-butyl-2,2'-bipyridine) **S2**

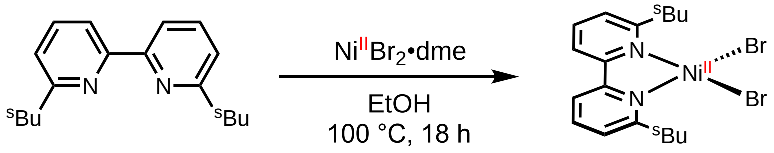


**S2**
*bright pink*
15%

**S2** was synthesized following a reported literature procedure for an analogous compound.[13] To a flame dried round bottom flask fitted with a reflux condenser was added $^{sBu}$bpy (80.0 mg, 0.30 mmol, 1 equiv.) and EtOH (50 mL, 6 mM), followed by purging with $N_2$. $NiBr_2$•dme (276 mg, 0.89 mmol, 3 equiv.) was added to this mixture at room temperature, then stirred overnight at reflux. After cooling to room temperature, pink solid precipitates are collected via vacuum filtration then washed several times with hexanes to afford **S2** as a bright pink powder (22 mg, 15% yield).

($^{sBu}$bpy)NiBr **S3**

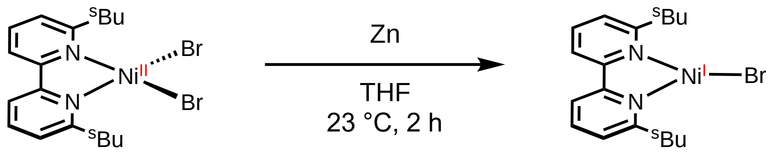


**S2**
*pale pink*

**S3**
*emerald green*
obsevred spectroscopically by EPR

In a nitrogen-filled glovebox, to a 20 mL scintillation vial was added **S2 (**6.5 mg, 0.013 mmol, 1 equiv.) and Zn (43 mg, 0.66 mmol, 50 equiv.) then suspended in THF (2 mL, 6.5 mM). This heterogenous mixture was stirred for 2 hours at room temperature and the solution gradually turned emerald green. The mixture was filtered through a celite plug and used for EPR analysis. Attempts to recrystallize this complex from a concentrated solution of $Et_2O$ at -35 °C resulted in decomposition and isolation of XRD quality crystals of complex **S2**.

4,4'-di-*tert*-butyl-6,6'-dichloro-2,2'-bipyridine ($^{Cl}$dtbpy) **S4**

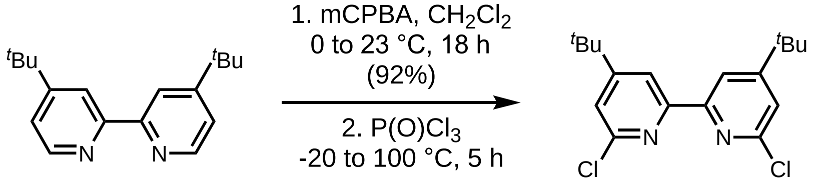


**S4**
32%

**S4** was synthesized following reported literature procedures.[26] A flame dried round bottom flask was charged with 4,4'-di-*tert*-butyl-2,2'-bipyridine (2.17 g, 8.08 mmol, 1 equiv.) and *meta*-chloroperoxybenzoic acid (5.58 g, 32.3 mmol, 4 equiv.), followed by purging with $N_2$. The flask was cooled in a 0 °C ice bath for 15 minutes then anhydrous dichloromethane (50 mL, 0.16M) was added and the reaction was allowed to stir overnight while warming to room temperature. An aqueous solution of 2 M KOH (100 mL, 0.08 M) was added to the reaction mixture then the organic phase was extracted with dichloromethane (3 x 20 mL). The combined organic extracts were dried over $MgSO_4$ and filtered. Solvent was removed under reduced pressure to afford a yellow solid (2.20 g, 92% yield) that was taken to the next step without further purification.

4,4'-di-*tert*-butyl-2,2'-bipyridine *N*,*N'*-dioxide (2.20 g, 7.3 mmol, 1 equiv.) was added to another flame dried round bottom flask, atmosphere purged with $N_2$, and cooled to -20 °C. Phosphoryl

chloride (5.30 mL, 57 mmol, 7.8 equiv.) was added dropwise and the reaction was warmed to room temperature. The solution was then heated to 100 °C for 3 hours. After cooling to room temperature, the volatiles were removed by vacuum distillation then the remaining crude oil was neutralized with an aqueous solution of 2M NaOH. The aqueous phase was then extracted with dichloromethane (3 x 40 mL), and the combined organic extracts dried over $MgSO_4$. After filtration and removal of solvent under reduced pressure, colorless crystalline solids (800 mg, 32% yield) were obtained after recrystallization from a concentrated solution of hexanes stored in a -20 °C freezer overnight. $^1$H NMR data agrees with reported literature.[26b]

4,4'-di-*tert*-butyl-6,6'-di-mesityl-2,2'-bipyridine ($^{Mes}$dtbpy) **S5**

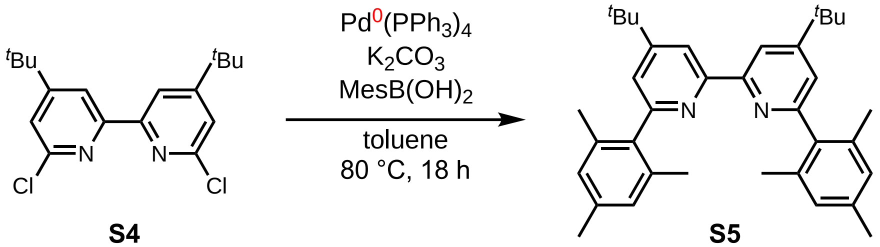


**S5** was synthesized following a modified literature procedure for an analogous compound.[27] **S4** (505.0 mg, 1.50 mmol, 1 equiv.) 2,4,6-trimethylphenylboronic acid (589.3 mg, 3.59 mmol, 2.4 equiv.), and $Pd(PPh_3)_4$ (173.0 mg, 0.15 mmol, 0.1 equiv.) were added to a flame dried flask under a $N_2$ atmosphere then suspended in anhydrous toluene (30 mL, 0.05 M). Concurrently, a freshly prepared aqueous $K_2CO_3$ solution (12.7 mL, 2 M) was bubbled with $N_2$ for 15 minutes then added to the reaction mixture. The reaction was stirred at 80 °C for 18 hours. After cooling to room temperature, the organic layer was separated, and aqueous layer extracted with dichloromethane (2 x 40 mL). The combined organic extracts were dried over $MgSO_4$, filtered, and solvent removed under reduced pressure. The compound was purified by flash column chromatography (10:1, hexanes:EtOAc) to afford **S5** as an off-white solid (490 mg, 65% yield).

**$^1$H NMR (500 MHz, chloroform-*d*):** δ 8.58 (d, $J$ = 1.6 Hz, 2H), 7.49 (d, $J$ = 1.6 Hz, 2H), 2.63 (s, 6H), 2.42 (s, 12H), 1.61 (s, 18H).

**$^{13}$C NMR (126 MHz, chloroform-*d*):** δ 160.7, 158.7, 156.9, 138.7, 137.3, 136.3, 128.5, 122.1, 116.5, 35.1, 30.8, 21.2, 20.7.

**HRMS (ESI-TOF):** calculated for $[C_{36}H_{44}N_2 + H]^+$: 505.3577, found: 505.3471.

($^{Mes}$dtbpy)$NiBr_2$ **S6**

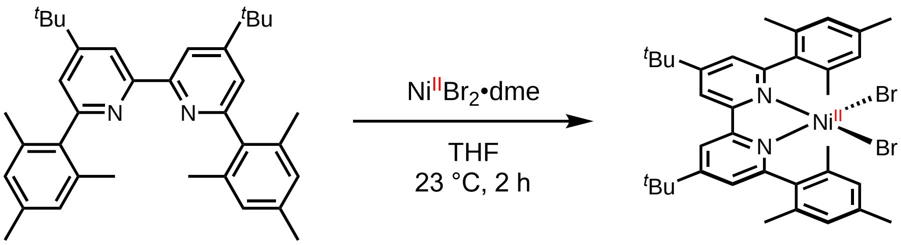


**S5**

**S6**
*magenta*
98%

**S6** was synthesized following the same reported procedure as ($^{Cy}$bpy)$NiCl_2$ **11**, instead using 1 equivalent of $NiBr_2$•dme and was only stirred for 2 hours at room temperature. **S6** was isolated as a magenta pink powder (27 mg, 75% yield).

**$^1$H NMR (400 MHz, acetone-*d*$_6$):** δ 67.95 (s, 2H), 56.51 (s, 2H), 9.69 (s, 4H), 5.28 (s, 6H), 4.95 (bs, 10H), 3.01 (bs, 4H), 1.83 (s, 18H). This compound shows no $^{13}$C NMR spectrum.

**UV-Vis (λ(ε), THF, 23 °C):** 512 nm (174 $M^{-1}cm^{-1}$).

($^{Mes}$dtbpy)NiBr **13**

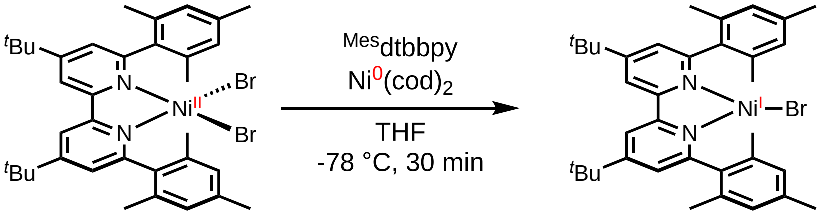


**S6**
*magenta*

**13**
*dark yellow/green*
62%

**13** was synthesized following the same reported procedure as ($^{Cy}$bpy)NiCl **12**, instead preparing and keeping the reaction at -78 °C in a coldwell. **13** was isolated as a dark green crystalline solid (22.5 mg, 62% yield). ***Note:*** *($^{Mes}$dtbpy)NiBr **13** decomposes rapidly at room temperature.*

**$^1$H NMR (400 MHz, benzene-*d*$_6$):** δ 63.89 (s, 1H), 55.09 (s, 1H), 9.28 (s, 2H), 5.29 (s, 6H), 4.88 (s, 3.5H), 1.13 (s, 12.87H). This compound shows no $^{13}$C NMR spectrum.

**UV-Vis (λ, THF, 23 °C):** 253 nm, 302 nm, 404 nm, 698 nm.

$(^{Mes}dtbpy)Ni(CH_2TMS)$ **14**

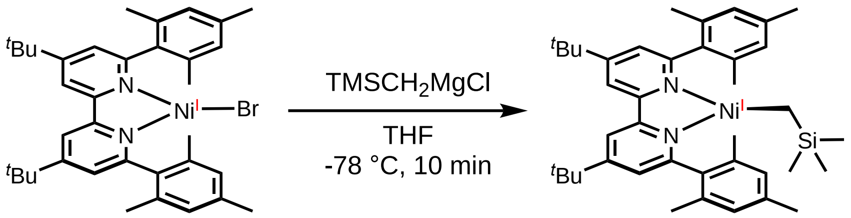


**13**
*dark green*

**14**
*orange red*
single crystals obtained

**14** was synthesized following a modified literature procedure for an analogous compound.[14] In a nitrogen-filled glovebox, to a 20 mL scintillation vial was added **13** (29.5 mg, 45.9 mmol, 1 equiv.) and THF (5 mL, 0.009 M) at -78 °C. Neosilyl magnesium chloride (119.1 μL, 0.77 M in THF, 2 equiv.) was added and the solution immediately turned dark orange. After allowing to sit in the coldwell at -78 °C for 10 minutes, the mixture was filtered through a celite plug and solvent removed under reduced pressure to obtain **14** as an orange-brown residue. Dark orange crystals suitable for XRD were obtained from a concentrated solution of $Et_2O$ layered with pentane stored in a -35 °C freezer over 3 days. This compound shows no $^1H$ or $^{13}C$ NMR spectrum. ***Note:*** *$(^{Mes}dtbpy)Ni(CH_2TMS)$* ***14*** *decomposes when stored at room temperature for prolonged periods of time.*

**UV-Vis (λ, THF, 23 °C):** 241 nm, 293 nm.

(dtbpy)Ni(Dipp)Br (dtbpy = 4,4'-di-*tert*-butyl-2,2'-bipyridine) **15**

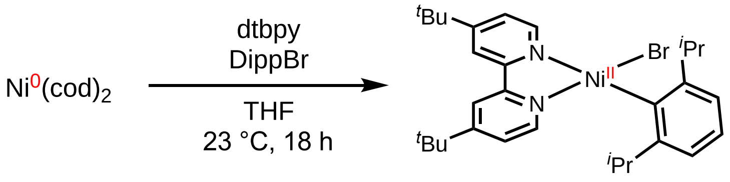


**15**
*dark red*
66%

**15** was synthesized following a reported literature procedure for an analogous compound.[28] In a nitrogen-filled glovebox, to a 20 mL scintillation vial was added $Ni(cod)_2$ (40.0 mg, 0.15 mmol, 1 equiv.), dtbpy (39.0 mg, 0.15 mmol, 1 equiv.), and THF (5 mL, 0.03 M). The solution turned dark purple while stirring over 30 minutes at room temperature. Then 1-bromo-2,6-diisopropyl benzene (175.4 mg, 0.73 mmol, 5 equiv.) was added in one portion and allowed to stir overnight at room temperature, resulting in a dark red solution. After filtering through a celite plug and removing solvent under reduced pressure, the crude residue was washed and triturated with pentane (5 x 5 mL). **15** was isolated as a dark red powder (54.5 mg, 66% yield).

**$^1$H NMR (400 MHz, chloroform-*d*):** δ 9.42 (d, *J* = 5.8 Hz, 1H), 7.80 (s, 1H), 7.77 (s, 1H), 7.48 (d, *J* = 5.8 Hz, 1H), 7.09 (q, *J* = 6.2 Hz, 2H), 6.92 (t, *J* = 7.4 Hz, 2H), 6.77 (d, *J* = 7.4 Hz, 2H), 5.69 (sept, *J* = 6.8 Hz, 2H), 1.41 (s, 9H), 1.35 (s, 9H), 1.11 (d, *J* = 6.8 Hz, 6H).

**$^{13}$C NMR (101 MHz, chloroform-*d*):** δ 163.0, 162.3, 155.8, 152.5, 151.24, 152.12, 145.2, 124.2, 123.5, 122.9, 121.2, 117.3, 116.5, 37.1, 35.5, 30.48, 30.28, 24.44.

**UV-Vis (λ(ε), THF, 23 °C):** 285 nm (19408 $M^{-1}cm^{-1}$), 487 nm (2152 $M^{-1}cm^{-1}$).

**HRMS (ESI-TOF):** calculated for $[C_{30}H_{41}N_2BrNi - Br + H]^+$: 488.2696, found: 488.2720.

$[(dtbpy)Ni(Dipp)][BAr^F_{24}]$ ($BAr^F_{24}$ = tetrakis(3,5-bis(trifluoromethyl)phenyl)borate) **16**

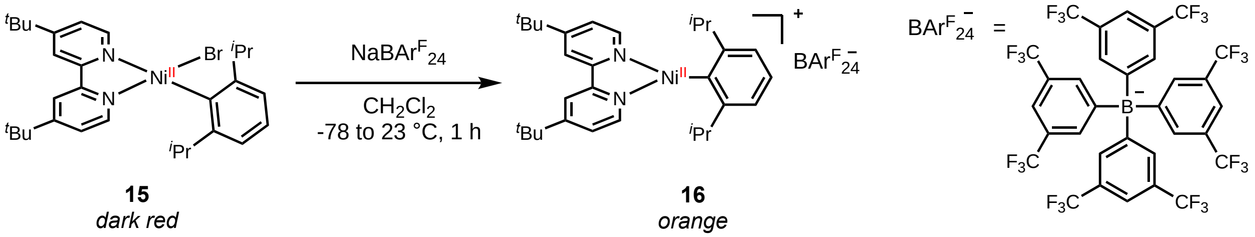


**15**
*dark red*

**16**
*orange*
66%

In a nitrogen-filled glovebox, to 2 separate 20 mL scintillation vials was added **15** (60.0 mg, 0.11 mmol, 1 equiv.) and $NaBAr^F_{24}$ (102.9 mg, 0.12 mmol. 1.1 equiv.) then both were suspended in dichloromethane (3 mL each, 0.02 M). Both vials were then placed in a coldwell cooled to – 78 °C for 15 minutes, after which the solution of $NaBAr^F_4$ was added to the solution containing the Ni complex. The reaction was left to sit at -78 °C for 1 hour, while the color gradually turned dark orange, then was brought to room temperature and filtered through a celite plug. After removing solvent under reduced pressure, the crude residue was washed and triturated with pentane (3 x 5 mL, each). **16** was isolated as a foamy dark orange solid (94.5 mg, 66% yield). ***Note*:** *If coordinating solvent (like THF) is not fully removed from starting material then the product requires extra pentane triturations to obtain a foamy solid from an oily residue. Residual coordinating solvent also broadens NMR shifts of desired product and turns the color more orange-red.*

**$^1$H NMR (500 MHz, chloroform-*d*):** δ 7.94 (d, *J* = 6.2 Hz, 2H), 7.78 (d, *J* = 1.8 Hz, 2H), 7.68 (bs, 8H), 7.53 (dd, *J* = 6.2, 1.8 Hz, 2H), 7.50 (bs, 4H), 7.23 (t, *J* = 7.6 Hz, 1H), 7.05 (m, 2H), 2.89 (sept., *J* = 6.9 Hz, 2H), 1.35 (s, 18H), 1.26 (d, *J* = 6.9 Hz, 12H). Compound decomposes when attempting to get $^{13}$C NMR.

**$^{19}$F NMR (470 MHz, chloroform-*d*):** δ -62.21.

**UV-Vis (λ(ε), THF, 23 °C):** 294 nm (21114 $M^{-1}cm^{-1}$), 488 nm (430 $M^{-1}cm^{-1}$).

**HRMS (ESI-TOF):** calculated for $[C_{62}H_{53}BF_{24}N_2Ni - C_{32}H_{12}BF_{24} + MeCN]^+$: 528.2889, found: 528.2896.

(dtbpy)Ni(Dipp) **17,18**

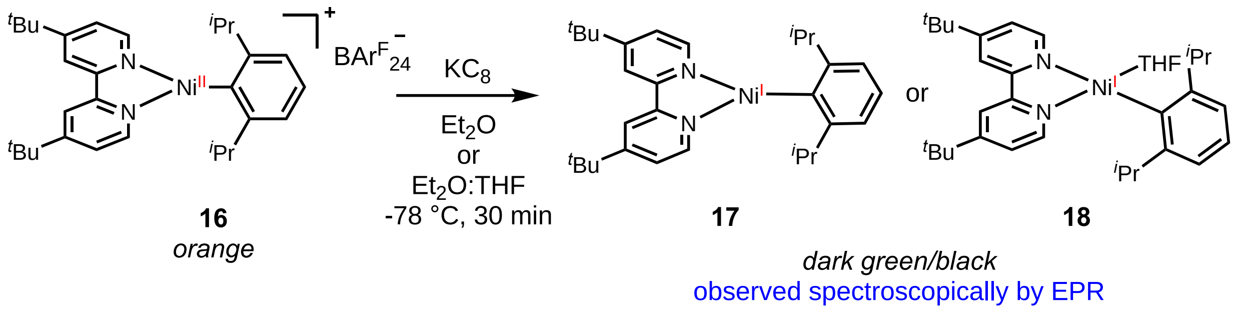


In a nitrogen-filled glovebox, to 2 separate 20 mL scintillation vials was added **16** (10.0 mg, 0.007 mmol, 1 equiv.) and $KC_8$ (3.0 mg, 0.022 mmol, 3 equiv.) then both were suspended in $Et_2O$ (1.5 mL each, 0.002M). Both vials were then placed in a coldwell cooled to – 78 °C for 15 minutes, after which the solution of $KC_8$ was added to the solution containing the Ni complex. The reaction was left at -78 °C for 30 minutes, while the color of the solution turned dark green. The mixture was filtered through a celite plug and used for EPR analysis. For **18**, the reaction was run in a mixture of $Et_2O$ and THF.

[(dtbpy)Ni(μ-Cl)]$_2$ **1**

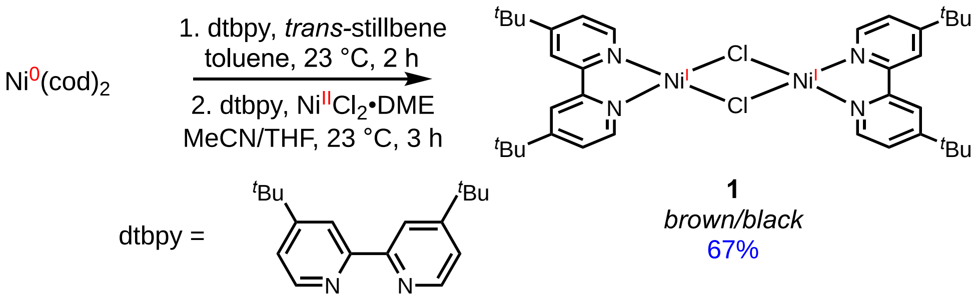


**1** was synthesized following a modified literature procedure.[28] In a nitrogen-filled glovebox, to a 20 mL scintillation vial was added Ni(cod)$_2$ (302.3 mg, 1.01 mmol, 1 equiv.), dtbpy (295.0 mg, 1.01 mmol, 1 equiv.), and toluene (5 mL, 0.2M). The reaction was allowed to stir at room temperature for 1 hour, during which the color of the solution turned dark purple. *Trans*-stillbene (198.1 mg, 1.01 mmol, 1 equiv.) was added to the solution and stirred an additional 30 minutes.

Solvent was removed under reduced pressure to obtain a dark purple crude residue, which was then triturated with toluene several times (4-5 x 5 mL) until a dark green color persisted upon solvation (indicating generation of (dtbpy)Ni(*trans*-stillbene)). Separately, $NiCl_2$•DME (241.5 mg, 1.01 mmol, 1 equiv.), dtbpy (295 mg, 1.01 mmol, 1 equiv.), and THF (5 mL, 0.2 M) were added to a 20 mL vial and stirred at room temperature until the color of solution becomes light green (~5-10 min). MeCN (5 mL, 0.2 M) was added to this *in situ* generated (dtbpy)$NiCl_2$ solution which was then added to (dtbpy)Ni(*trans*-stillbene) and stirred at room temperature for 3 hours.

The brown/black reaction mixture was then filtered through an alumina plug, and the collected filtrate concentrated under reduced pressure. The crude residue was washed with toluene (3 x 5

mL) and THF (3 x 5 mL) then dried to obtain the desired product as a dark brown/black powdery solid (535 mg, 67% yield). $^1$H NMR data agrees with reported literature.[28]

(dtbpy)Ni(Dipp*)Cl **19**

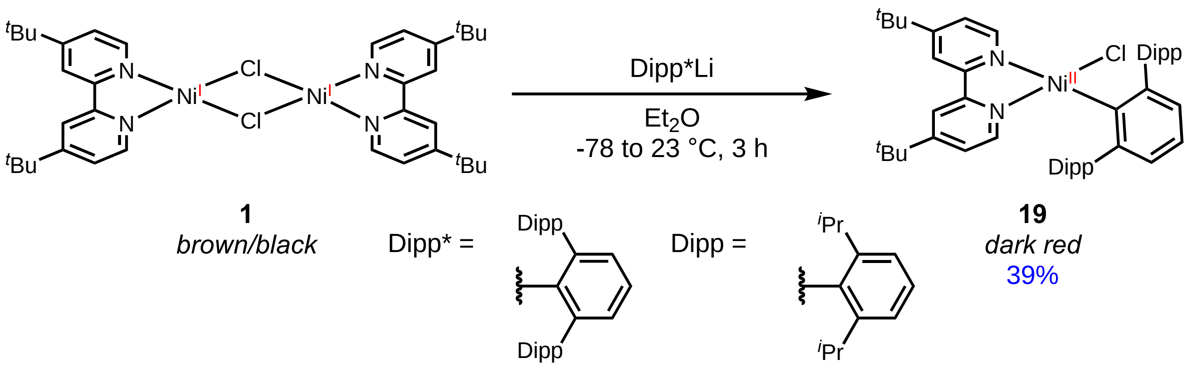


In a nitrogen-filled glovebox, to 2 separate 20 mL scintillation vials were added **1** (435.9 mg, 0.6 mmol, 1 equiv.) and Dipp*Li (243.2 mg, 0.6 mmol, 1 equiv.), and each dissolved in $Et_2O$ (4 mL each, 0.08 M). Both vials were then placed in a coldwell cooled to -78 °C for 15 minutes, after which the solution of Dipp*Li was added dropwise to the solution of **1**. The reaction mixture turned dark red immediately and was stirred at room temperature for 3 hours. The solution was then filtered through an alumina plug and solvent was removed under reduced pressure. The dark red residue was washed with cold pentane (3 x 3 mL) to remove protonated terphenyl and other impurities. ***Note:*** *The product is slightly soluble in pentane.*

The remaining red solids were extracted with $Et_2O$ and filtered through an alumina plug into a new oven-dried 20 mL vial. Upon solvent removal, the solids were triturated with pentane (3 x 5 mL) to afford **19** as a dark red crystalline solid (178 mg, 39% yield). Dark red crystals suitable for XRD were obtained from a concentrated $Et_2O$ solution stored in a -35 °C freezer over 3 days.

**$^1$H NMR (400 MHz, chloroform-*d*):** δ 7.58 (s, 1H), 7.50 (t, *J* = 7.5 Hz, 1H), 7.38 (t, *J* = 7.7 Hz, 2H), 7.31 (m, 2H), 7.24 – 7.16 (m, 7H), 7.05 (s, 1H), 6.90 (d, *J* = 7.1 Hz, 1H), 2.78 (sept., *J* = 6.8 Hz, 4H), 1.38 (s, 18H), 1.19 (d, *J* = 6.8 Hz, 12H), 1.10 (d, *J* = 6.8 Hz, 12H).

**$^{13}$C NMR (101 MHz, chloroform-*d*):** δ 162.3, 146.8, 143.1, 140.4, 139.3, 130.8, 127.85, 127.82, 127.76, 127.02, 122.5, 35.3, 30.70, 30.43, 30.27, 25.8, 24.29, 24.13, 22.6.

**UV-Vis (λ(ε), THF, 23 °C):** 298 nm (22670 $M^{-1}cm^{-1}$), 500 nm (4506 $M^{-1}cm^{-1}$).

**HRMS (ESI-TOF):** calculated for $[C_{48}H_{61}N_2ClNi + K]^+$: 797.3508, found: 797.3747.

(dtbpy)Ni(Dipp*) **21**

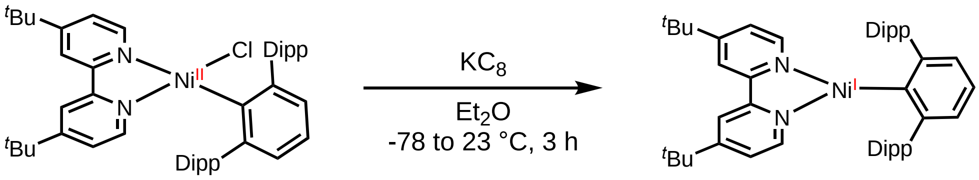


**19**
*dark red*

**21**
*dark green/black*

**21** was synthesized following the same procedure as (dtbpy)Ni(Dipp) **17**. After filtering through a celite plug, the filtrate was concentrated then redissolved in pentane. Dark green/black crystals suitable for XRD were obtained from a concentrated pentane solution stored in a -35 °C freezer over several days. No isolated yield obtained as the intermediate received upon reduction is the assumed four-coordinate, chloride-ligated Ni(I) (observable by EPR) and 3-coordinate Ni(I) only obtained upon crystallization.

**UV-Vis (λ, THF, 23 °C):** 300 nm, 418 nm, 735 nm.

[(dtbpy)Ni(η-3 Dipp*)][$BAr^F_{24}$] **22**

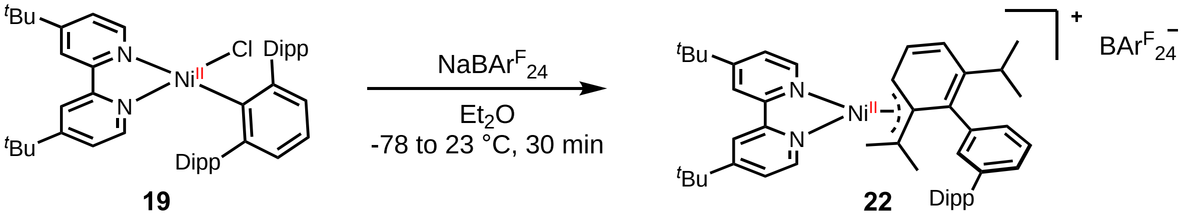


**19**
*dark red*

**22**
*dark orange*
37%

**22** was synthesized following the same reported procedure as [(dtbpy)Ni(Dipp*)][$BAr^F_{24}$] **16**, instead running the reaction in $Et_2O$. Upon completion, the reaction mixture was filtered through an alumina plug and solvent removed under reduced pressure. The crude residue was washed with pentane (3 x 5 mL) then extracted with toluene (3 x 3 mL) and filtered through a celite plug into a new vial. **22** was isolated as a foamy dark orange solid (96.1 mg, 37% yield) after removing solvent under reduced pressure. Dark orange crystals suitable for XRD were obtained from a concentrated $CH_2Cl_2$ solution layered with pentane stored in a -35 °C freezer overnight.

**$^1$H NMR (600 MHz, chloroform-*d*):** Diastereomeric ratio (major:minor): 1.4:1. Major diastereomer: δ 8.41 (d, *J* = 5.6 Hz, 1H), 8.17 (d, *J* = 8.7 Hz, 1H), 7.82 (s, 1H), 7.74 (s, 1H), 7.69 (bs, 8H), 7.61 (t, *J* = 7.2 Hz, 1H), 7.53 (t, *J* = 7.8 Hz, 1H), 7.49 (bs, 4H), 7.28 (d, *J* = 7.8 Hz, 1H), 7.22 (m, 7H), 7.17 (d, *J* = 7.3 Hz, 1H), 7.07 (d, *J* = 7.0 Hz, 2H), 6.46 (d, *J* = 7.0 Hz, 1H), 2.96 (sept., *J* = 6.8 Hz, 1H), 2.64 (sept, *J* = 6.8 Hz, 1H), 2.49 (sept, *J* = 6.8 Hz, 1H), 1.34 (s, 9H), 1.26 (s, 9H), 1.19 – 1.16 (m, 7.5H), 1.11 – 1.08 (m, 7.5H), 1.06 – 1.02 (m, 12H), 0.52 (d, *J* = 13.6 Hz, 6H), 0.34 (s, 3H). Minor diastereomer: δ 8.48 (d, *J* = 5.6 Hz, 1H), 8.19 (d, *J* = 8.7 Hz, 1H), 7.92 (s, 1H), 7.82 (s, 1H), 7.63 (d, *J* = 7.8 Hz, 1H), 7.44 (d, *J* = 7.8 Hz, 1H), 7.40 (t, *J* = 7.7 Hz, 1H), 7.36 (t, *J* = 7.7 Hz, 1H), 7.29 (d, *J* = 7.8 Hz, 1H), 7.12 (d, *J* = 5.6 Hz, 1H), 7.03 (s, 1H), 6.57 (d, *J* = 7.0 Hz, 1H), 3.02 (sept, *J* = 6.8 Hz, 1H), 2.79 (sept, *J* = 6.8 Hz, 1H), 2.58 (sept, *J* = 6.8 Hz, 1H),

1.37 (s, 9H), 1.27 (s, 9H), 1.14 (d, $J$ = 6.9 Hz, 3H), 1.05 (d, $J$ = 6.9 Hz, 3H), 0.49 (sept, $J$ = 6.7 Hz, 3H), 0.43 (s, 3H). Compound decomposes when attempting to get $^{13}$C NMR.

**$^{19}$F NMR (470 MHz, chloroform-*d*):** δ -61.78.

**UV-Vis (λ(ε), THF, 23 °C):** 295 nm (20908 $M^{-1}cm^{-1}$), 420 nm (289 $M^{-1}cm^{-1}$).

**HRMS (ESI-TOF)**: calculated for $[C_{80}H_{73}BF_{24}N_2Ni - C_{32}H_{12}BF_{24} + H]^+$: 724.4266, found: 724.4325.

(dtbpy)Ni(η-3 Dipp*) **23**

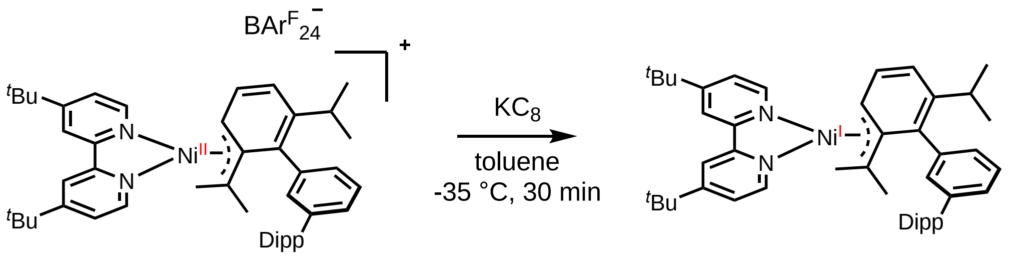


**22** *dark orange* **23** *light red/purple* observed spectroscopically by EPR

**23** was synthesized following the same reported procedure as (dtbpy)Ni(Dipp)* **21**. The reaction mixture was filtered through a celite plug to give a light red/purple solution used for EPR analysis.

(dtbpy)Ni($CH_2$TMS)$_2$ **25**

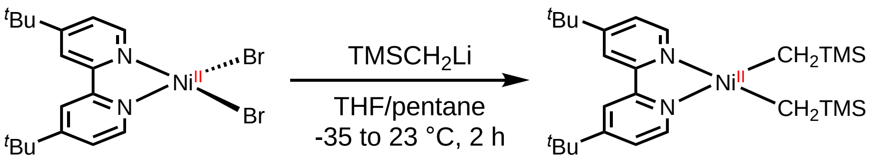


**25** *dark green* 62%

In a nitrogen-filled glovebox, to 2 separate 20 mL scintillation vials, (dtbpy)NiBr$_2$ (97.2 mg, 0.20 mmol, 1 equiv.) was suspended in THF (8 mL, 0.03 M) and seperately neosilyl lithium (37.9 mg, 0.40 mmol, 2 equiv.) was dissolved in pentane (2 mL, 0.1 M). Both vials were placed in a -35 °C freezer for 30 minutes then the solution of neosilyl lithium was added to the solution containing the Ni complex. The reaction was allowed to sit at -35 °C for an additional hour before stirring for 1 hour at room temperature then solvent was removed under reduced pressure. The resulting crude solid was extracted with toluene (3 x 3 mL) and filtered through celite. The filtrate solvent was removed under reduced pressure and the resulting solid was triturated with pentane (3 x 3 mL) to afford **25** as a dark green powder (63 mg, 62% yield).

**$^1$H NMR (500 MHz, benzene-*d*$_6$):** δ 9.04 (d, $J$ = 6.1 Hz, 2H), 7.44 (d, $J$ = 1.7 Hz, 2H), 6.73 (dd, $J$ = 6.1, 1.7 Hz, 2H), 0.96 (s, 18H), 0.56 (s, 18H), 0.36 (s, 4H).

**$^{13}$C NMR (126 MHz, benzene-$d_6$):** δ 158.8, 154.4, 148.1, 123.0, 116.8, 34.9, 29.8, 4.1, 1.7.

**UV-Vis (λ(ε), THF, 23 °C):** 304 nm (13368 $M^{-1}cm^{-1}$), 400 nm (4026 $M^{-1}cm^{-1}$), 634 nm (4056 $M^{-1}cm^{-1}$).

$[(dtbpy)Ni(CH_2TMS)_2]^-[K(18\text{-crown-}6)]^+$ **26**

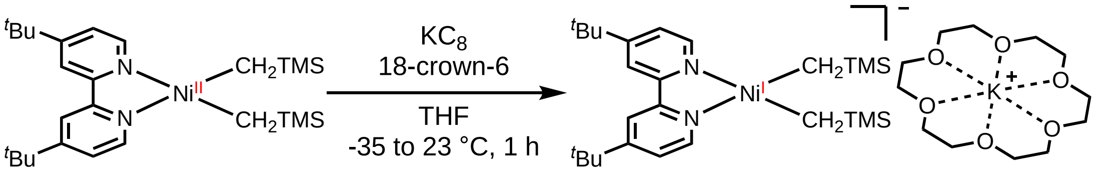


**25** *dark green* **26** *maroon* 76%

**26** was synthesized following a reported literature procedure.[15] In a nitrogen-filled glovebox, to a 20 mL scintillation vial were added **25** (26.2 mg, 0.05 mmol, 1 equiv.), 18-crown-6 (13.8 mg, 0.05 mmol, 1 equiv.), and THF (5 mL, 0.01M). $KC_8$ (10.6 mg, 0.08 mmol, 1.5 equiv.) and THF (5 mL, 0.01M) were added to a separate 20 mL vial, then both vials were allowed to cool in a -35 °C freezer for 15 minutes. The $KC_8$ suspension was added to the Ni-containing solution and the reaction was allowed to stir for 2 hours, gradually turning a dark red color over time. After filtering through celite the solvent was removed under reduced pressure and the resulting solids were washed with pentane (3 x 3 mL) to afford **26** as dark maroon oily solids (32 mg, 76% yield). Dark red crystals suitable for XRD were obtained from a concentrated solution of $Et_2O$ stored in a -35 °C freezer over several days. This compound shows no $^1$H or $^{13}$C NMR spectra.

**UV-Vis (λ, THF, 23 °C):** 250 nm, 307 nm, 378 nm, 540 nm, 636 nm.

$(phen)NiBr_2$ (phen = 1,10-phenanthroline) **27**

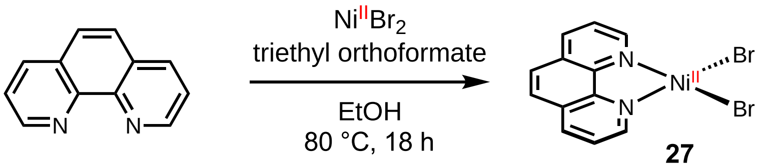


**27** *seafoam green* 66%

Outside of a nitrogen-filled glovebox, a flame-dried 3-neck flask fitted with a reflux condenser was charged with anhydrous $NiBr_2$ (5.46 g, 24.97 mmol, 3 equiv.). The flask was evacuated and backfilled with nitrogen three times then anhydrous EtOH (60 mL, 0.13M) and triethyl orthoformate (5.54 mL, 33.29 mmol, 4 equiv.) were added by syringe. The mixture was heated to reflux and stirred overnight, or until obtaining a homogeneous green solution (indicating generation of $NiBr_2$•4EtOH).

After cooling to room temperature, phen (1.50 g, 8.32 mmol, 1 equiv.) was added under a positive pressure of nitrogen and the mixture was stirred for an additional hour. The green precipitate was collected by vacuum filtration and washed with EtOH (3 x 30 mL) and hexanes (3 x 10 mL) to

afford **27** as a pale green powder (2.18 g, 66% yield). ***Note:*** *Consistency issues were observed when synthesizing this complex using different procedures. This method of preparation reliably afforded the desired product and products resulting from reduction between different batches of the prepared complex.*

[(phen)NiBr$_2$]$^-$[K(dibenzo-18-crown-6)]$^+$ **28**

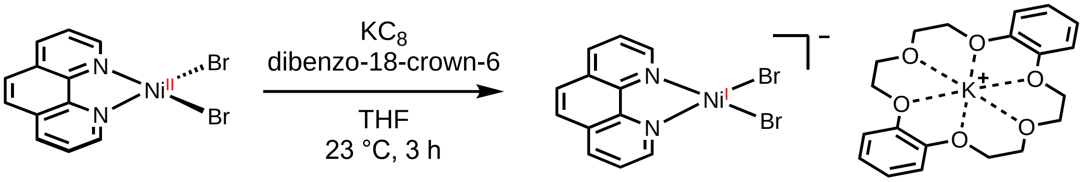


**27**
*seafoam green*

**28**
*dark violet*
77%

**28** was synthesized following the same reported procedure reported as [K(18-crown-6)][(dtbpy)Ni(CH$_2$TMS)$_2$] **26**, instead using THF (or DMF) as solvent and dibenzo-18-crown-6 (1 equiv.) as the crown chelator. After filtration, solvent was removed under reduced pressure and the resulting solids were washed with pentane (3 x 3 mL) to afford **28** as dark purple solids (71 mg, 77% yield). This complex shows no $^1$H or $^{13}$C NMR spectra.

**UV-Vis (λ, THF, 23 °C):** 223 nm, 265 nm, 362 nm, 554 nm, 609 nm.

(phen)Ni(CH$_2$TMS)$_2$ **29**

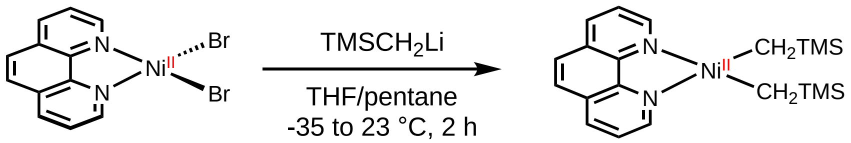


**27**
*seafoam green*

**29**
*navy blue*
47%

**29** was synthesized following the same procedure as (dtbpy)Ni(CH$_2$TMS)$_2$ **25**, affording **29** as a dark blue powder (160.0 mg, 47% yield). $^1$H NMR data agrees with reported literature.[29]

[(phen)Ni($CH_2$TMS)$_2$]$_2^-$[K(18-crown-6)]$_2^+$ **30, 30’**

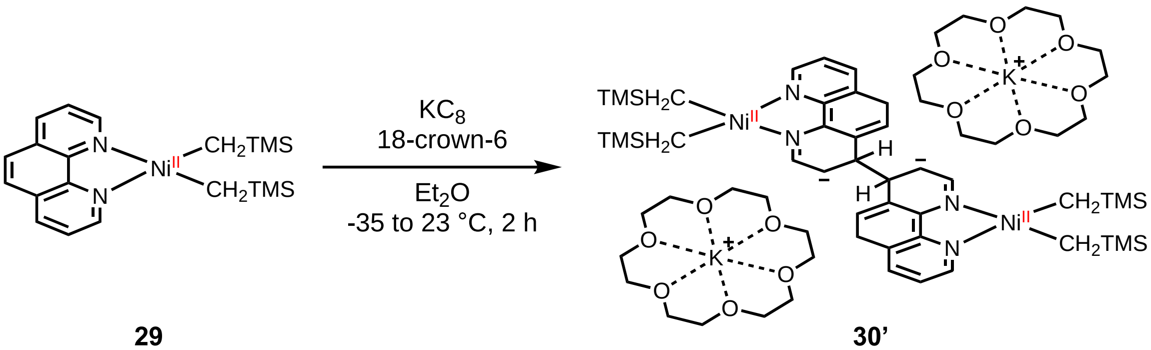


**29**
*navy blue*

**30’**
*dark teal*
98%

[K(18-crown-6)]$_2$[(phen)Ni($CH_2$TMS)$_2$]$_2$ **30’** was synthesized following the same reported procedure as [K(18-crown-6)][(dtbpy)Ni($CH_2$TMS)$_2$] **26**, affording **30’** as a dark teal microcrystalline solid (68.0 mg, 98% yield). Dark blue crystals suitable for XRD were obtained from a concentrated solution of $Et_2O$ layered with pentane stored in a -35 °C freezer overnight. This complex shows no $^1$H or $^{13}$C NMR spectra.

**UV-Vis (λ, THF, 23 °C):** 367 nm, 573 nm, 626 nm.

6-Mes-$^{dBn}$picolinamide **S7**

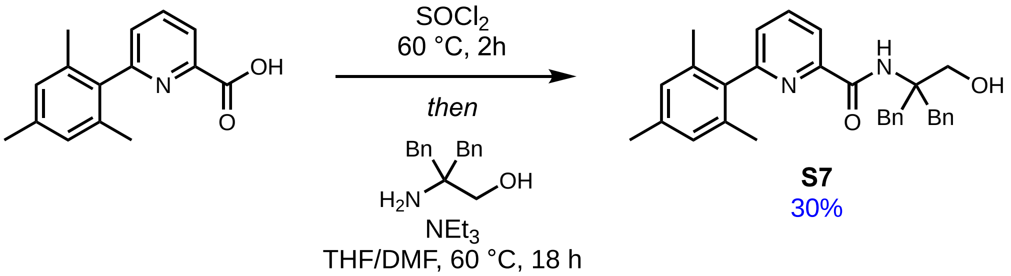


**S7**
30%

**S7** was synthesized following reported literature for an analogous compound.[30] To a flame dried 3-neck round bottom flask fitted with a reflux condenser was added 6-Mesitylpicolinic acid (770 mg, 3.19 mmol, 1 equiv.) followed by purging with $N_2$. Thionyl chloride (3.2 mL, 44 mmol, 14 equiv.) was added in one portion then the solution was refluxed for 2 hours. After cooling to room temperature, the solvent was evaporated under reduced pressure. The resulting residue was re-dissolved in anhydrous THF (5 mL, 0.45M) and DMF (1 mL, 2.25M) and added to a solution of 2,2-dibenzylglycinol (770 mg, 3.19 mmol, 1 equiv.) and triethylamine (1.33 mL, 9.57 mmol, 3 equiv.) dissolved in THF (5 mL, 0.45M). This mixture was brought to reflux and stirred for 12 hours. After cooling to room temperature, the reaction was neutralized with an aqueous saturated solution of $NaHCO_3$ (30 mL, 0.1M) and brine (30 mL, 0.1M). The organic phase was collected then the aqueous phase was extracted with EtOAc (3 x 30 mL). The combined organic extracts were dried over $MgSO_4$, filtered, and solvent removed under reduced pressure. The compound was purified by flash column chromatography (4:1, hexanes:EtOAc) to afford **S7** as a tan solid (442 mg, 30% yield).

**$^1$H NMR (400 MHz, chloroform-*d*):** δ 7.95 (dd, *J* = 7.8, 1.1 Hz, 1H), 7.82 (t, *J* = 7.8 Hz, 1H), 7.33 – 7.27 (m, 7H), 7.26 – 7.19 (m, 4H), 6.92 (s, 2H), 4.28 (s, 2H), 3.26 (d, *J* = 13.6 Hz, 2H), 3.02 (d, *J* = 13.6 Hz, 2H), 2.33 (s, 3H), 2.02 (s, 6H).

4,4-dibenzyl-2-(6-mesityl-2-pyridyl)-4,5-dihydrooxazole (6-Mes-$^{dBn}$pyrox) **S8**

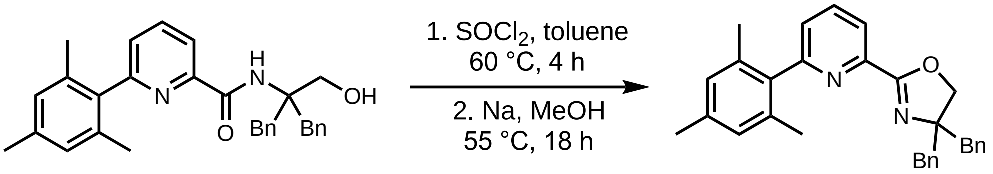


**S7**

**S8**
72%

**S8** was synthesized following a reported literature procedure for an analogous compound.[30b] To a flame dried round bottom flask was added **S7** (360 mg, 0.77 mmol, 1 equiv.) and anhydrous toluene (20 mL, 0.04M) under a $N_2$ atmosphere. The solution was heated to 60 °C then thionyl chloride (0.11 mL, 1.55 mmol, 2 equiv.) was dropwise added over 70 minutes with vigorous stirring. The reaction mixture was stirred for 4 hours at 60 °C after complete addition. After cooling to room temperature, the solvent is removed under reduced pressure. Separately, sodium (89 mg, 3.87 mmol, 5 equiv.) was added to anhydrous methanol (50 mL, 0.015M) and stirred until complete consumption of sodium to *in situ* generate fresh sodium methoxide. This methanol solution was then added to the crude residue containing the chlorinated 6-Mes-$^{dBn}$picolinamide and the reaction was stirred at 55 °C overnight under a $N_2$ atmosphere. After cooling to room temperature, the solvent is removed under reduced pressure. The resulting residue was re-dissolved in EtOAc (50 mL, 0.015M) and water (50 mL, 0.015M) then the organic phase was collected. The aqueous phase was extracted with EtOAc (3 x 30 mL) then the combined organic extracts were dried over $MgSO_4$, filtered, and solvent removed under reduced pressure. The compound was purified by flash column chromatography (4:1, hexanes:EtOAc) to afford **S8** as an off-white solid (250 mg, 72% yield).

**$^1$H NMR (500 MHz, chloroform-*d*):** δ 7.90 (d, *J* = 7.8 Hz, 1H), 7.77 (t, *J* = 7.8 Hz, 1H), 7.26 – 7.15 (m, 11H), 6.86 (s, 2H), 4.22 (s, 2H), 3.21 (d, *J* = 13.6 Hz, 2H), 2.97 (d, *J* = 13.6 Hz, 2H), 2.28 (s, 3H), 1.96 (s, 6H).

**$^{13}$C NMR (126 MHz, chloroform-*d*):** δ 162.4, 160.3, 146.9, 137.55, 137.46, 136.96, 136.64, 135.91, 130.9, 128.28, 128.15, 126.6, 121.8, 74.9, 73.3, 68.1, 45.9, 25.7, 21.2, 20.3.

**HRMS (ESI-TOF):** calculated for $[C_{31}H_{30}N_2O + H]^+$: 447.2431, found: 447.2577.

(6-Mes-$^{dBn}$pyrox)$NiCl_2$ **31**

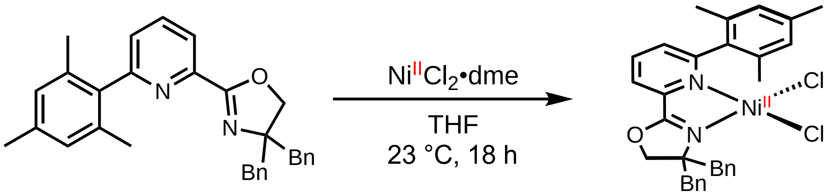


**S8**

**31**
*pale pink*
99%

**31** was synthesized following the same reported procedure as ($^{Cy}$bpy)$NiCl_2$ **11**, affording **31** as a pale pink powder (25 mg, 99% yield).

**$^1$H NMR (400 MHz, acetone-$d_6$):** δ 73.95 (s, 1H), 47.77 (s, 1H), 24.55 (bs, 2H), 21.96 (s, 2H), 20.51 (bs, 2H), 18.18 (s, 1H), 12.79 (s, 2H), 7.13 (t, $J$ = 12.8 Hz, 2H), 6.52 (s, 3H), 6.48 (d, $J$ = 12.8 Hz, 4H), 6.42 (s, 5H), 5.59 (bs, 4H), 2.97 (s, 1H). This compound shows no $^{13}$C NMR spectrum.

**UV-Vis (λ(ε), THF, 23 °C):** 266 nm (5898 $M^{-1}cm^{-1}$), 508 nm (70 $M^{-1}cm^{-1}$).

(6-Mes-$^{dBn}$pyrox)NiCl **32**

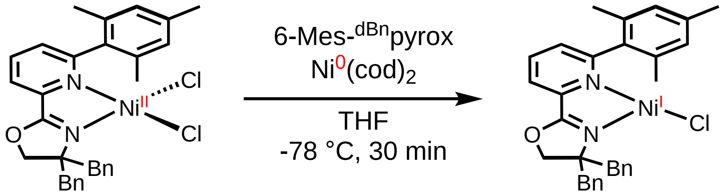


**31**
*pale pink*

**32**
*dark emerald green*
95%

**32** was synthesized following the same reported procedure as ($^{Mes}$dtbpy)NiBr **13**, affording **32** as a dark emerald green crystalline solid (23 mg, 95% yield). Dark green crystals suitable for XRD were obtained from a concentrated solution of THF layered with pentane stored in a -35 °C freezer over several days. This compound shows no $^1$H or $^{13}$C spectrum. ***Note:*** *(6-Mes-$^{dBn}$pyrox)NiCl* ***32*** *rapidly decomposes at room temperature.*

**UV-Vis (λ, THF, 23 °C):** 275 nm, 429 nm, 705 nm.

(S)-4-(*tert*-butyl)-2-(6-mesityl-2-pyridyl)-4,5-dihydrooxazole (6-Mes-$^{tBu}$pyrox) **S9**

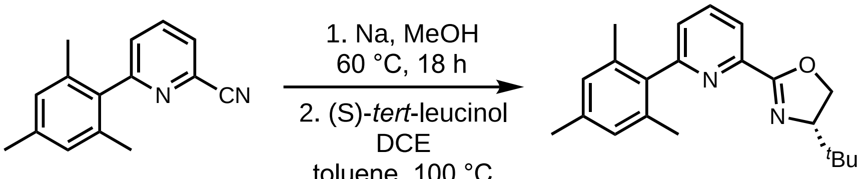


**S9**
46%

**S9** was synthesized following a reported literature procedure for an analogous compound.[5] Sodium (15.9 mg, 0.69 mmol, 1.1 equiv.) was added to anhydrous methanol (15 mL. 0.04M) in a flame dried flask under an inert atmosphere and stirred until complete consumption of sodium to *in situ* generate fresh sodium methoxide. 6-Mesityl-2-pyridinecarbonitrile (140 mg, 0.63 mmol, 1 equiv.) was added to this solution and stirred at 60 °C overnight. After cooling to room temperature, the solvent was removed under reduced pressure. The resulting residue was redissolved in EtOAc (30 mL) and water (20 mL). The organic phase was separated and washed with brine (2 x 20 mL), then dried over $MgSO_4$. After filtering and removing solvent under reduced pressure, *tert*-leucinol (160 mg, 0.63 mmol, 1 equiv.), anhydrous toluene (10 mL, 0.06M), and a catalytic amount of dichloroethane were added to the flask and this mixture was stirred overnight at 100 °C. After cooling to room temperature, the solvent was removed under reduced pressure. The compound was purified by flash column chromatography (4:1, hexanes:EtOAc) to afford **S9** as a colorless solid (93 mg, 46% yield).

**$^1$H NMR (400 MHz, chloroform-*d*):** δ 8.09 (d, *J* = 7.8 Hz, 1H), 7.81 (d, *J* = 7.8 Hz, 1H), 7.30 (d, *J* = 7.8, 1.1 Hz, 1H), 6.89 (s, 2H), 4.43 (dd, *J* = 10.2, 8.5 Hz, 1H), 4.30 (t, *J* = 8.5 Hz, 1H), 4.10 (dd, *J* = 10.2, 8.5 Hz, 1H), 2.30 (s, 3H), 2.01 (s, 6H), 0.98 (s, 9H).

(6-Mes-$^{tBu}$pyrox)NiCl **S10**

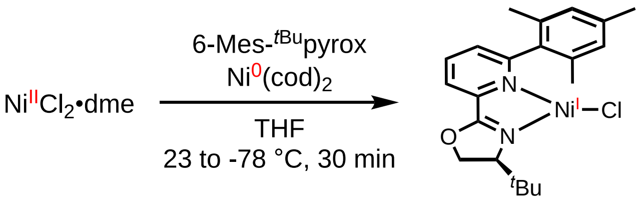


**S10**
*dark emerald green*
observed spectroscopically by EPR

**S10** was synthesized following the same reported procedure as ($^{Mes}$dtbpy)NiBr **13**, instead *in situ* generating (6-Mes-$^{tBu}$pyrox)$NiCl_2$. **S9** (30 mg, 0.09 mmol, 1 equiv.) and $NiCl_2$•dme (94 mg, 0.47 mmol, 5 equiv.) were stirred for 2 hours in THF (2 mL, 0.02M) at room temperature, then filtered through celite into a new vial. A separate solution of **S9** (1 equiv.) and $Ni(cod)_2$ (1 equiv.) in THF (2 mL, 0.02M) was added to this light pink solution and color immediately turned dark green. The solution was placed in a Coldwell cooled to -78 °C and allowed to sit for 30 min. The dark green solution was filtered and used for EPR analysis.

($(^{t\mathrm{Bu}}$pyrox)Ni(Dipp)Br **33**

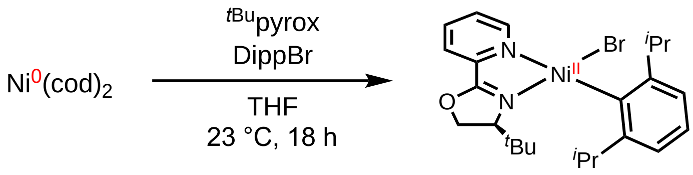


**33**
*dark red*
77%

**33** was synthesized following the same reported procedure as (dtbpy)Ni(Dipp)Br **15**, affording **33** as a dark red powder (95 mg, 77% yield). $^1$H NMR data agrees with reported literature.[15]

**HRMS (ESI-TOF):** calculated for $[C_{24}H_{33}BrN_2ONi + Na]^+$: 525.1027, found: 525.1379.

[($^{t\mathrm{Bu}}$pyrox)Ni(Dipp)][BAr$^F_{24}$] **34**

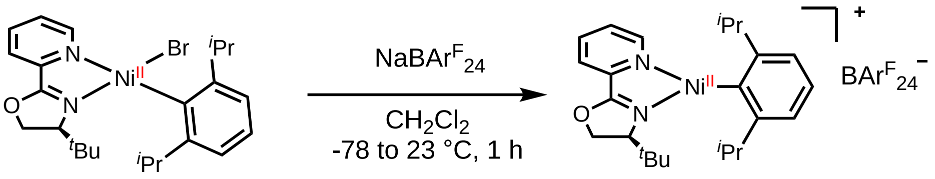


**33**
*dark red*

**34**
*orange red*
61%

**34** was synthesized following the same reported procedure as [(dtbpy)Ni(Dipp)][BAr$^F_{24}$] **16**, affording **34** as an orange red powder (62 mg, 61% yield). ***Note***: *If coordinating solvent (like THF) is not fully removed from starting material then the product requires extra pentane triturations to obtain a foamy solid from an oily residue. Residual coordinating solvent also broadens NMR shifts of desired product and turns the color more red.*

**$^1$H NMR (400 MHz, chloroform-*d*):** $\delta$ 8.38 (s, 8H), 7.67 (s, 4H), 6.85 (d, $J$ = 5.4 Hz, 1H), 6.78 (t, $J$ = 7.2 Hz, 1H), 6.69 (d, $J$ = 7.2 Hz, 1H), 6.64 (d, $J$ = 7.6 Hz, 2H), 6.56 (t, $J$ = 7.6 Hz, 1H), 6.34 (sept., $J$ = 6.8 Hz, 1H), 5.94 (t, $J$ = 6.9 Hz, 1H), 5.29 (sept., $J$ = 6.9 Hz, 1H), 3.93 (dd, $J$ = 9.5, 3.1 Hz, 1H), 3.66 (dd, $J$ = 8.9, 3.1 Hz, 1H), 3.41 (q, $J$ = 9.2 Hz, 1H), 1.54 (d, $J$ = 6.8 Hz, 3H), 1.34 (d, $J$ = 8.8 Hz, 3H), 1.19 (d, $J$ = 6.6 Hz, 3H), 0.83 (d, $J$ = 6.8 Hz, 3H), 0.79 (s, 9H). Compound decomposes when attempting to get $^{13}$C NMR.

**$^{19}$F NMR (470 MHz, chloroform-*d*):** $\delta$ -62.17.

**UV-Vis ($\lambda$($\varepsilon$), THF, 23 °C):** 280 nm (5927 $M^{-1}cm^{-1}$).

**HRMS (ESI-TOF):** calculated for $[C_{56}H_{45}N_2OBF_{24}Ni - C_{32}H_{12}BF_{24} + H]^+$: 424.2019, found: 424.2081.

($^{tBu}$pyrox)Ni(Dipp) **35**

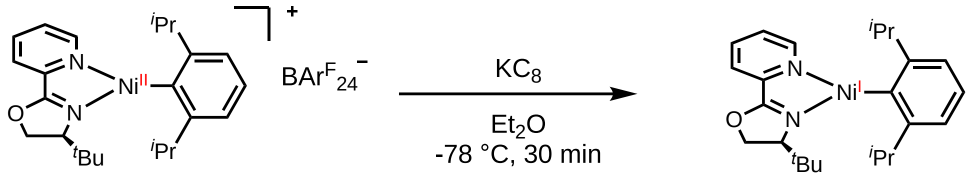


**34**
*orange red*

**35**
*dark green*
observed spectroscopically by EPR

**35** was synthesized following the same reported procedure as (dtbpy)Ni(Dipp)* **21**. The reaction mixture was filtered through a celite plug to give a dark green solution used for EPR analysis.

### 4. UV-Vis Spectra

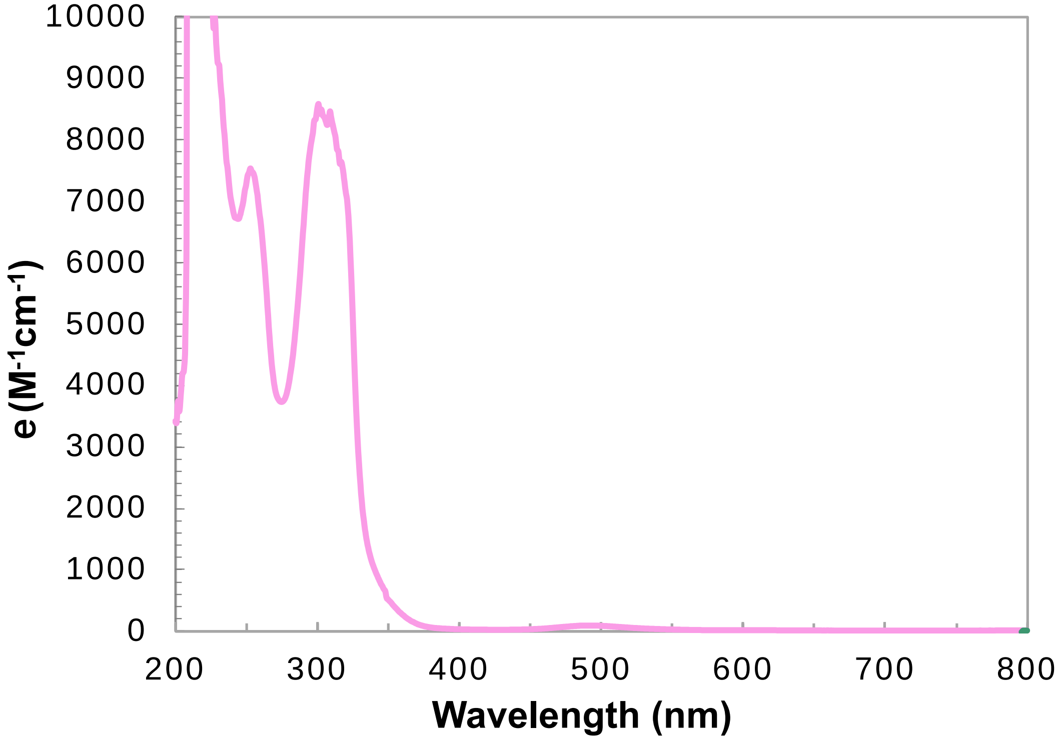


**Figure S1.** UV-Vis of ($^{Cy}$bpy)NiCl$_2$ (**11**) (THF, 23 °C, 0.44 mM).

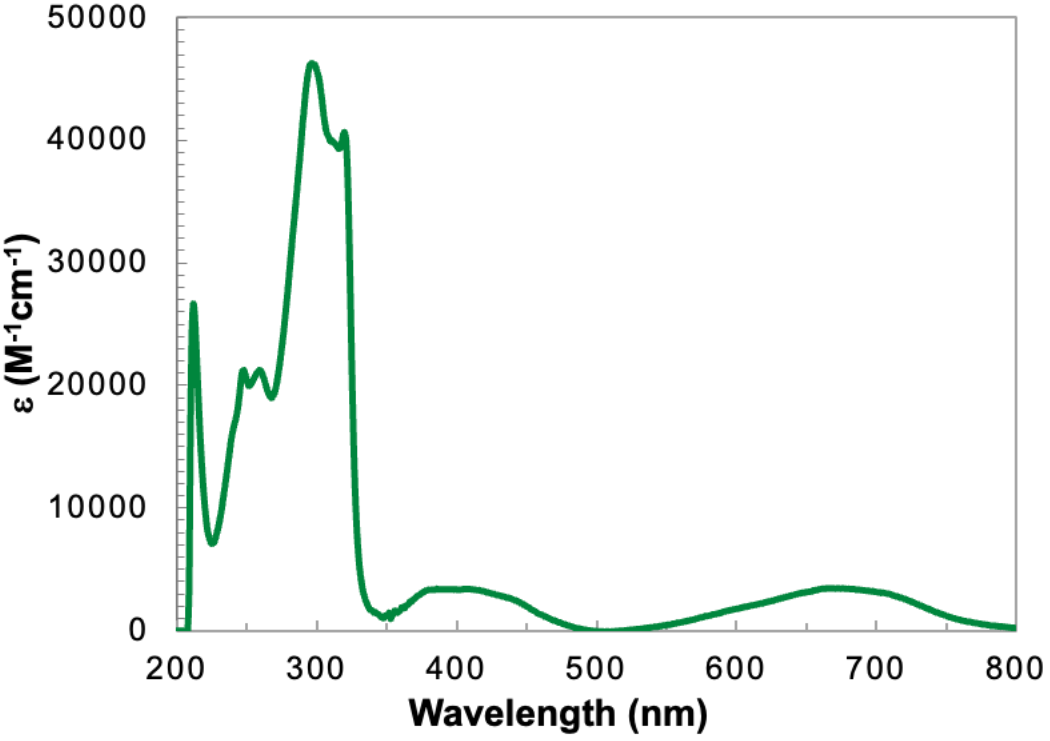


**Figure S2.** UV-Vis of ($^{Cy}$bpy)NiCl (**12**) (THF, 23 °C, 0.01 mM).

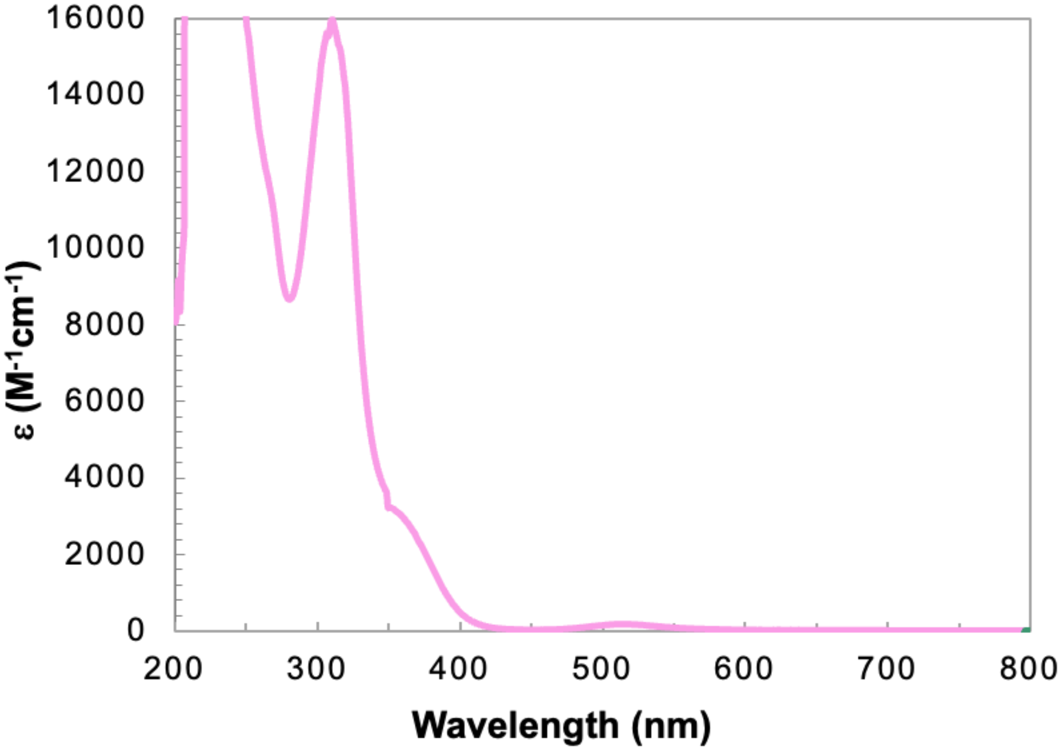


**Figure S3.** UV-Vis of ($^{Mes}$dtbpy)$NiBr_2$ (**S6**) (THF, 23 °C, 0.22 mM).

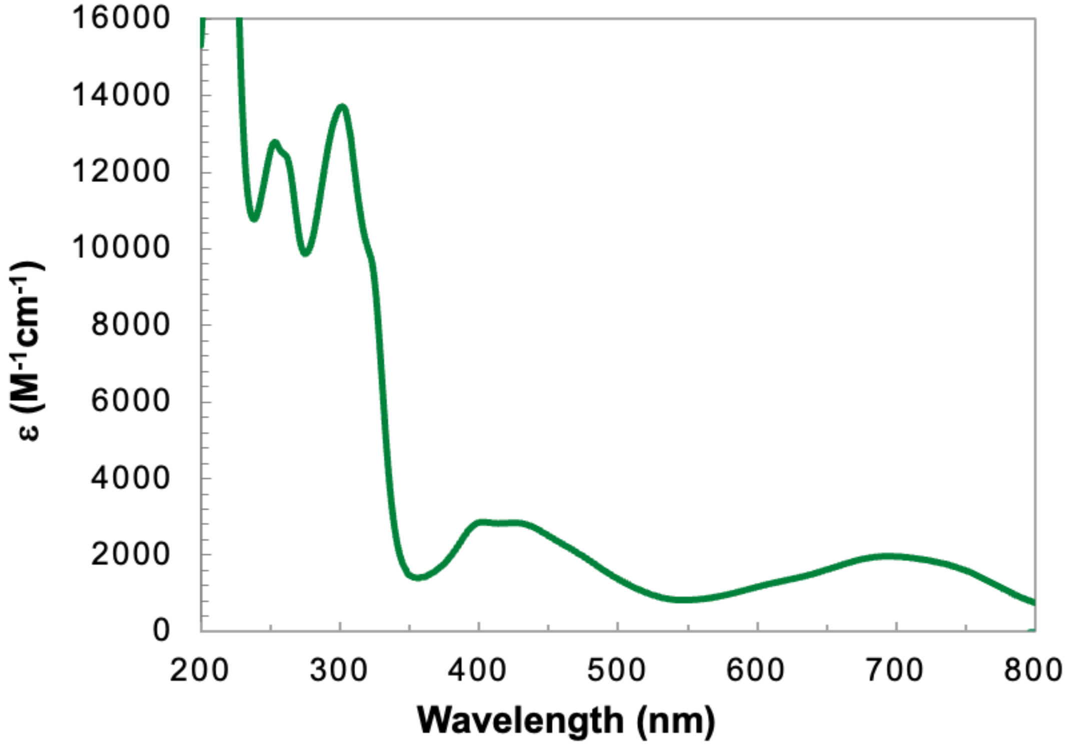


**Figure S4.** UV-Vis of ($^{Mes}$dtbpy)NiBr (**13**) (THF, 23 °C, 0.07 mM).

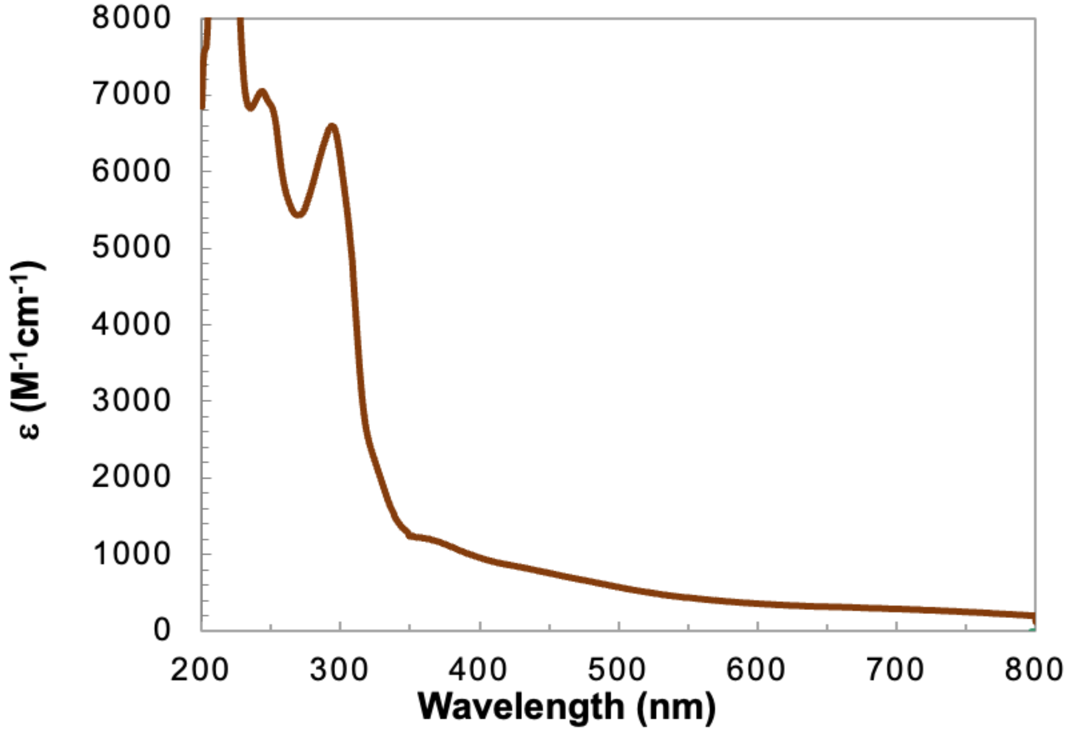


**Figure S5.** UV-Vis of ($^{Mes}$dtbpy)Ni($CH_2$TMS) (**14**) (THF, 23 °C, 0.12 mM).

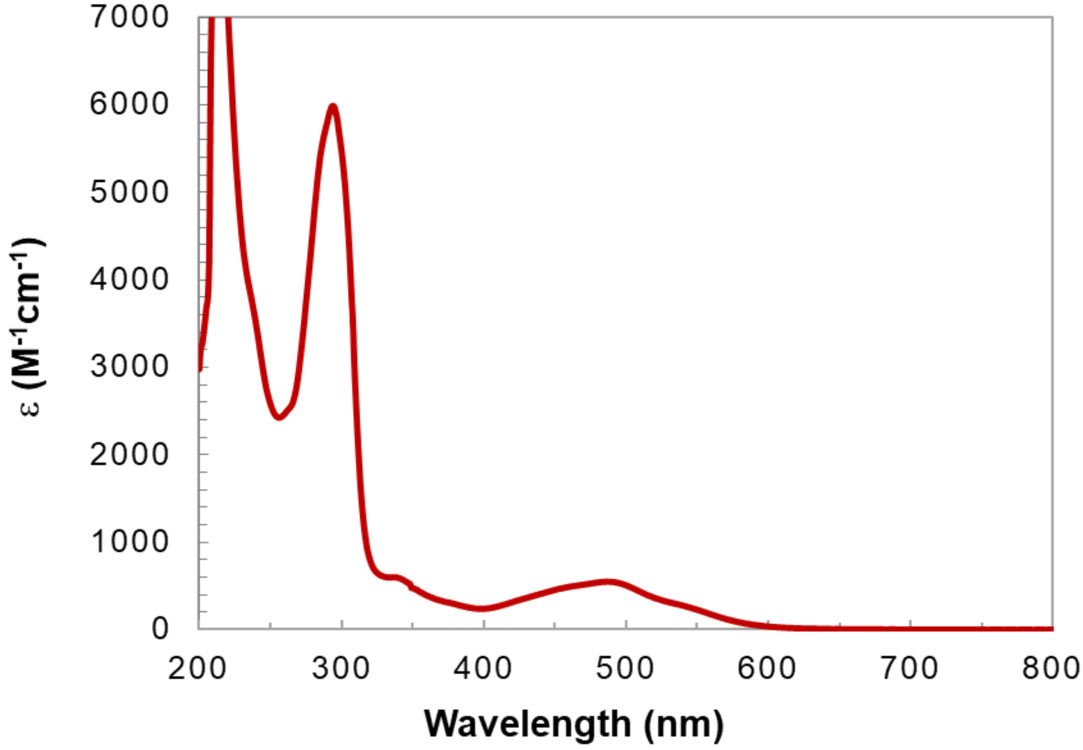


**Figure S6.** UV-Vis of (dtbpy)Ni(Dipp)Br (**15**) (THF, 23 °C, 0.11 mM).

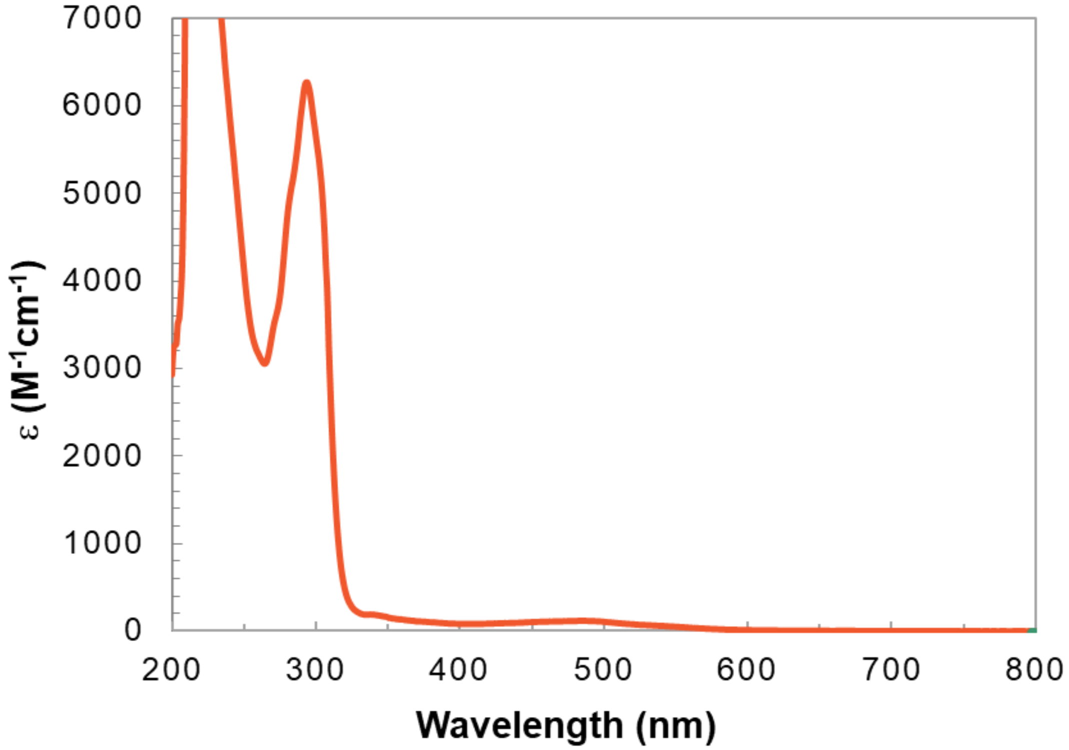


**Figure S7.** UV-Vis of [(dtbpy)Ni(Dipp)][$BAr^{F}_{24}$] (**16**) (THF, 23 °C, 0.12 mM).

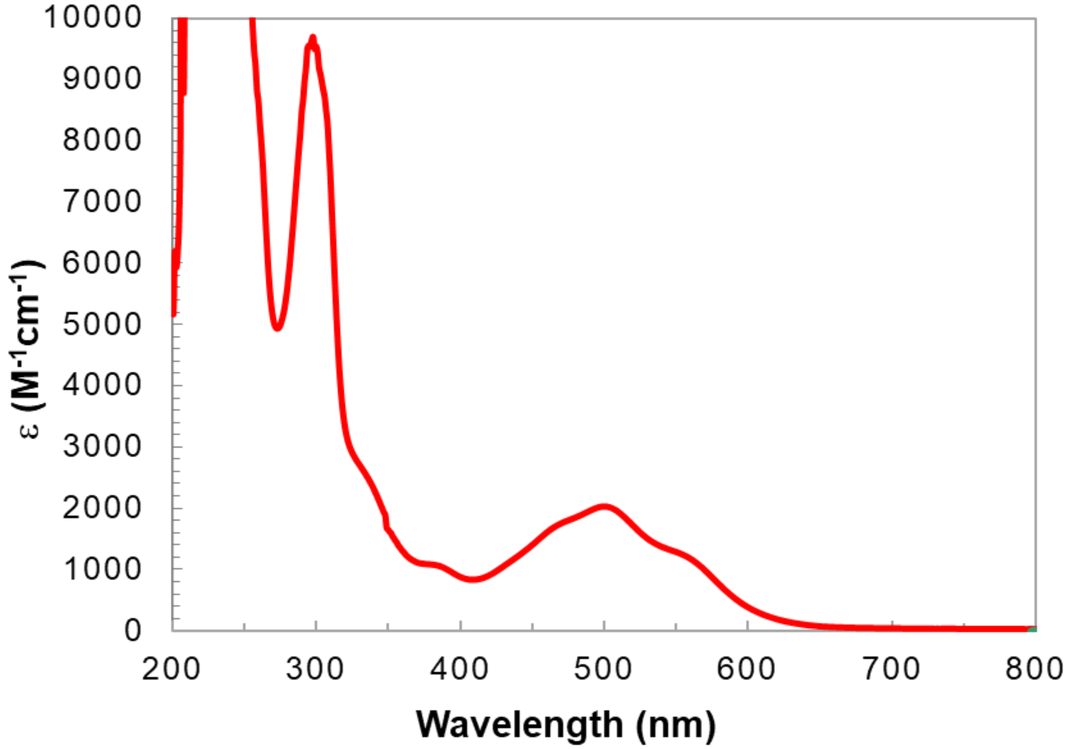


**Figure S8.** UV-Vis of (dtbpy)Ni(Dipp*)Cl (**19**) (THF, 23 °C, 0.08 mM).

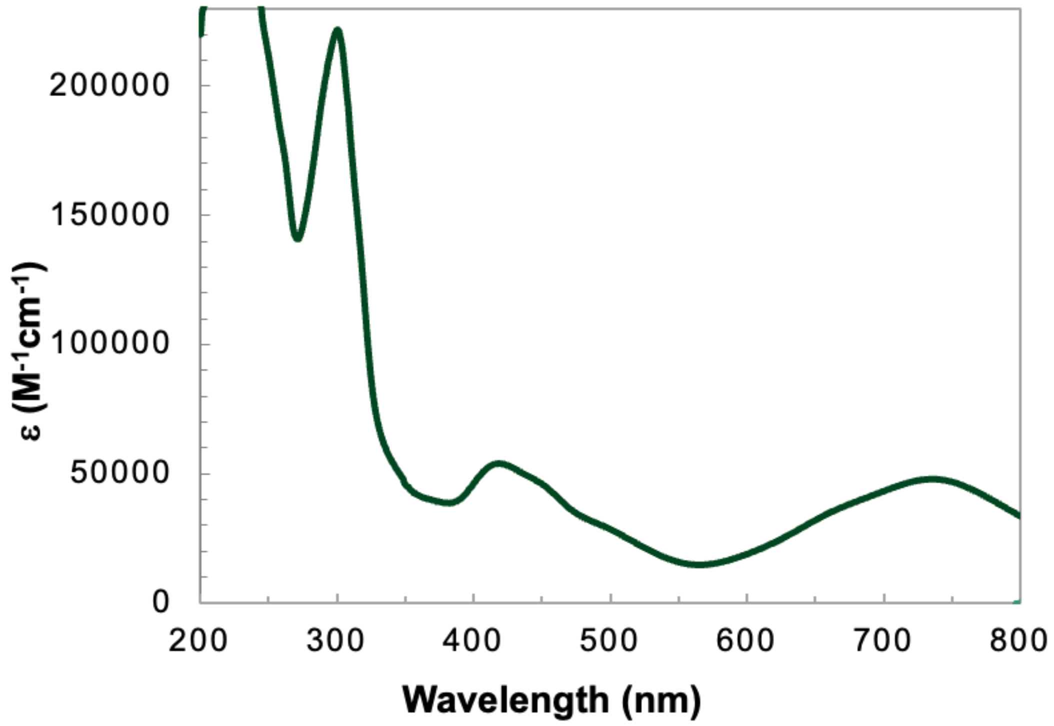


**Figure S9.** UV-Vis of (dtbpy)Ni(Dipp*) (**21**) (THF, 23 °C, 0.01 mM).

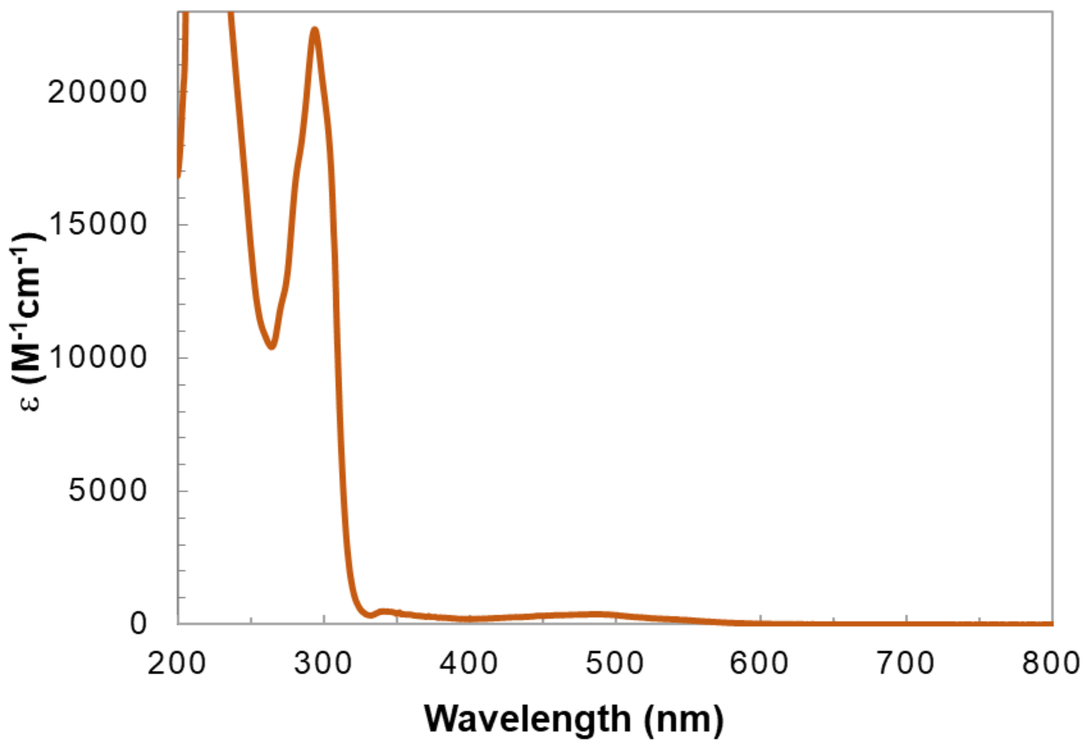

**Figure S10.** UV-Vis of [(dtbpy)Ni(η-3 Dipp*)][BAr$^{F}{}_{24}$] (**22**) (THF, 23 °C, 0.06 mM).

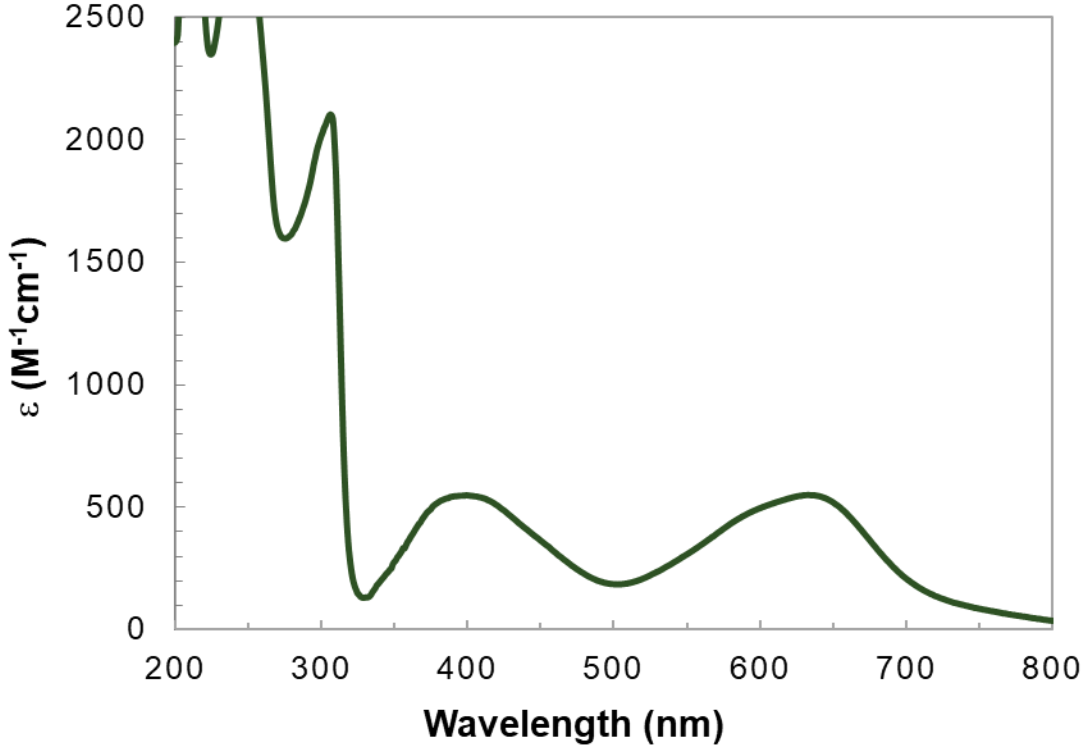

**Figure S11.** UV-Vis of (dtbpy)Ni($CH_2$TMS)$_2$ (**25**) (THF, 23 °C, 0.07 mM).

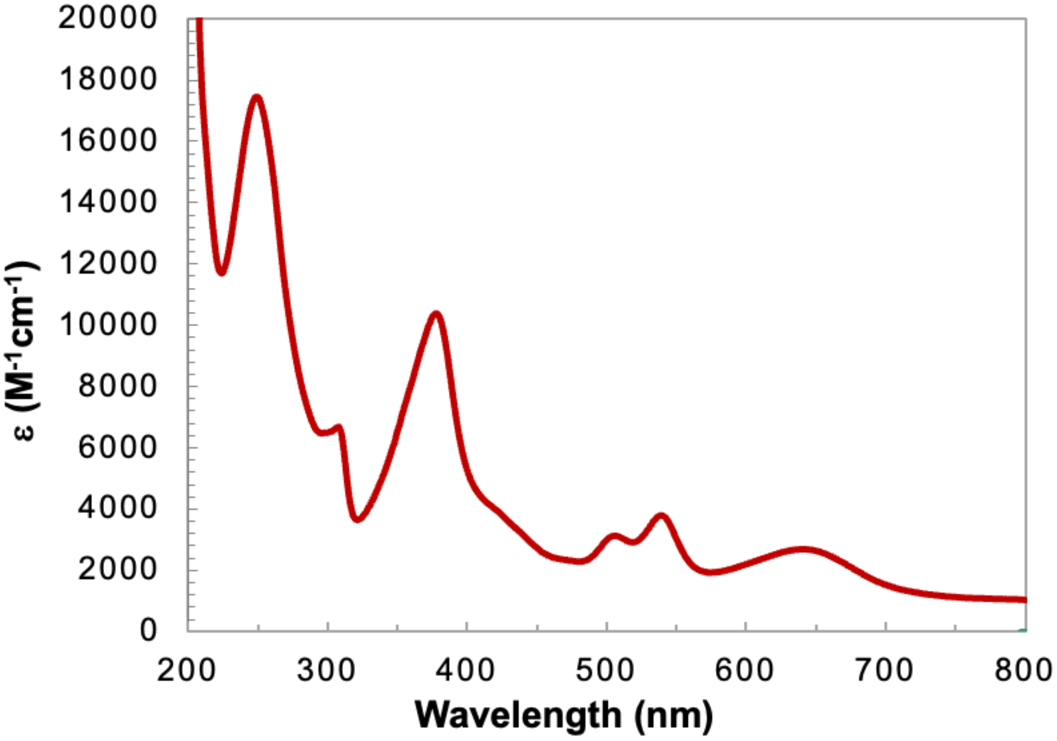


**Figure S12.** UV-Vis of $[(dtbpy)Ni(CH_2TMS)_2]^-[K(18\text{-}crown\text{-}6)]^+$ (**26**) (THF, 23 °C, 0.1 mM).

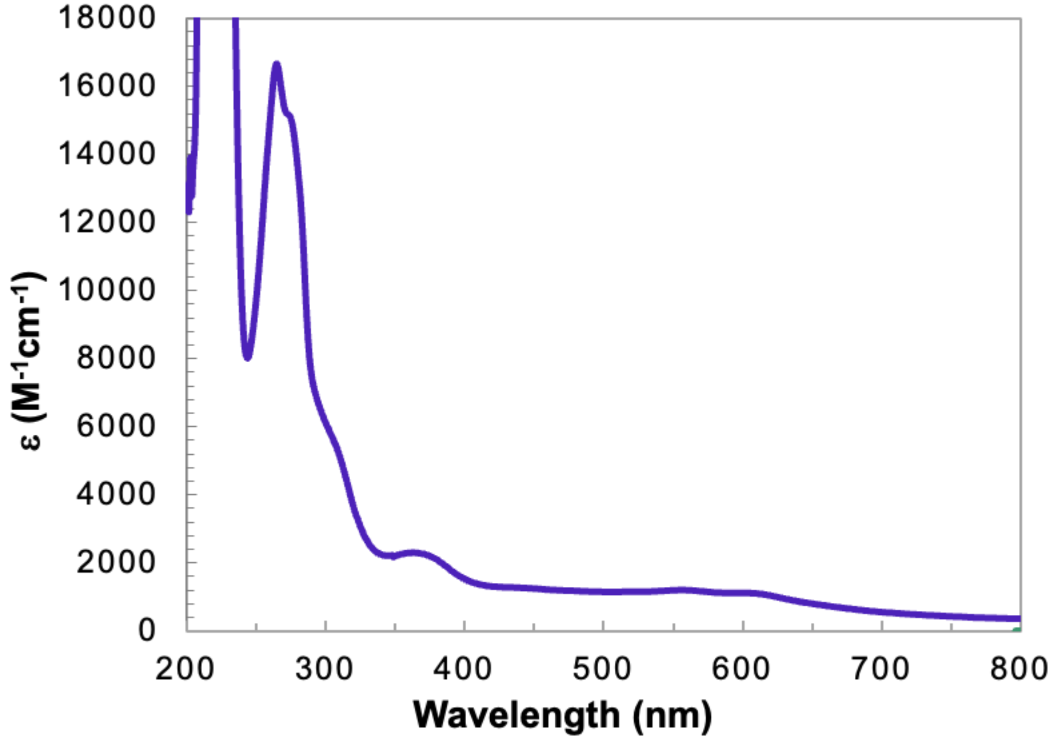


**Figure S13.** UV-Vis of $[(phen)NiBr_2]^-[K(dibenzo\text{-}18\text{-}crown\text{-}6)]^+$ (**28**) (THF, 23 °C, 0.13 mM).

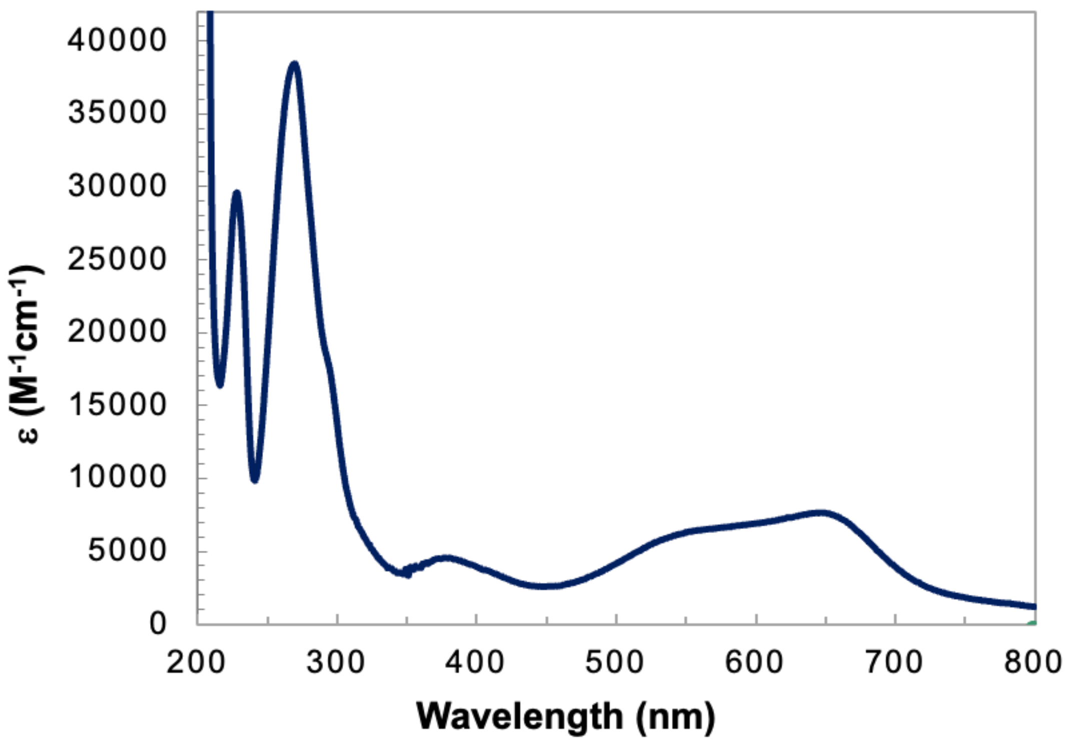


**Figure S14.** UV-Vis of [(phen)Ni($CH_2$TMS)$_2$]$_2^-$[K(18-crown-6)]$_2^+$ (**30'**) (THF, 23 °C, 0.01 mM).

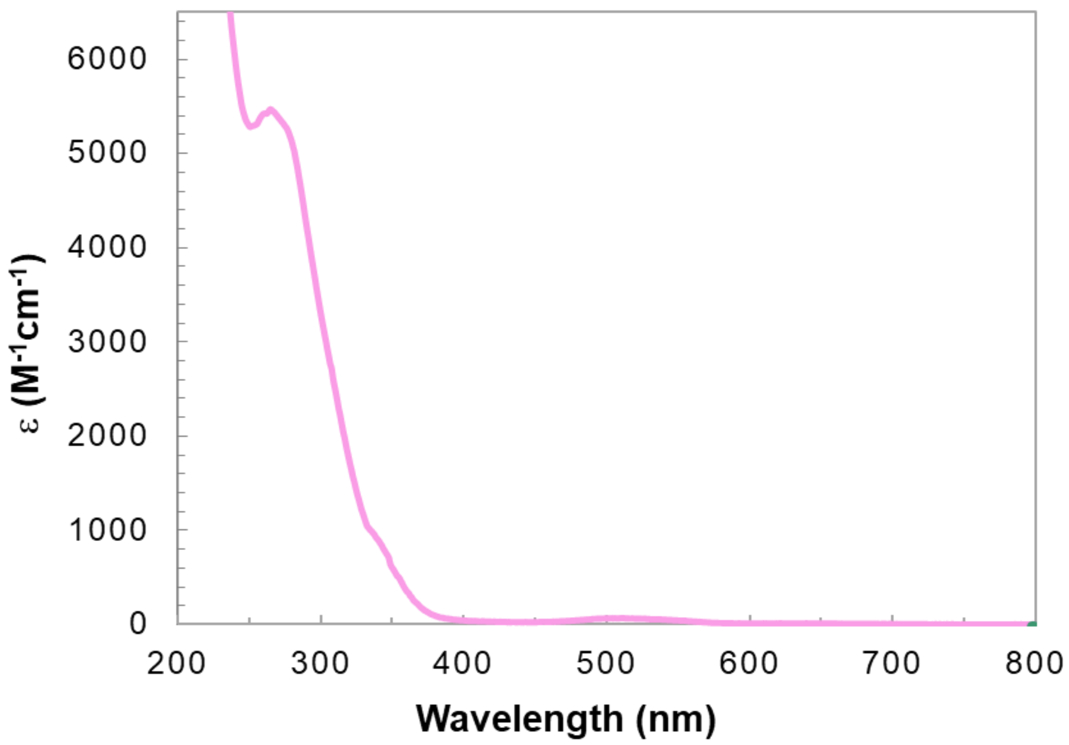


**Figure S15.** UV-Vis of (6-Mes-$^{dBn}$pyrox)NiCl$_2$ (**31**) (THF, 23 °C, 0.11 mM).

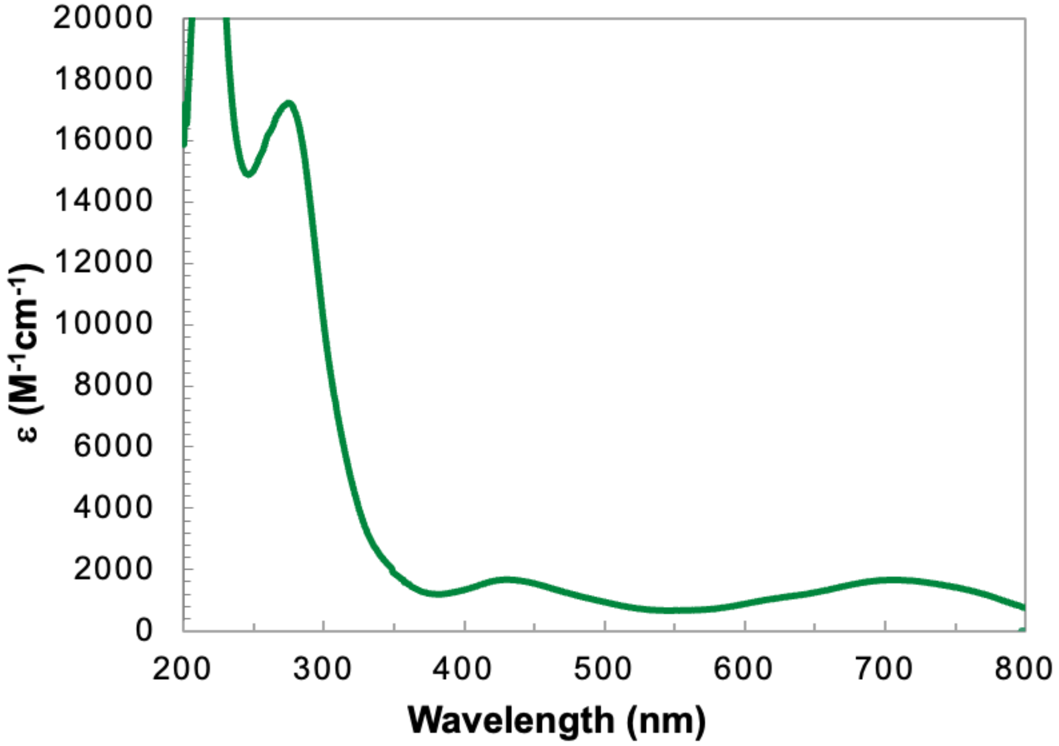


**Figure S16.** UV-Vis of ((6-Mes-$^{dBn}$pyrox)NiCl (**32**) (THF, 23 °C, 0.09 mM).

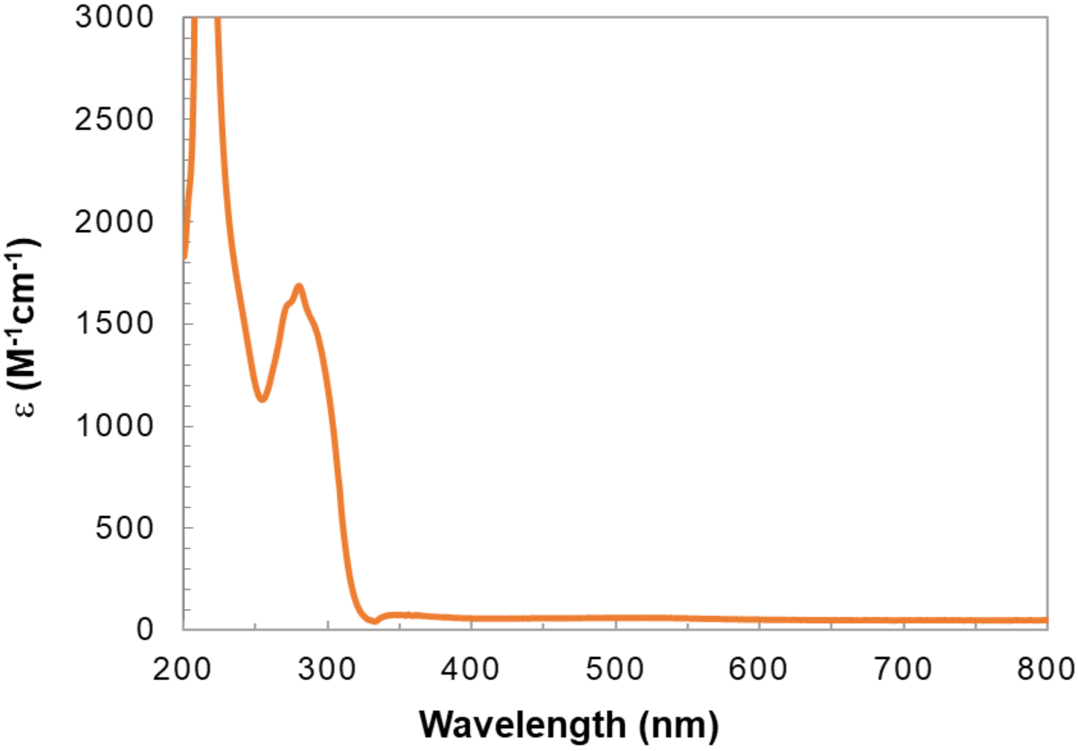


**Figure S17.** UV-Vis of [($^{tBu}$pyrox)Ni(Dipp)][$BAr^F_{24}$] (**34**) (THF, 23 °C, 0.04 mM).

## 5. NMR Spectra

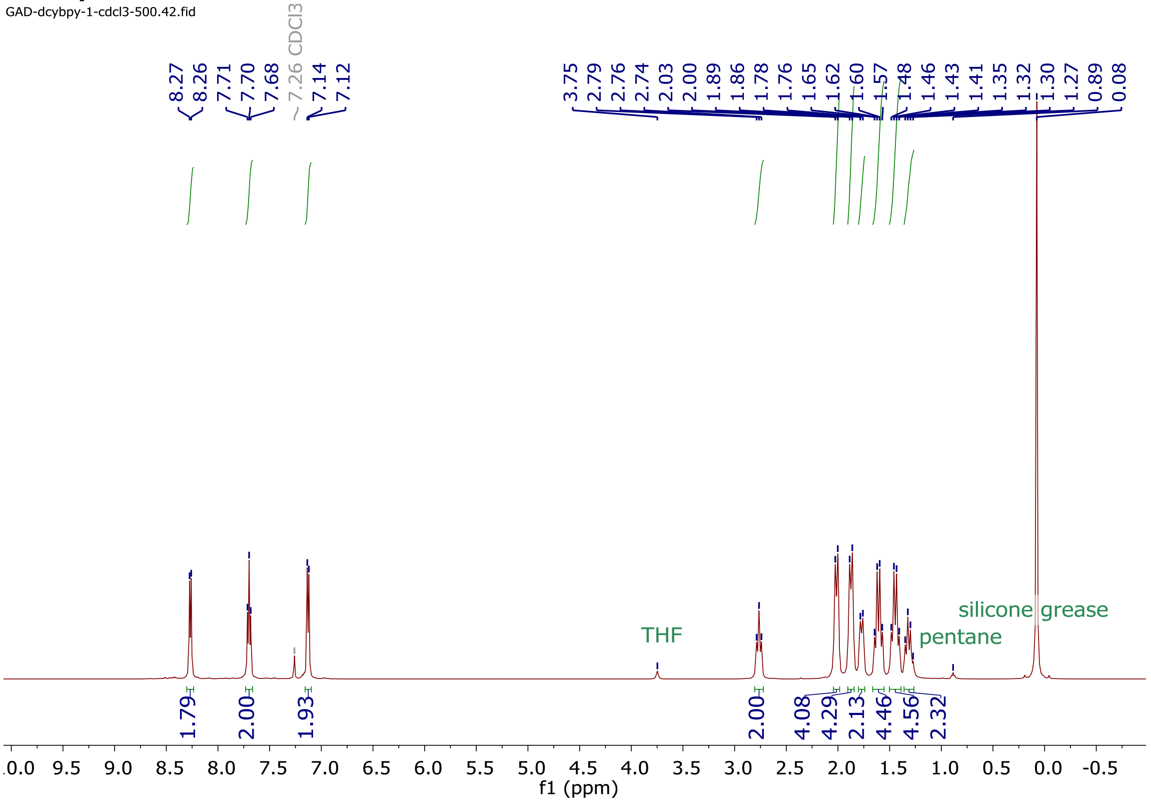


**Figure S18.** $^{1}$H NMR (500 MHz, chloroform-*d*, 23 °C) of $^{Cy}$bpy **S1**.

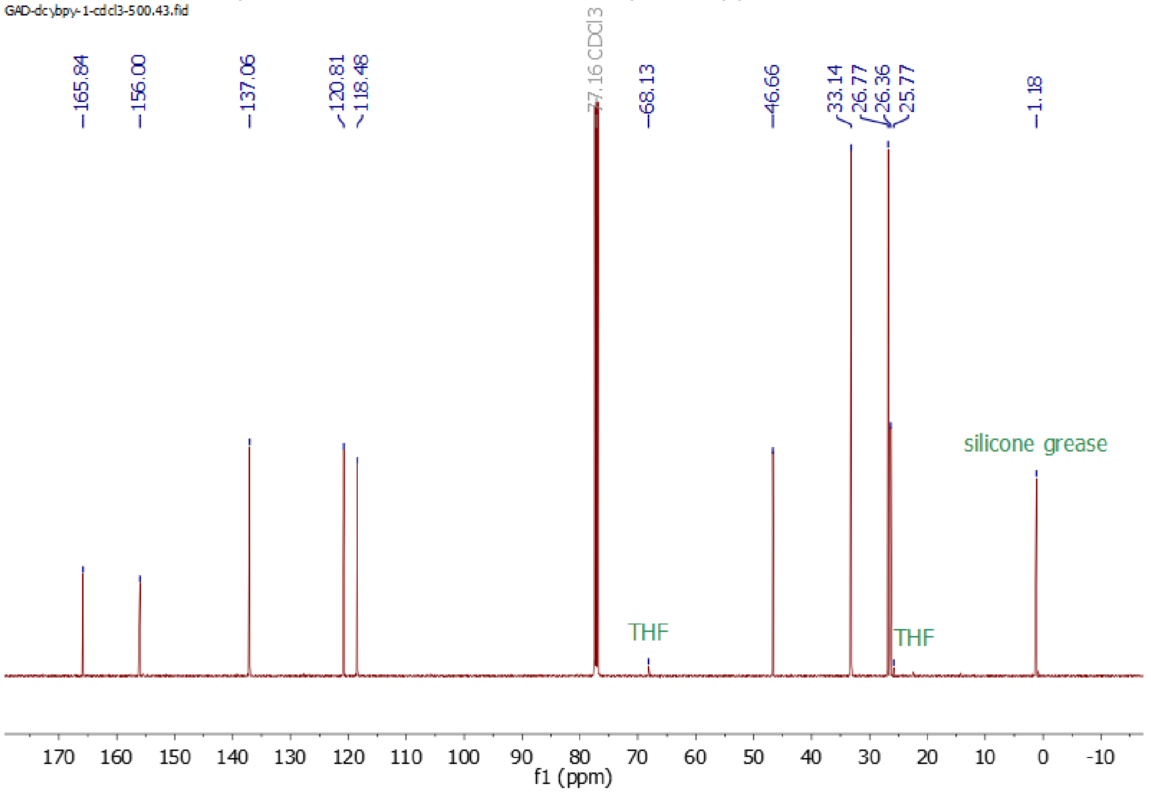


**Figure S19.** $^{13}$C NMR (126 MHz, chloroform-*d*, 23 °C) of $^{Cy}$bpy **S1**.

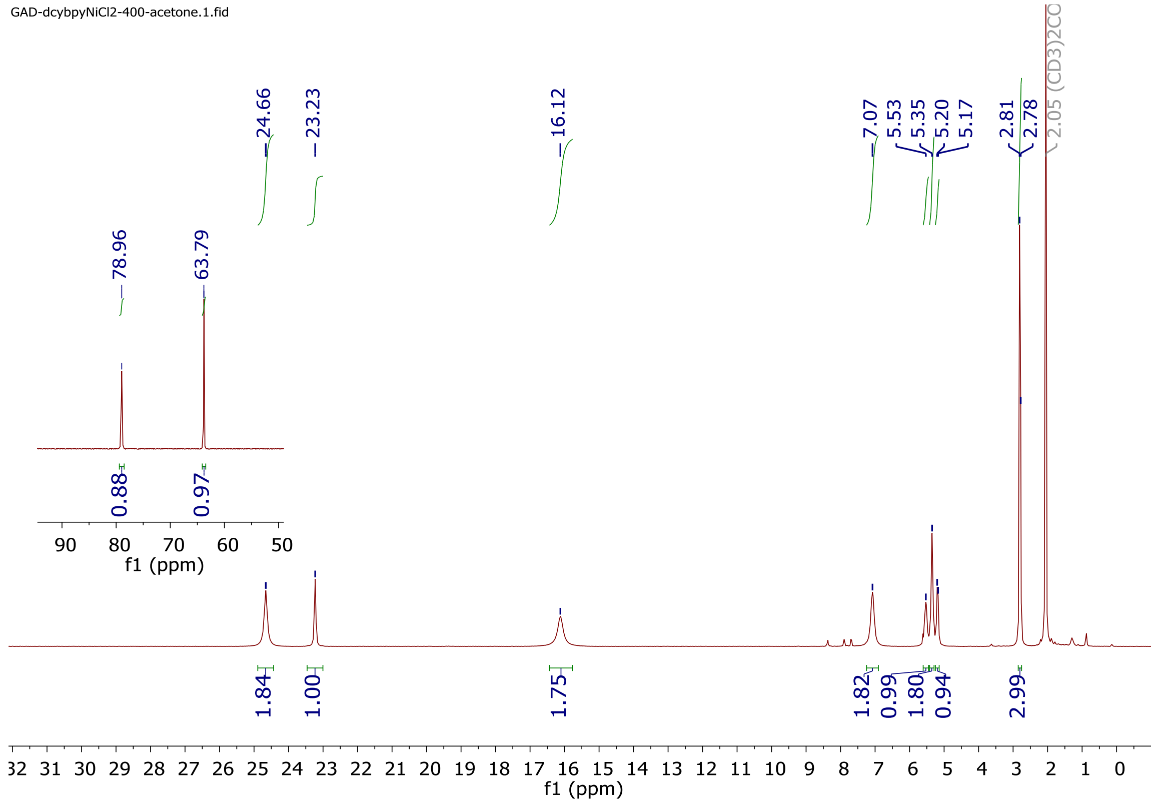


**Figure S20.** $^{1}$H NMR (400 MHz, acetone-$d_6$, 23 °C) of ($^{Cy}$bpy)NiCl$_2$ **11**.

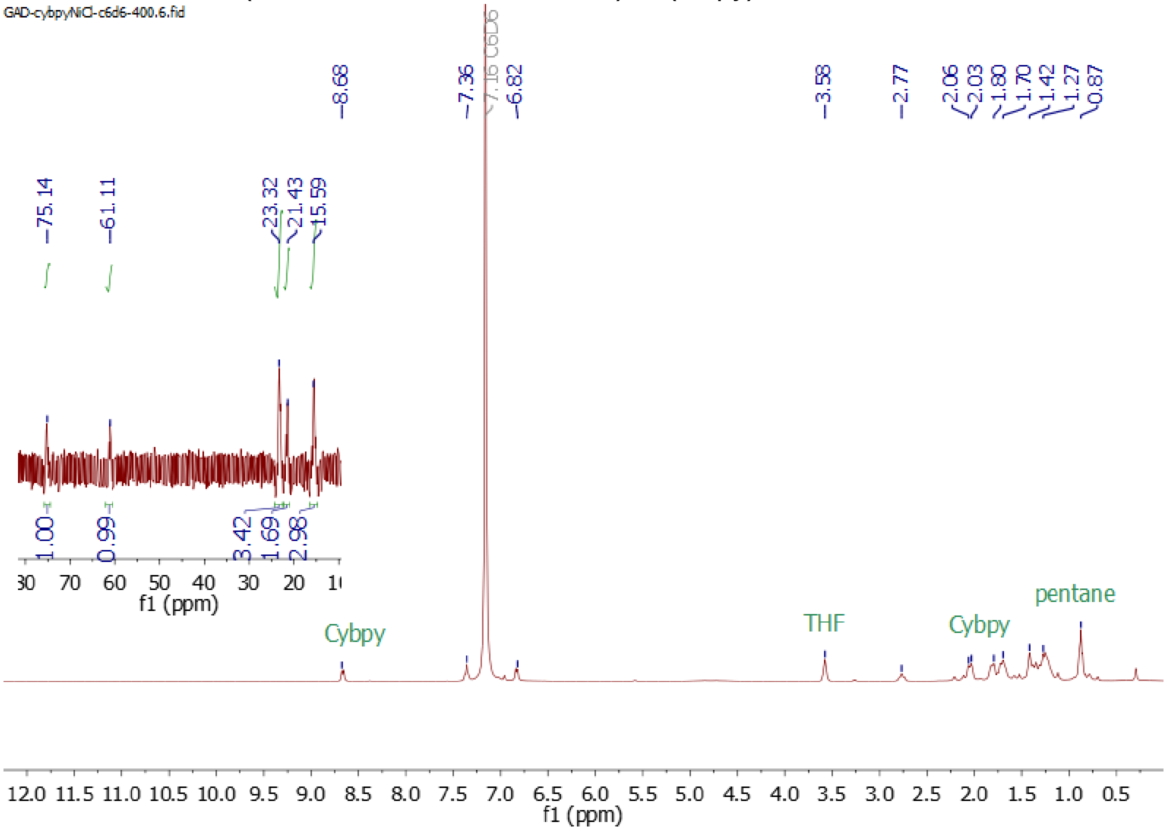


**Figure S21.** $^{1}$H NMR (400 MHz, benzene-$d_6$, 23 °C) of ($^{Cy}$bpy)NiCl$_2$ **12**.

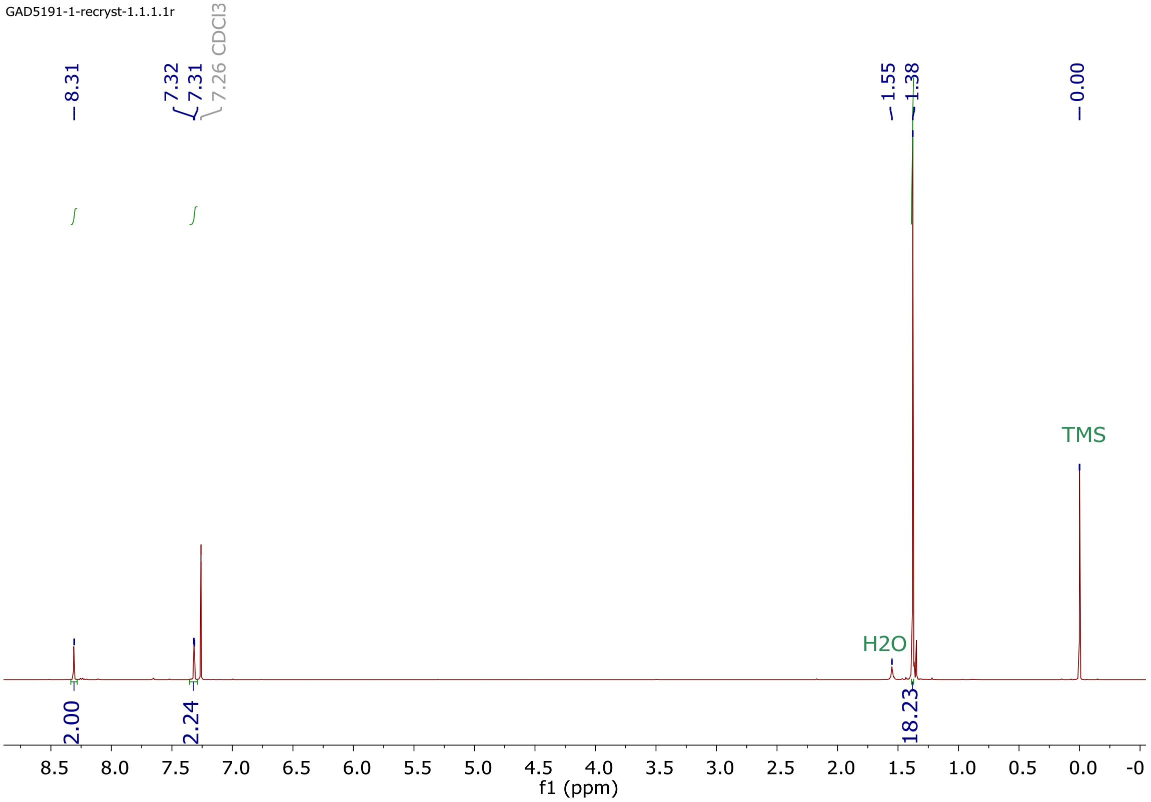


**Figure S22.** $^{1}H$ NMR (400 MHz, chloroform-*d*, 23 °C) of $^{Cl}$dtbpy **S4**.

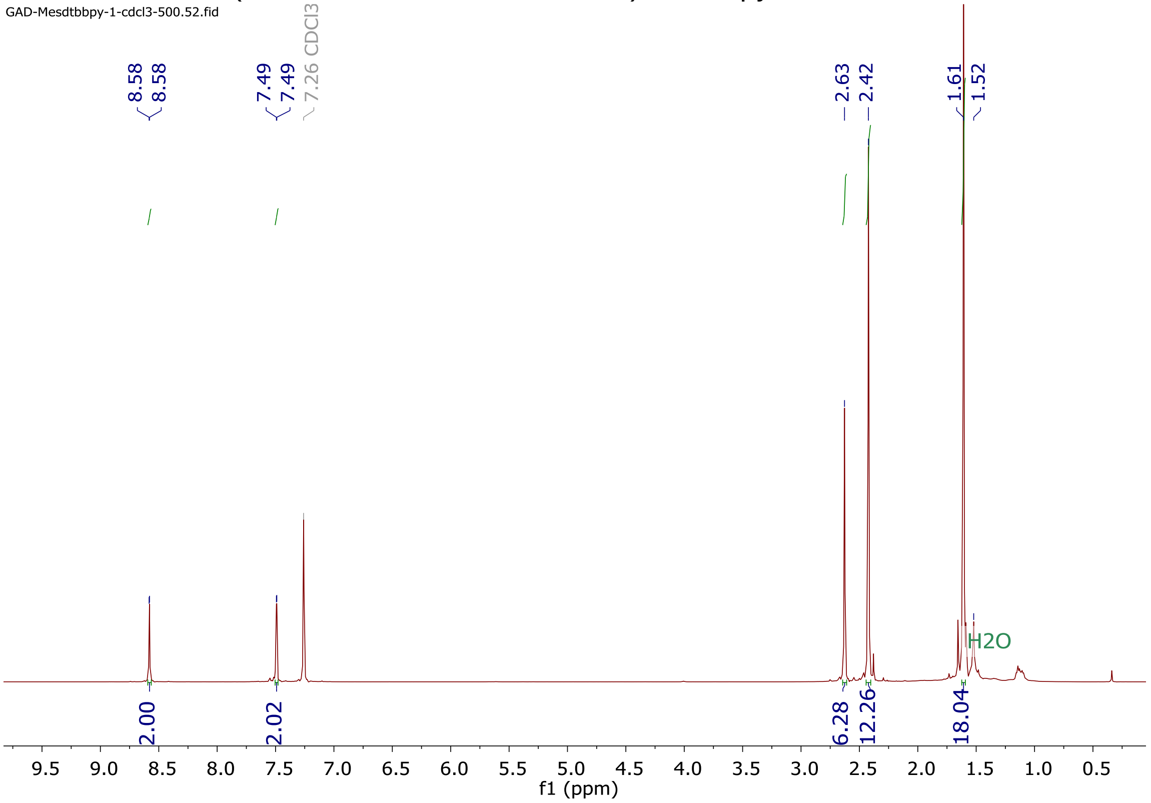


**Figure S23.** $^{1}H$ NMR (500 MHz, chloroform-*d*, 23 °C) of $^{Mes}$dtbpy **S5**.

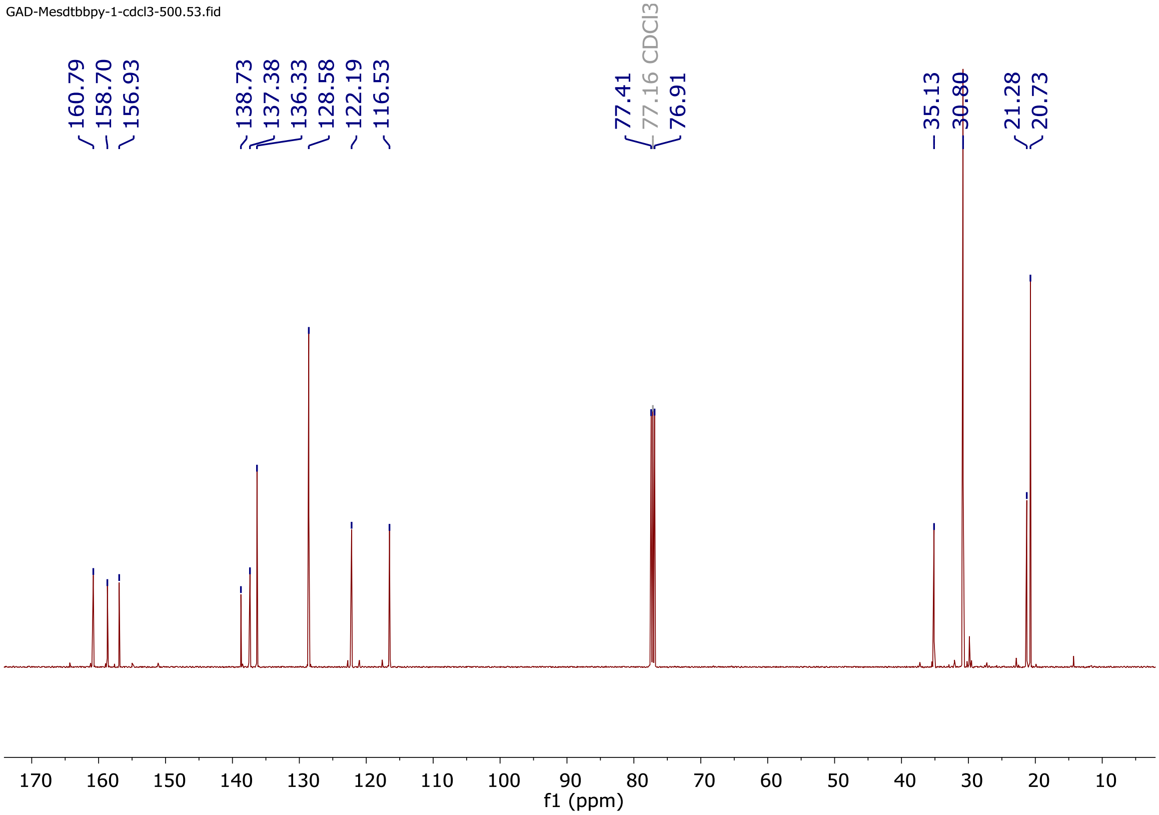


**Figure S24.** $^{13}$C NMR (126 MHz, chloroform-*d*, 23 °C) of $^{Mes}$dtbpy **S5**.

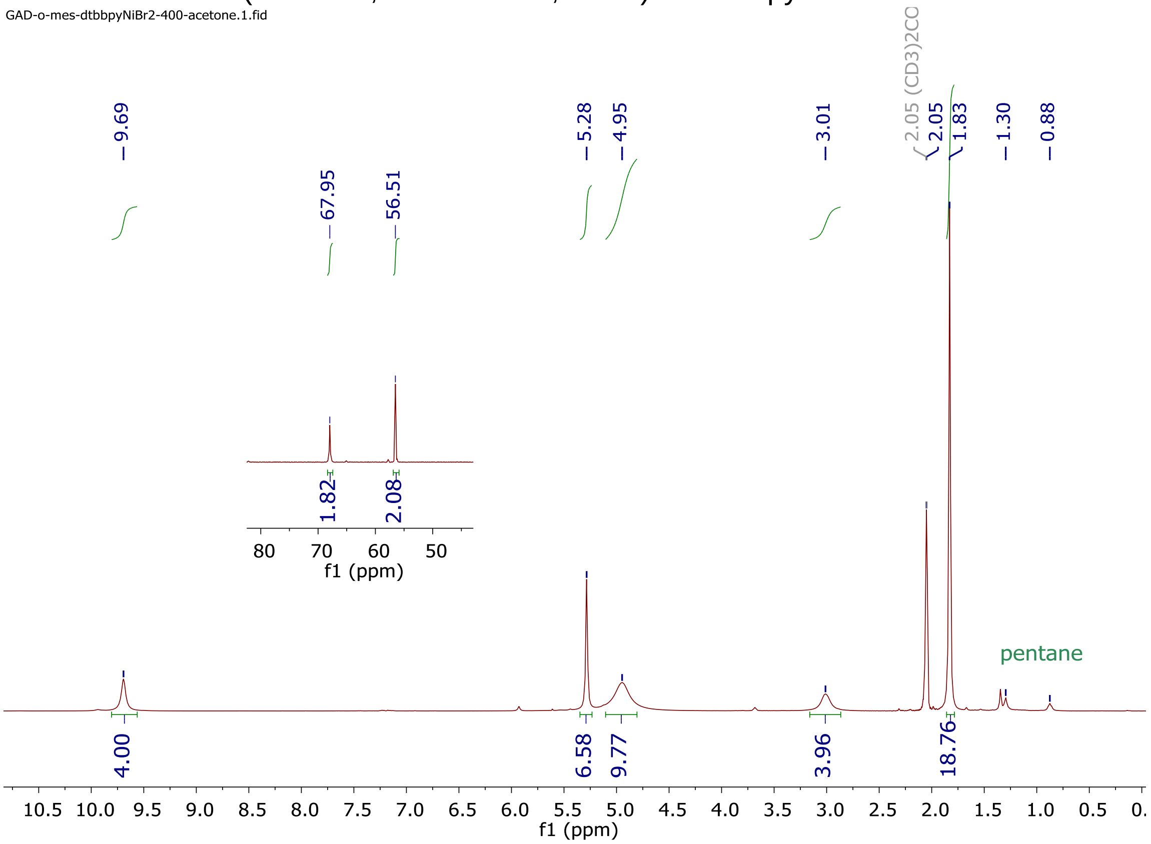


**Figure S25.** $^{1}$H NMR (400 MHz, acetone-*d*$_6$, 23 °C) of ($^{Mes}$dtbpy)NiBr$_2$ **S6**.

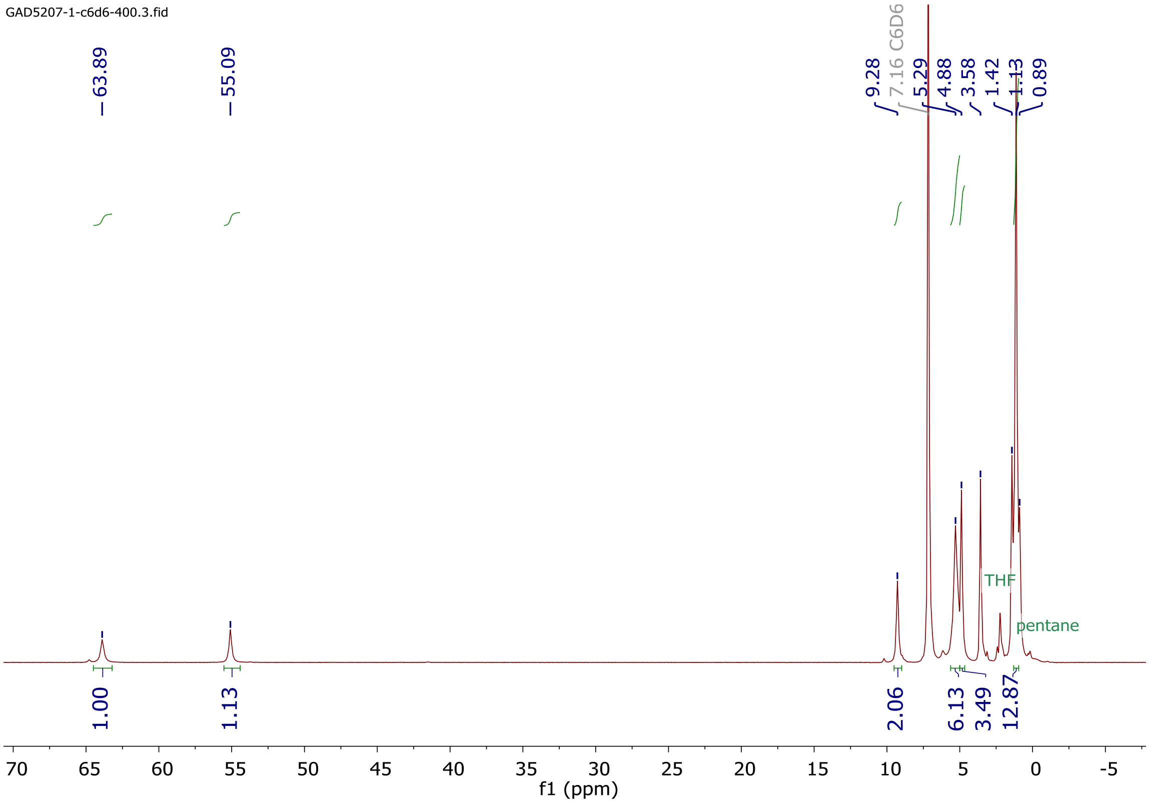


**Figure S26.** $^{1}H$ NMR (400 MHz, benzene-$d_6$, 23 °C) of ($^{Mes}$dtbpy)NiBr **13**.

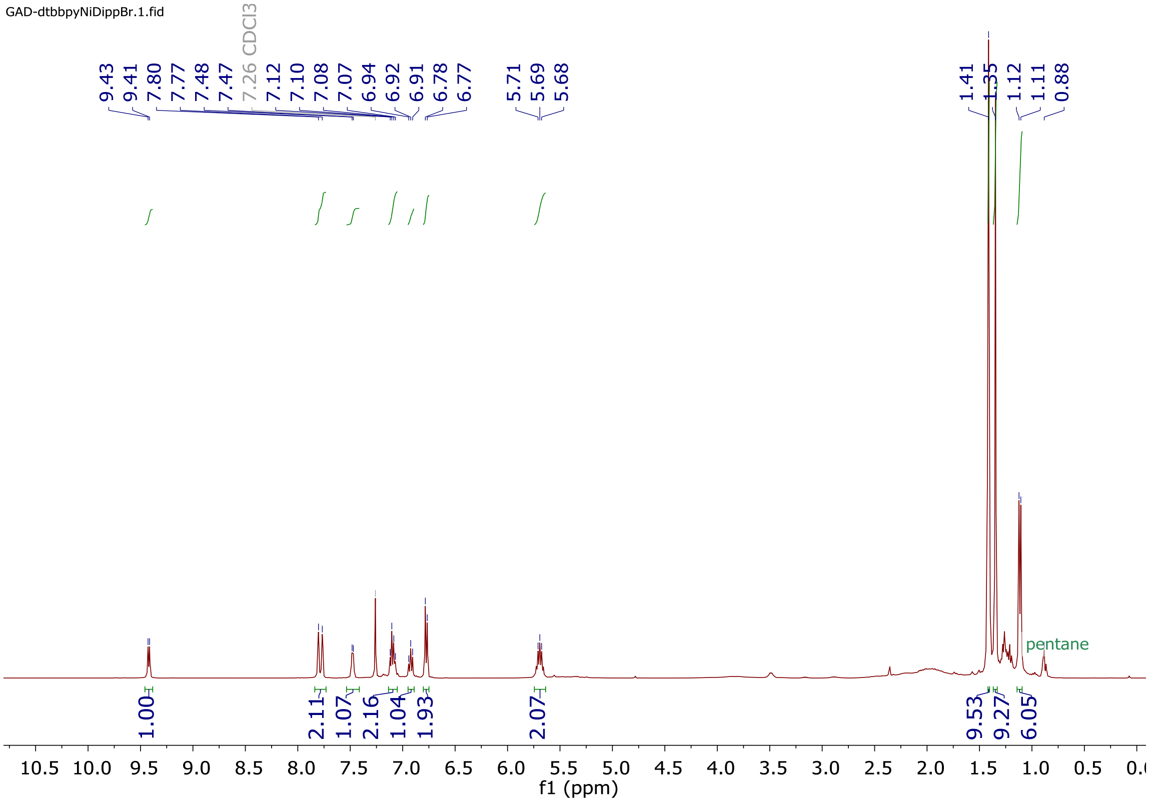


**Figure S27.** $^{1}H$ NMR (400 MHz, chloroform-$d$, 23 °C) of (dtbpy)Ni(Dipp)Br **15**.

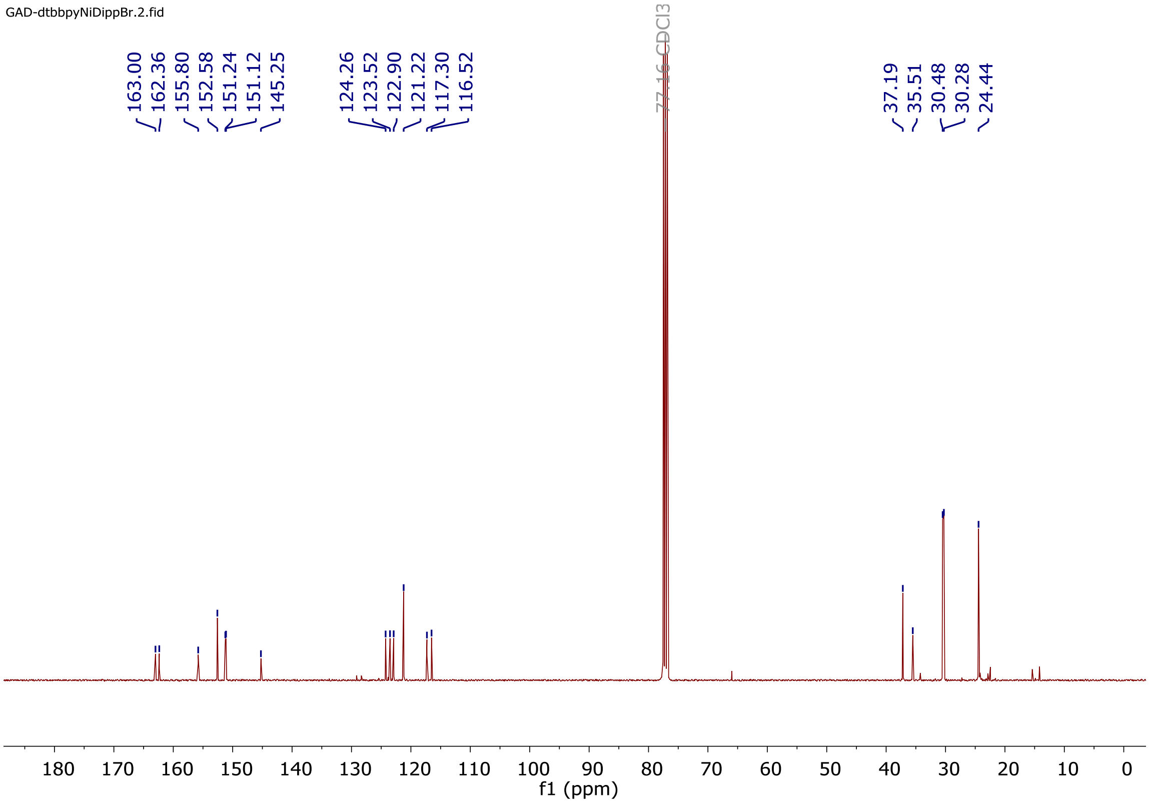


**Figure S28.** $^{13}C$ NMR (101 MHz, chloroform-*d*, 23 °C) of (dtbpy)Ni(Dipp)Br **15**.

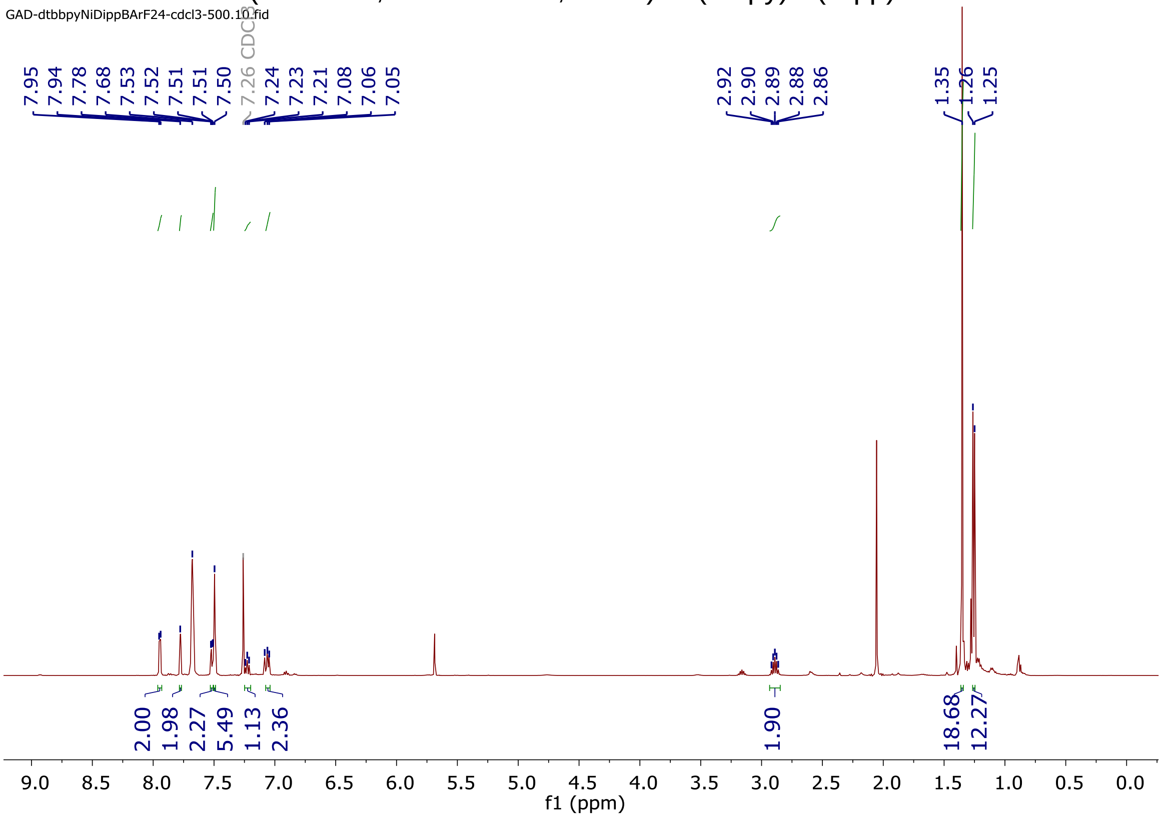


**Figure S29.** $^{1}H$ NMR (500 MHz, chloroform-*d*, 23 °C) of [(dtbpy)Ni(Dipp)][$BAr^{F}_{24}$] **16**.

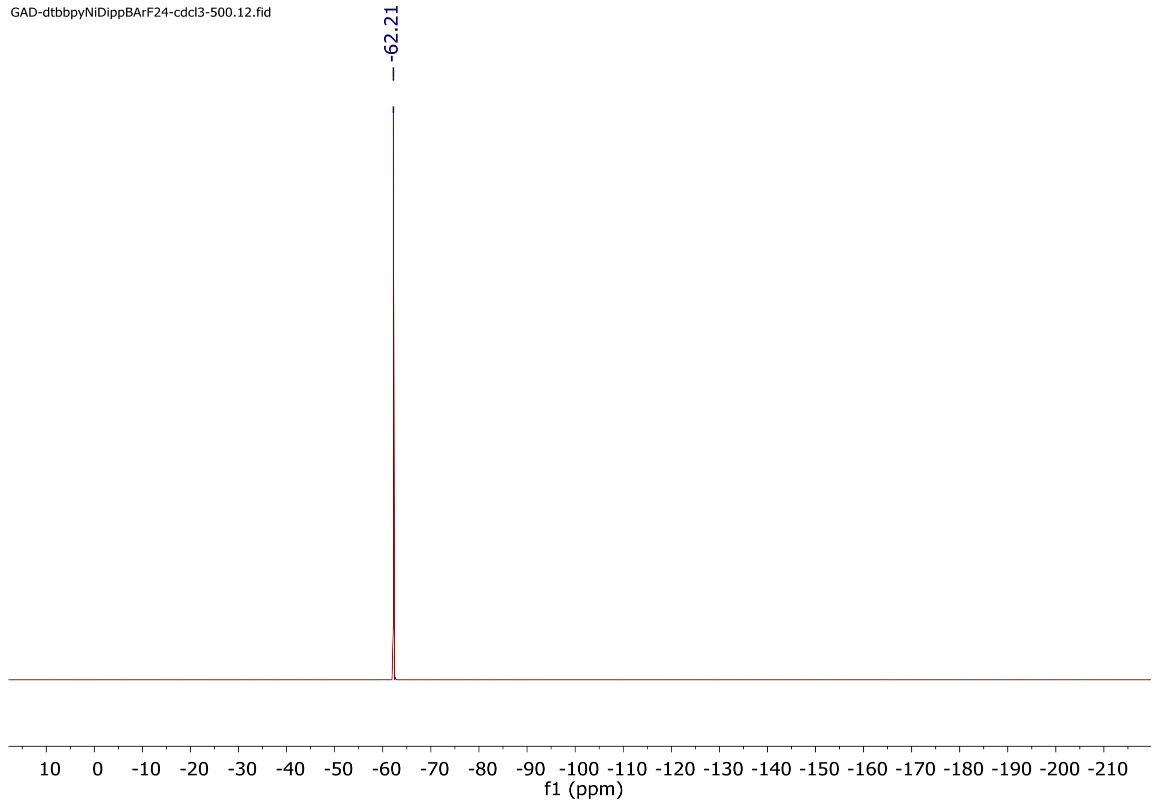


**Figure S30.** $^{19}F$ NMR (470 MHz, chloroform-*d*, 23 °C) of [(dtbpy)Ni(Dipp)][$BAr^F_{24}$] **16**.

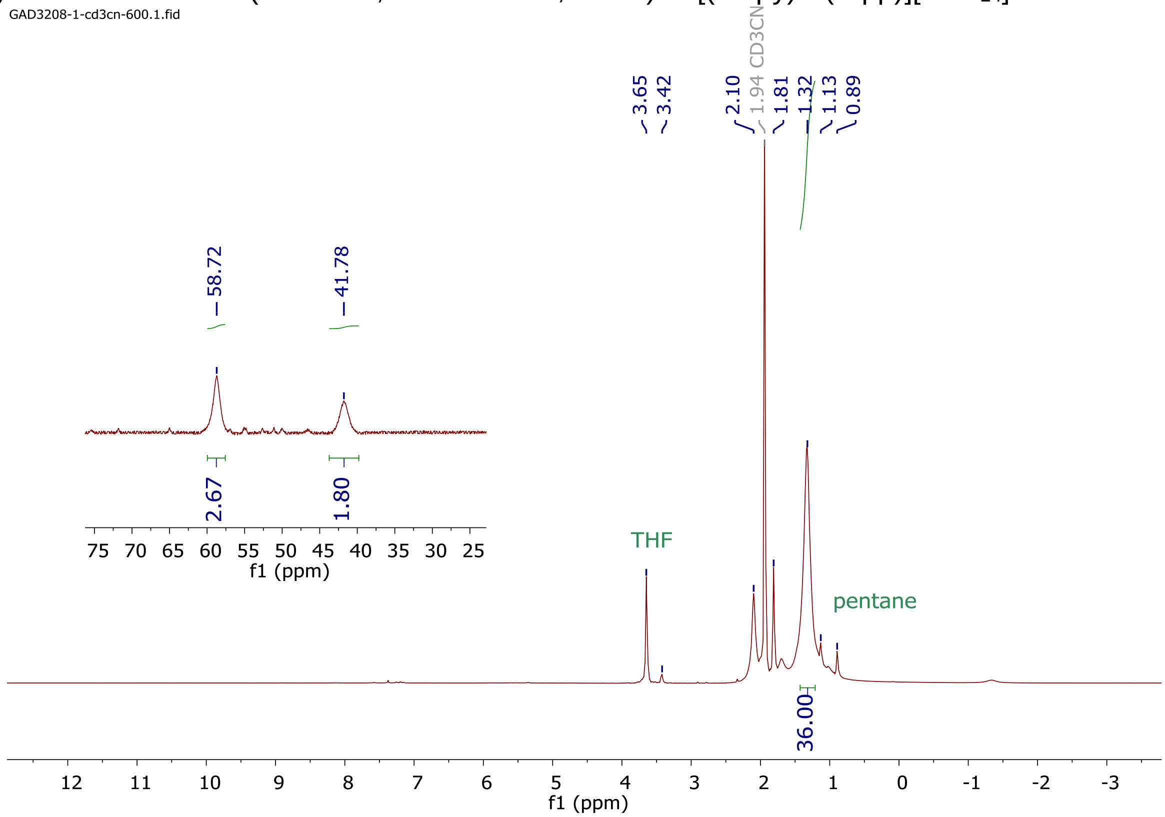


**Figure S31.** $^{1}H$ NMR (600 MHz, acetonitrile-$d_3$, 23 °C) of [(dtbpy)Ni(μ-Cl)$]_2$ **1**.

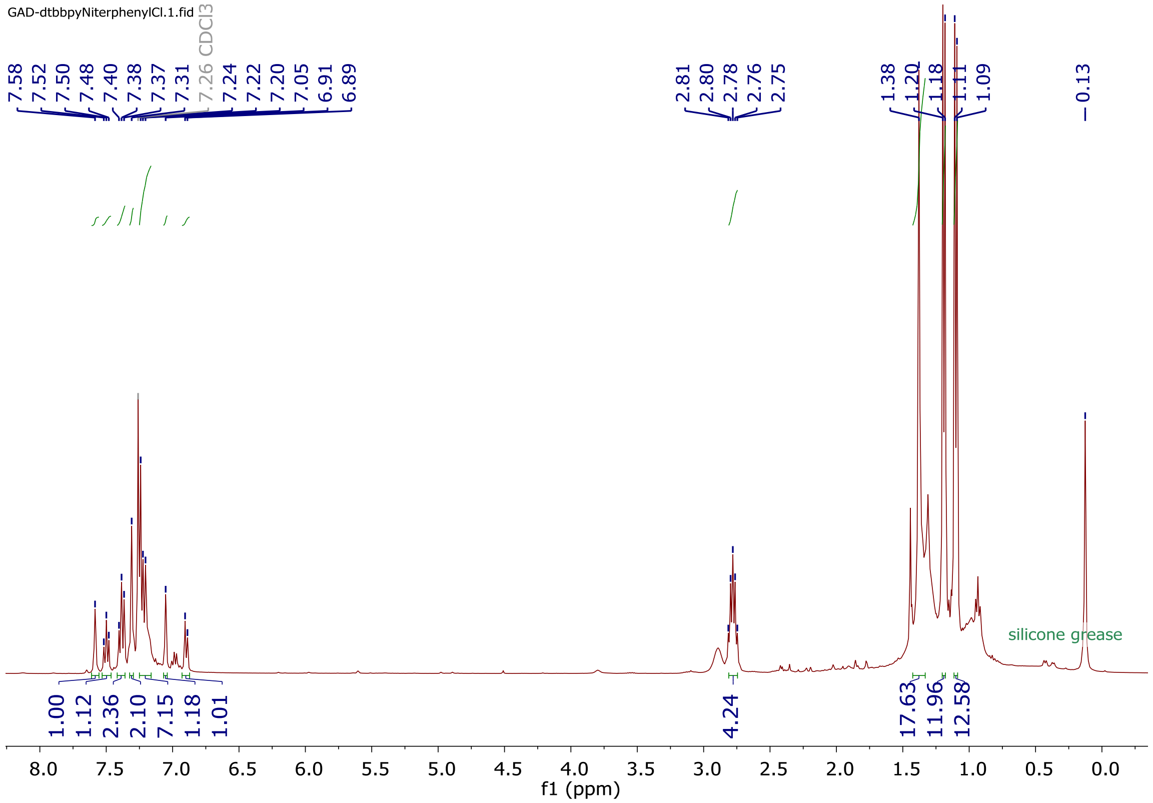

**Figure S32.** $^{1}H$ NMR (400 MHz, chloroform-*d*, 23 °C) of (dtbpy)Ni(Dipp*)Cl **19**.

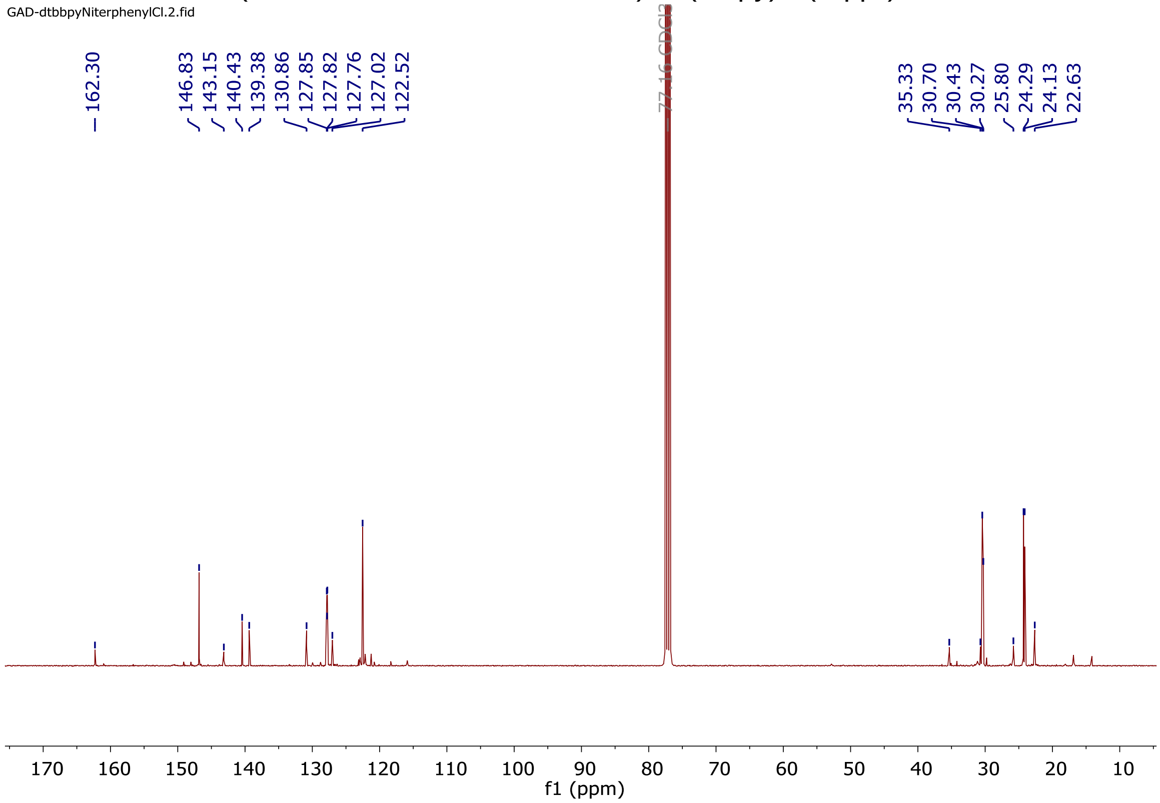

**Figure S33.** $^{13}C$ NMR (101 MHz, chloroform-*d*, 23 °C) of (dtbpy)Ni(Dipp*)Cl **19**.

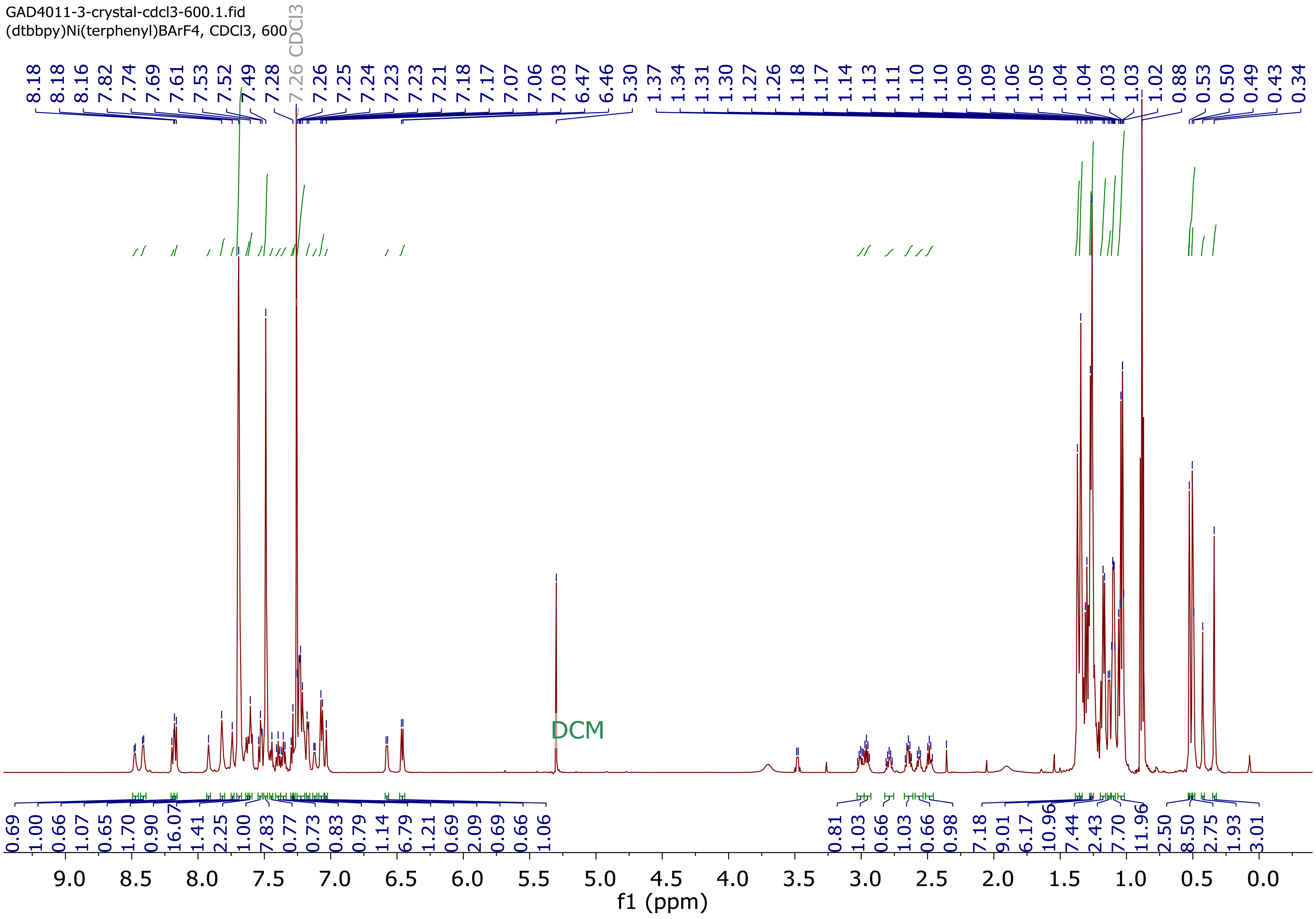


**Figure S34.** $^{1}H$ NMR (600 MHz, chloroform-*d*, 23 °C) of [(dtbpy)Ni(η-3 Dipp*)][$BAr^{F}_{24}$] **22**.

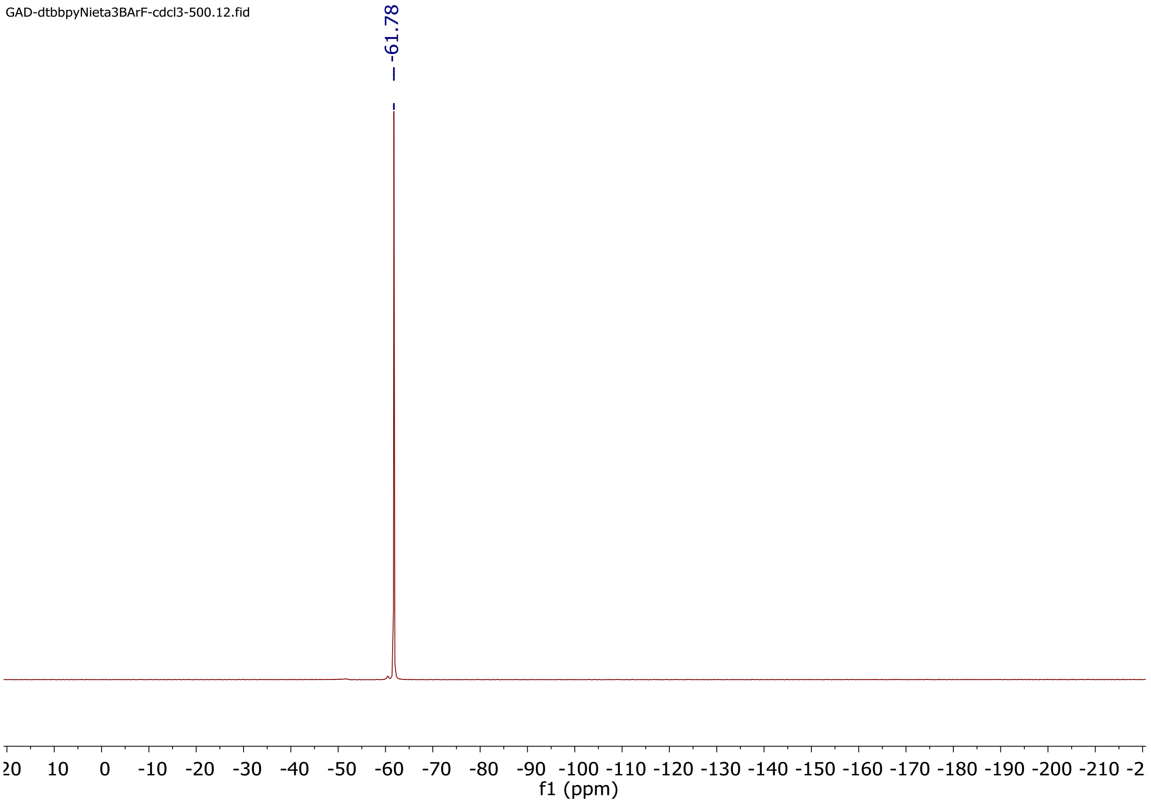


**Figure S35.** $^{19}F$ NMR (470 MHz, chloroform-*d*, 23 °C) of [(dtbpy)Ni(η-3 Dipp*)][$BAr^F{}_{24}$] **22**.

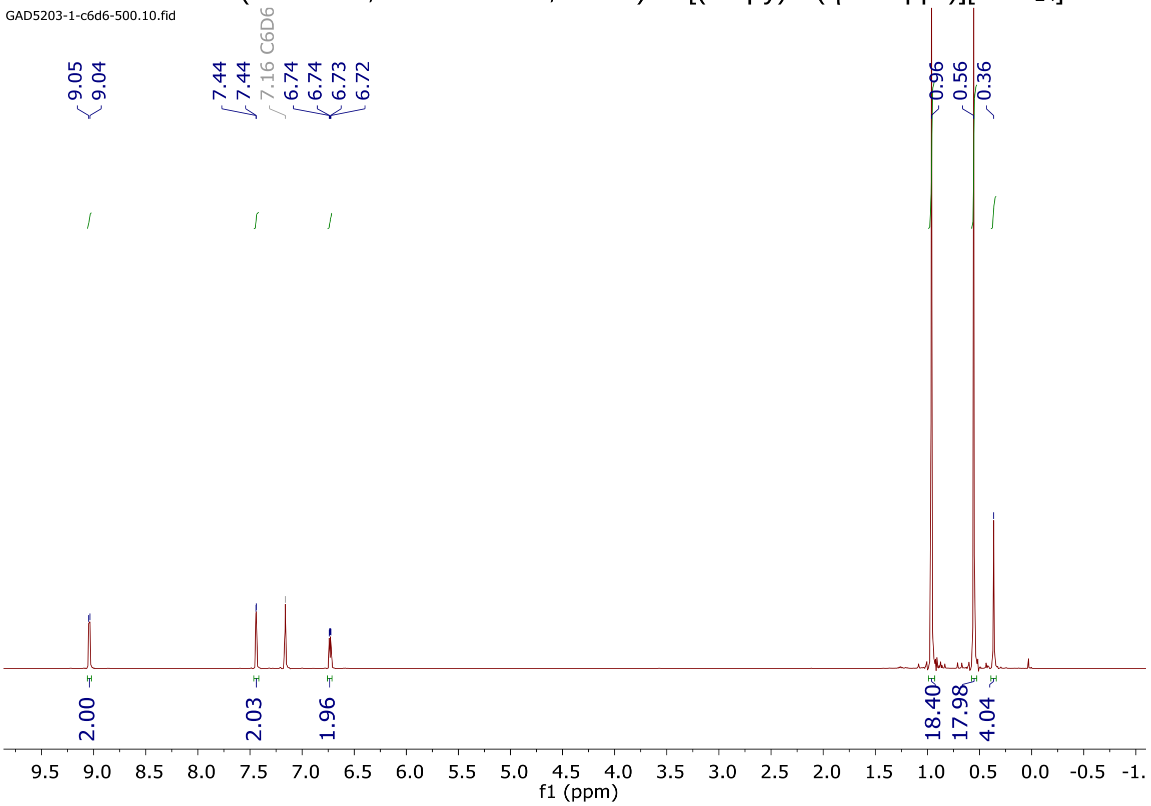


**Figure S36.** $^{1}H$ NMR (500 MHz, benzene-$d_6$, 23 °C) of (dtbpy)Ni($CH_2$TMS)$_2$ **25**.

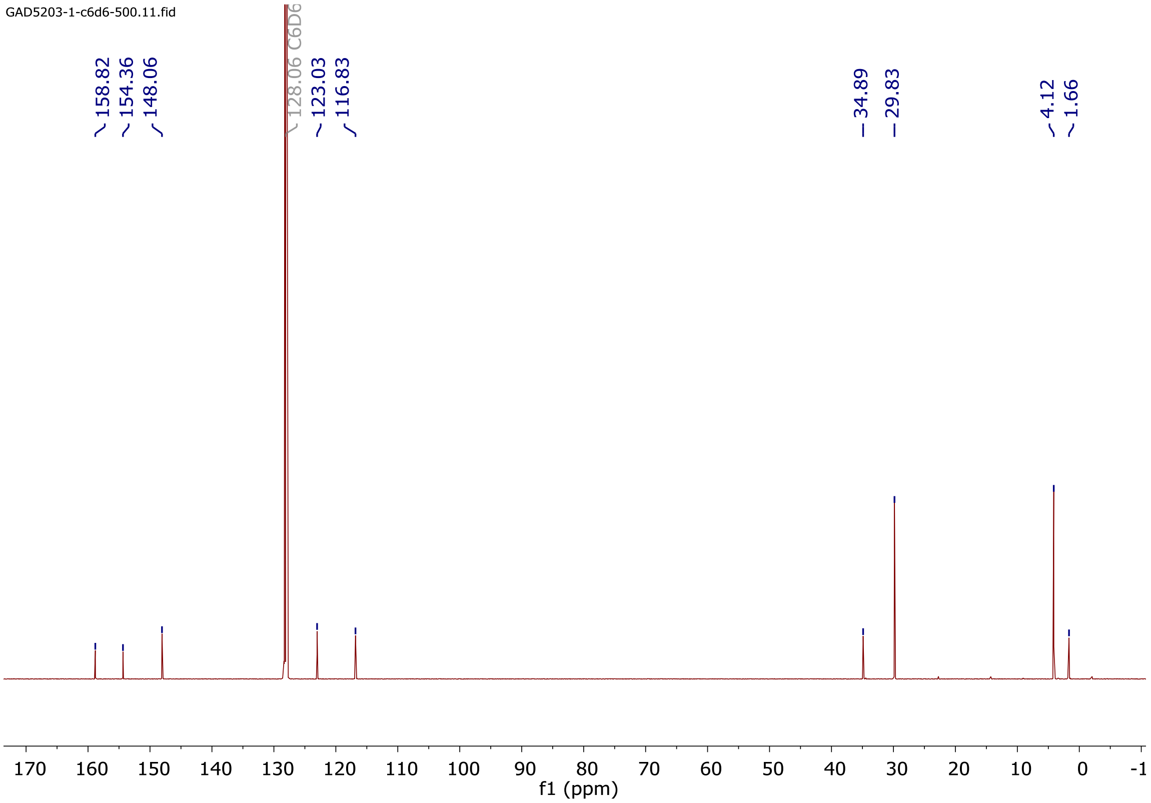


**Figure S37.** $^{13}$C NMR (126 MHz, benzene-$d_6$, 23 °C) of (dtbpy)Ni($CH_2$TMS)$_2$ **25**.

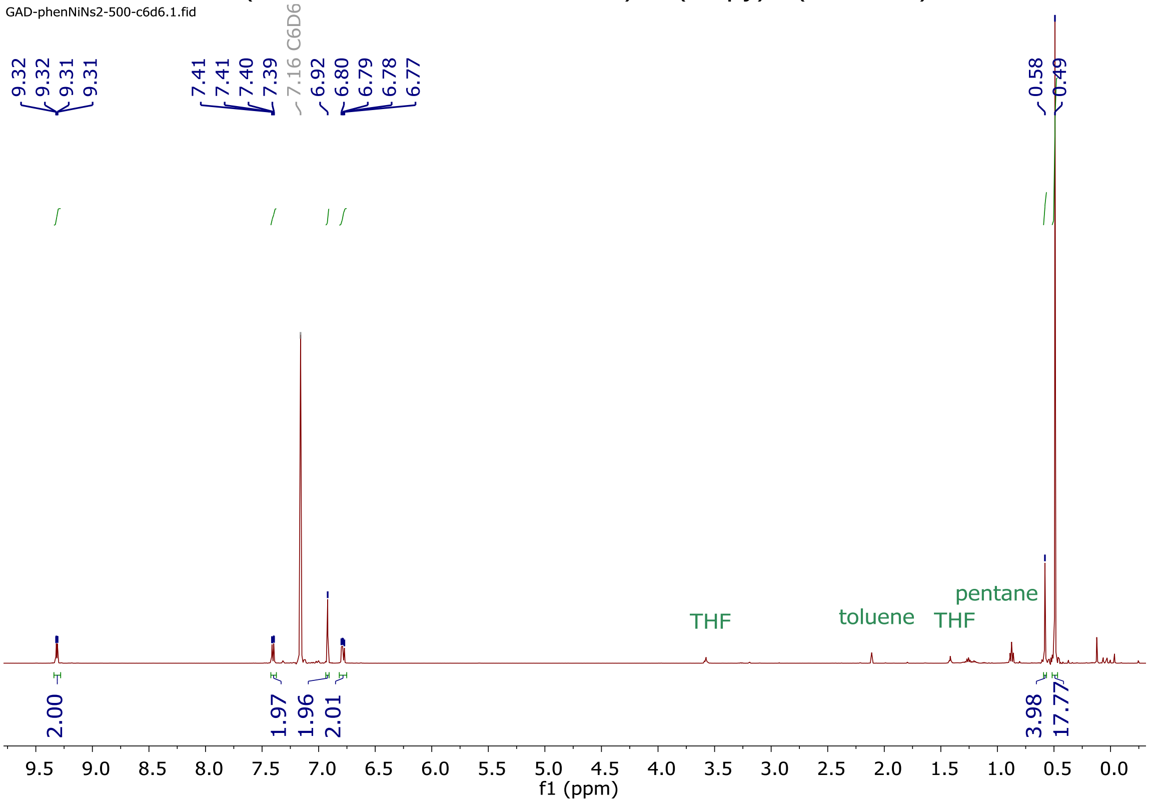


**Figure S38.** $^{1}$H NMR (500 MHz, benzene-$d_6$, 23 °C) of (phen)Ni($CH_2$TMS)$_2$ **29**.

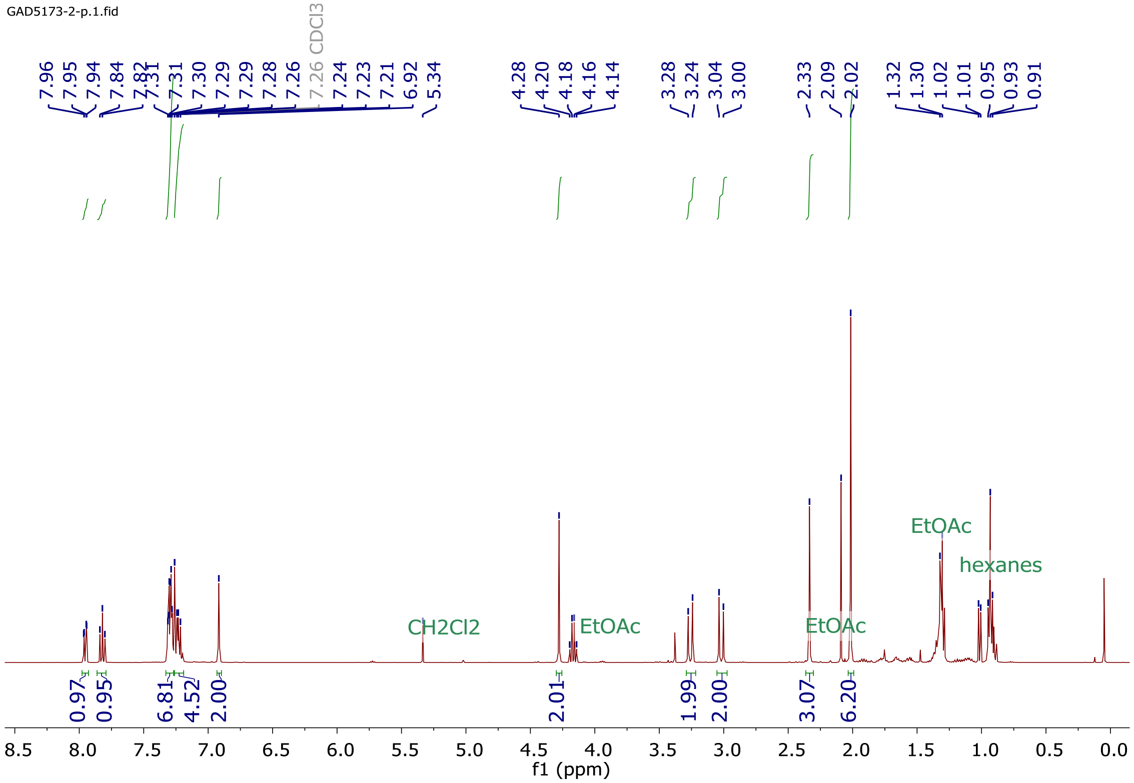


**Figure S39.** $^{1}$H NMR (400 MHz, chloroform-*d*, 23 °C) of 6-Mes-$^{dBn}$picolinamide **S7**.

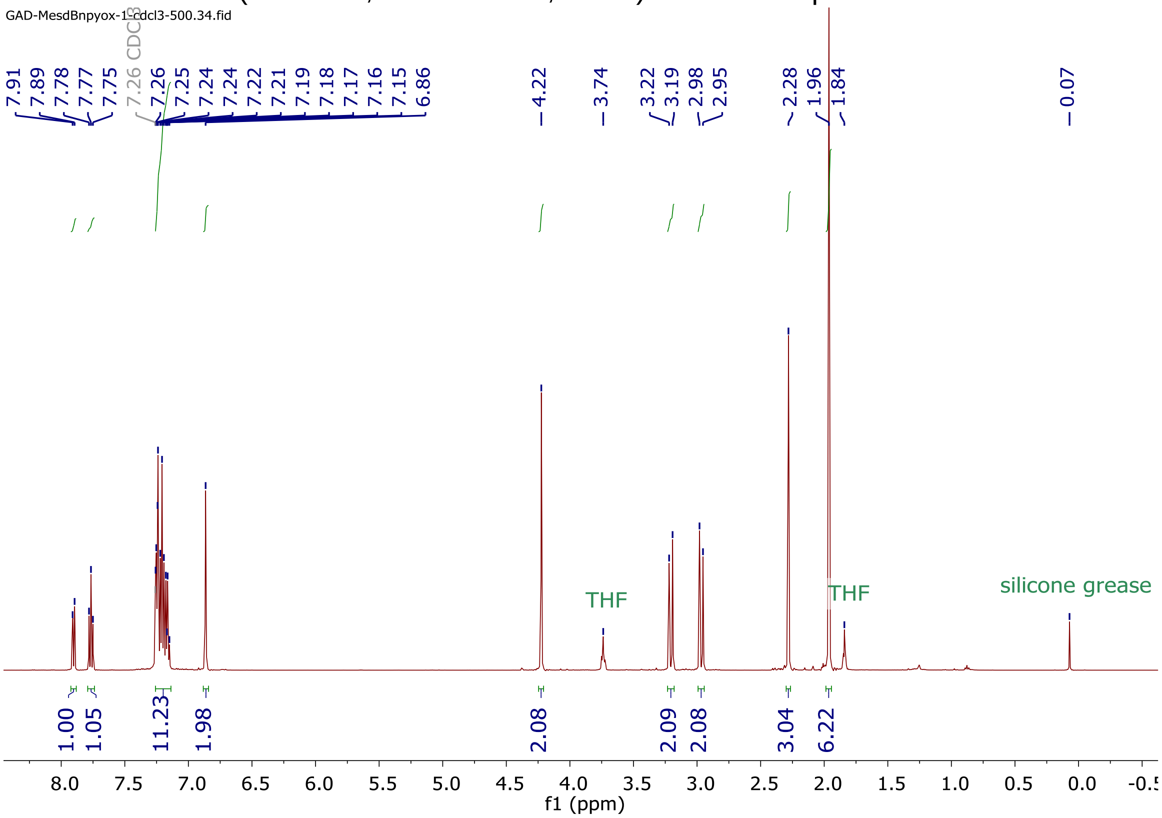


**Figure S40.** $^{1}$H NMR (500 MHz, chloroform-*d*, 23 °C) of 6-Mes-$^{dBn}$pyrox **S8**.

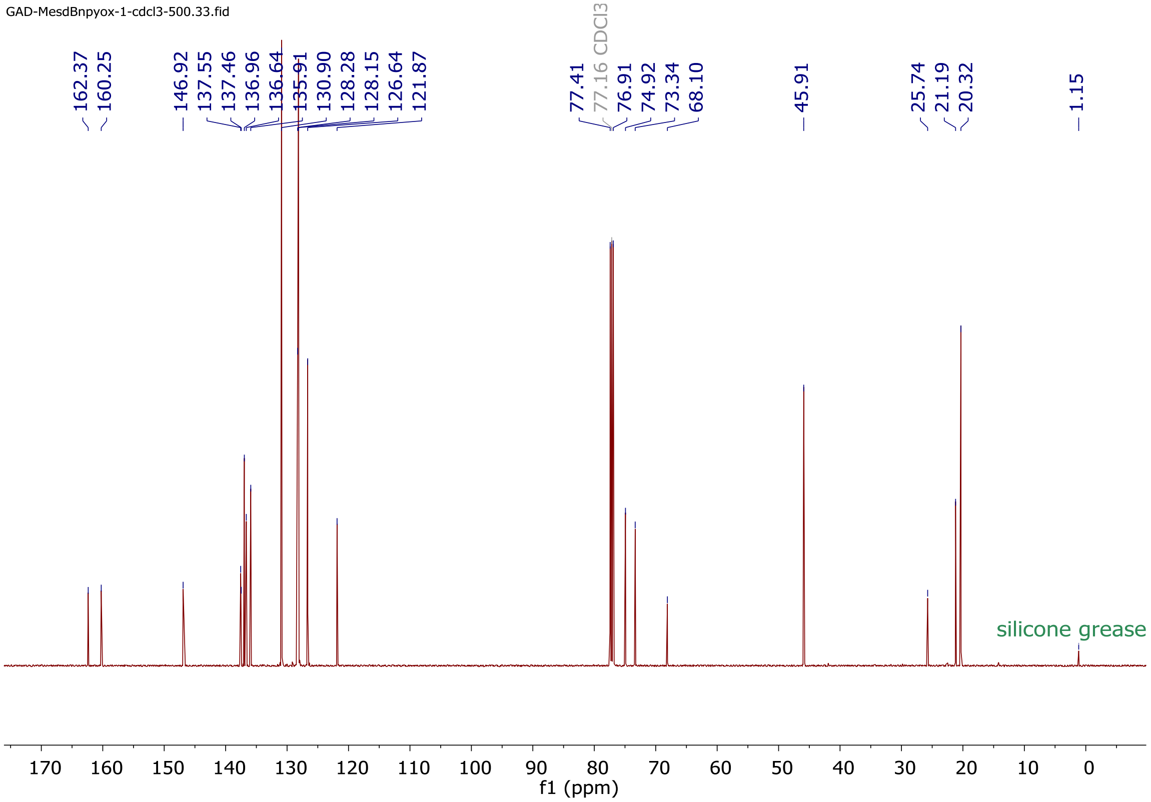


**Figure S41.** $^{13}C$ NMR (126 MHz, chloroform-*d*, 23 °C) of 6-Mes-$^{dBn}$pyrox **S8**.

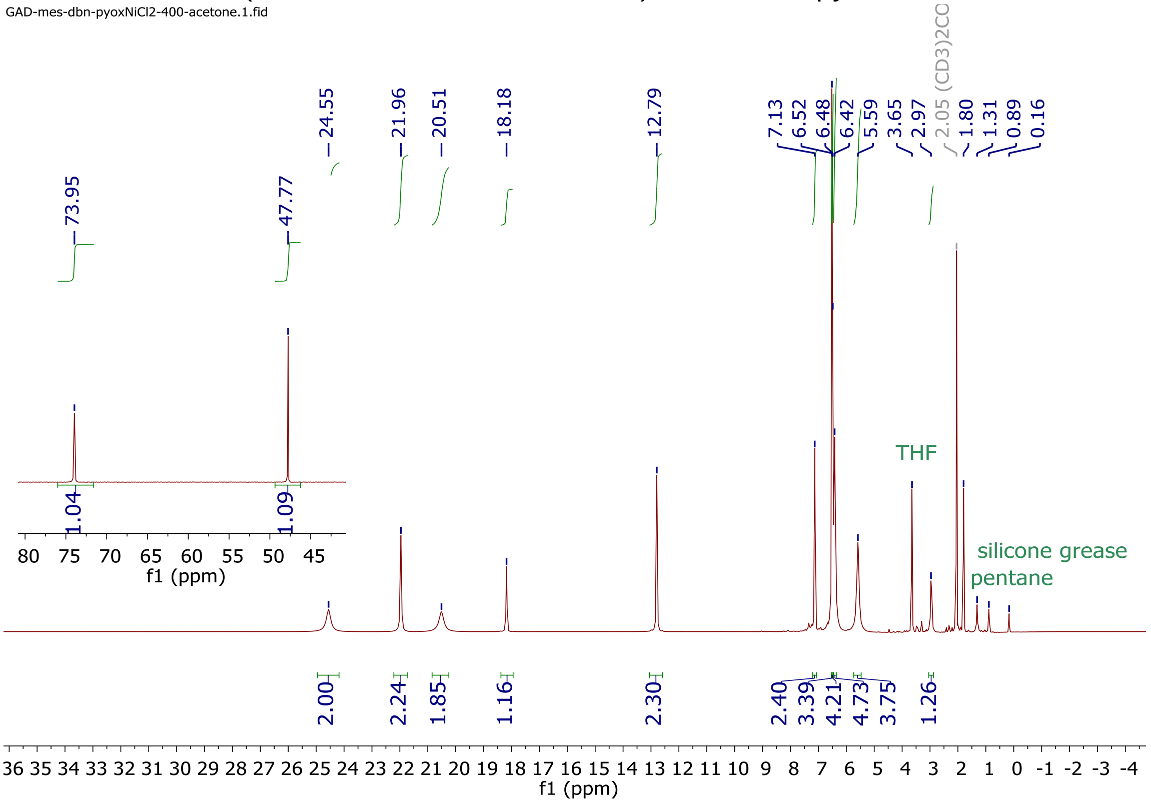


**Figure S42.** $^{1}H$ NMR (400 MHz, acetone-$d_6$, 23 °C) of (6-Mes-$^{dBn}$pyrox)$NiCl_2$ **31**.

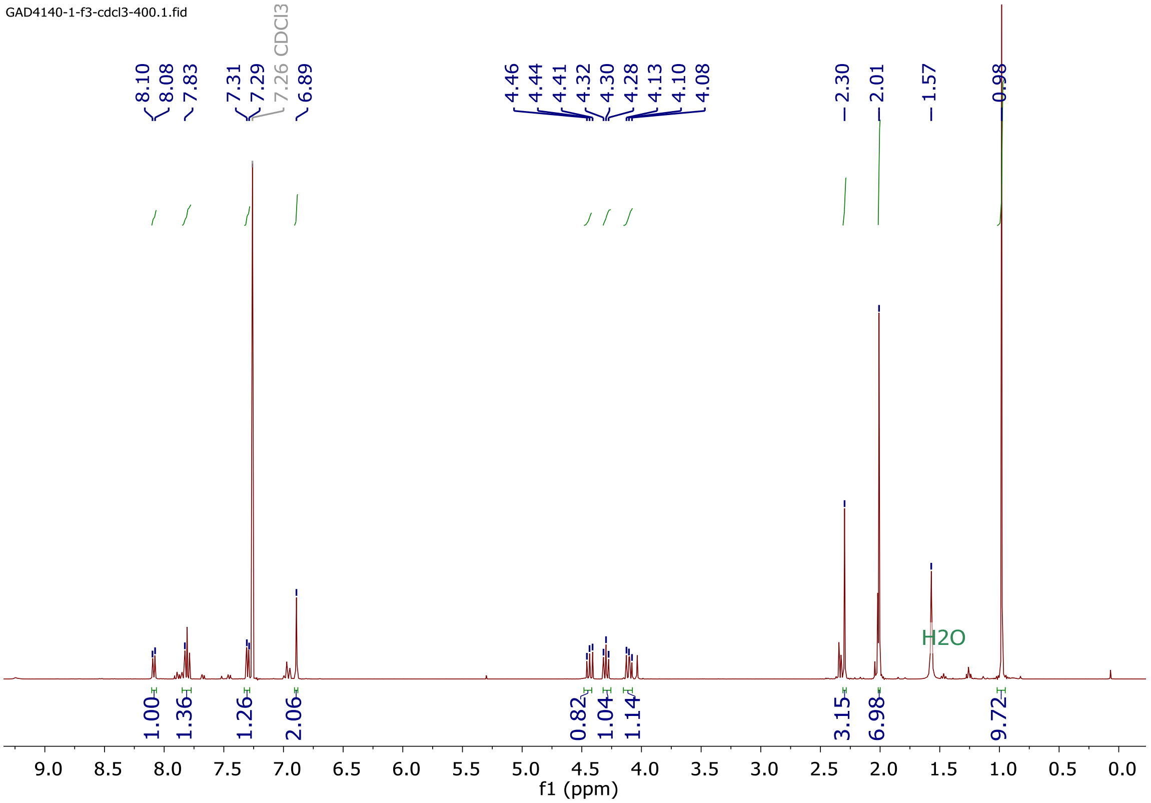

**Figure S43.** $^{1}$H NMR (400 MHz, chloroform-*d*, 23 °C) of 6-Mes-$^{tBu}$pyrox **S9**.

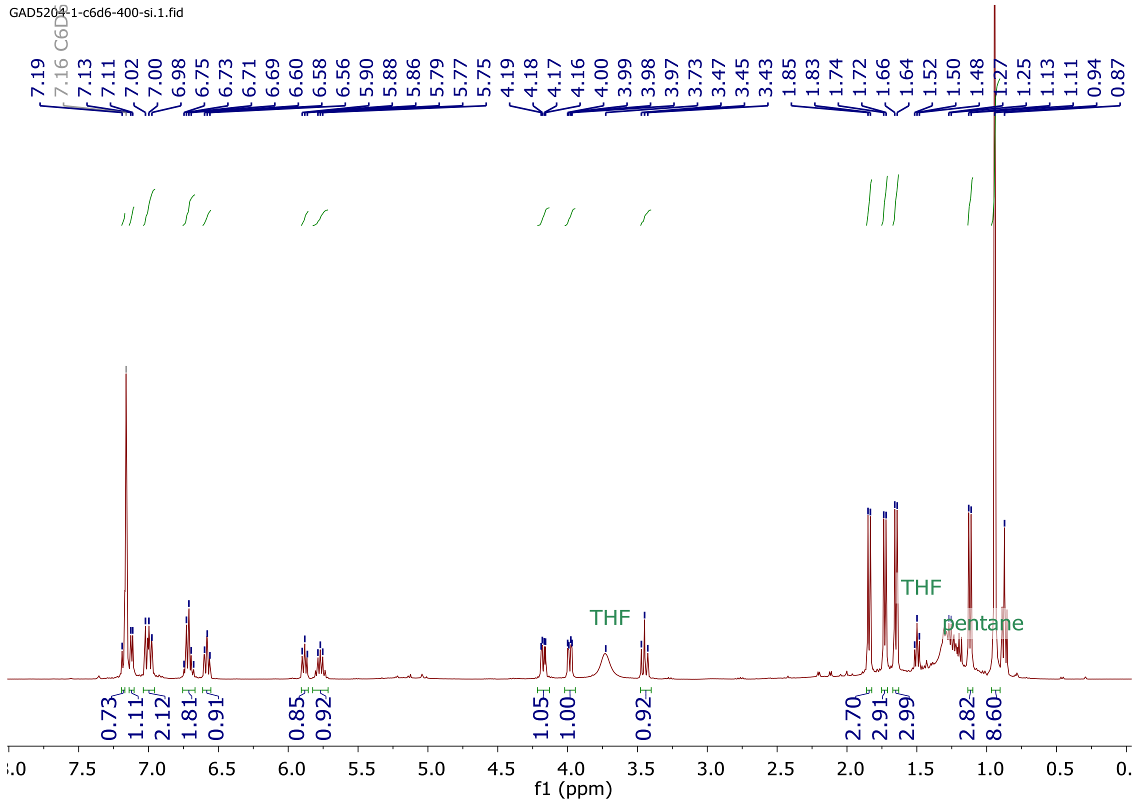

**Figure S44.** $^{1}$H NMR (400 MHz, benzene-$d_6$, 23 °C) of ($^{tBu}$pyrox)Ni(Dipp)Br **33**.

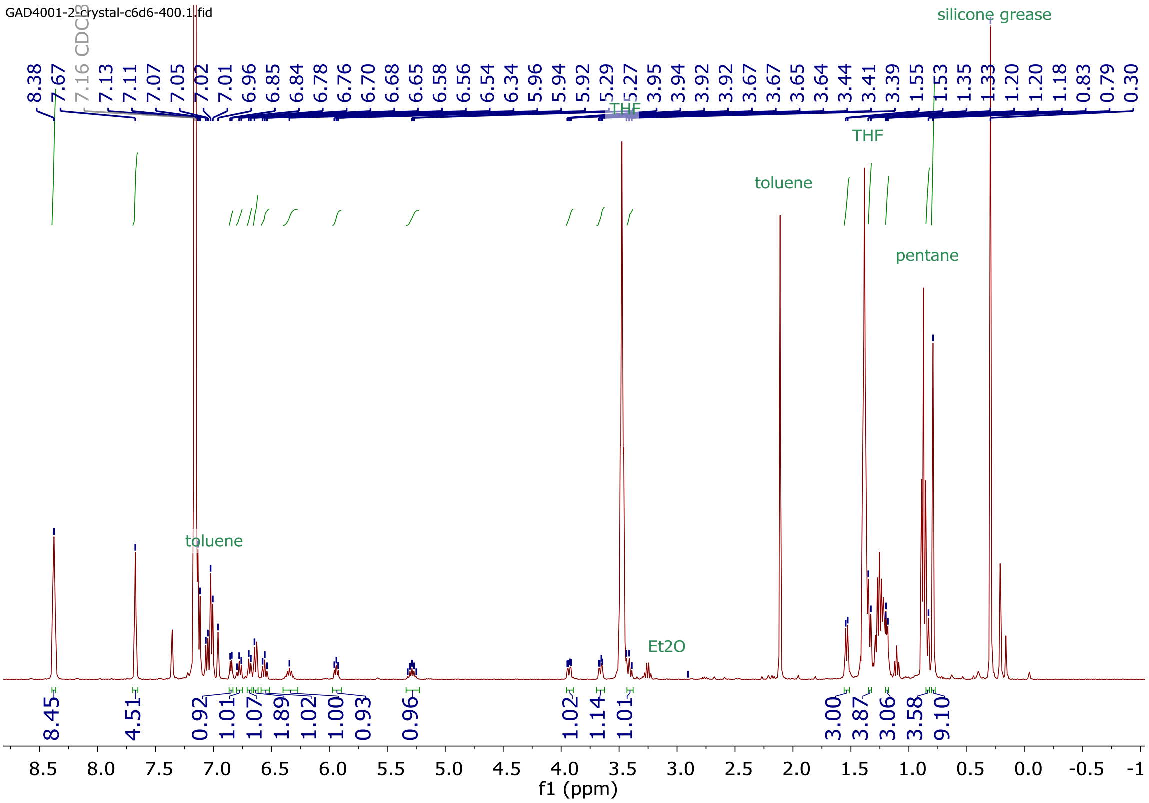


**Figure S45.** $^{1}$H NMR (400 MHz, chloroform-*d*, 23 °C) of [($^{t\text{Bu}}$pyrox)Ni(Dipp)][BAr$^{F}_{24}$] **34**.

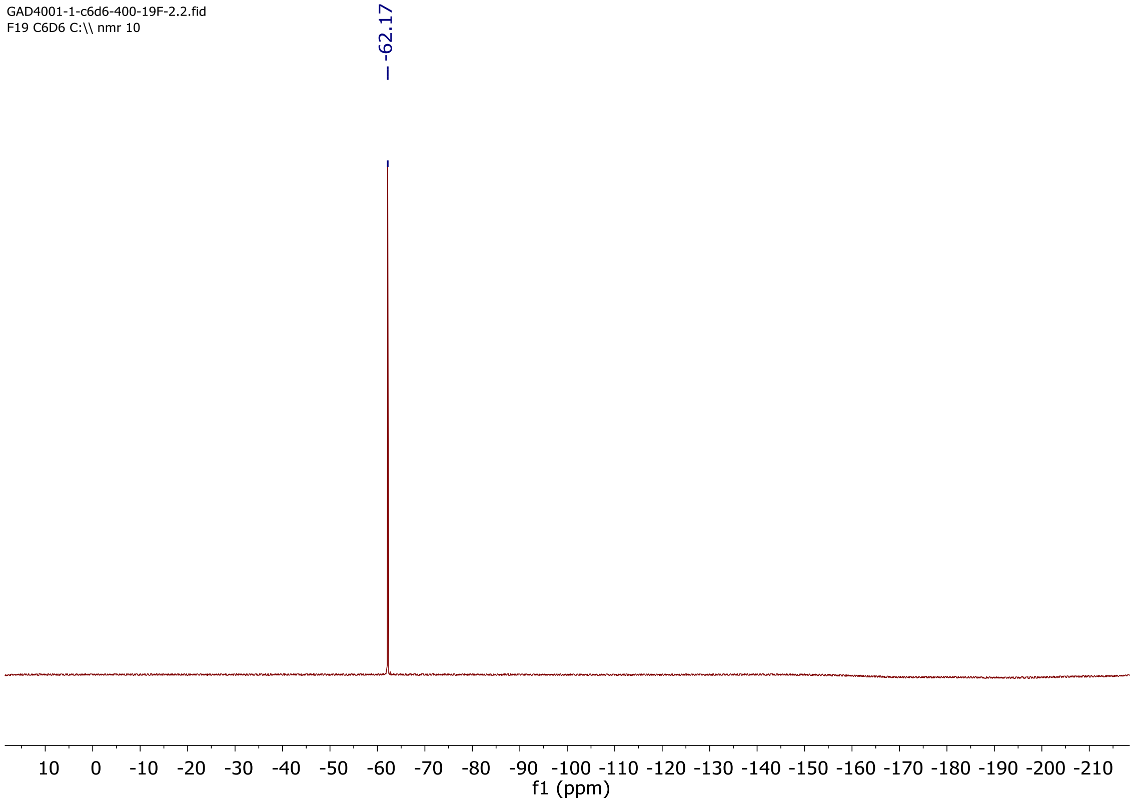


**Figure S46.** $^{19}$F NMR (470 MHz, chloroform-*d*, 23 °C) of [($^{t\text{Bu}}$pyrox)Ni(Dipp)][BAr$^{F}_{24}$] **34**.

## 6. EPR Spectra

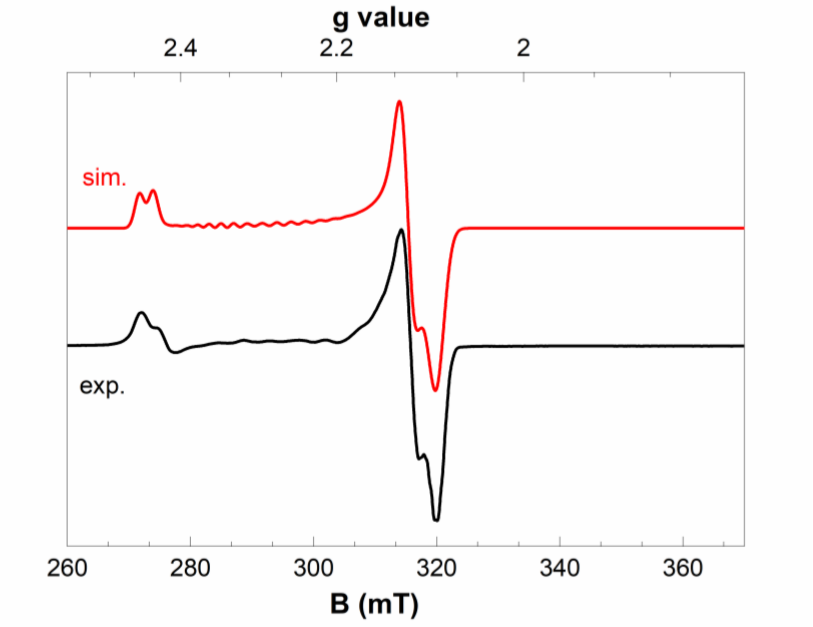


**Figure S47.** X-band EPR spectrum of **12**. Temperature = 15 K, solvent = THF, microwave frequency = 9.35243 GHz, power = 0.004743 mW, modulation amplitude = 4.0 G, modulation frequency = 1 mT/100 kHz. The simulation was performed using Xepr software. The simulated spectrum (red) uses the following parameters: g = [2.450, 2.130, 2.089], $A_{Hx}$ = 22.307 G. $A_{Hy}$ = 0.000 G, $A_{Hz}$ = 0.000 G.

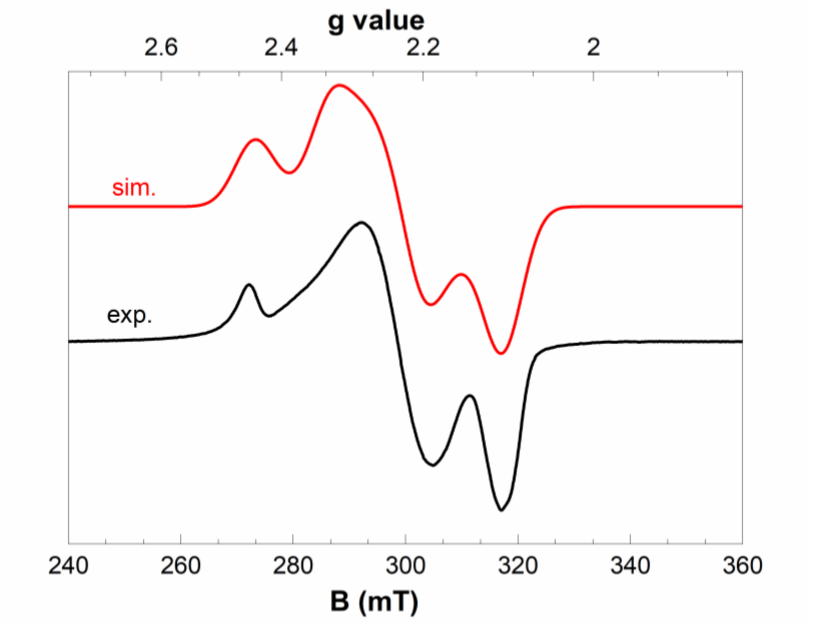


**Figure S48.** X-band EPR spectrum of **S3**. Temperature = 10 K, solvent = THF, microwave frequency = 9.334305 GHz, power = 0.0200 mW, modulation frequency = 1 mT/100 kHz, modulation amplitude = 5.0 G. The simulation was performed using Easyspin in MATLAB. The simulated spectrum (red) uses the following parameters: g =[2.387, 2.236, 2.104], $A_{Hx}$ = 450 MHz, $A_{Hy}$ = 200 MHz, $A_{Hz}$ = 0 MHz.

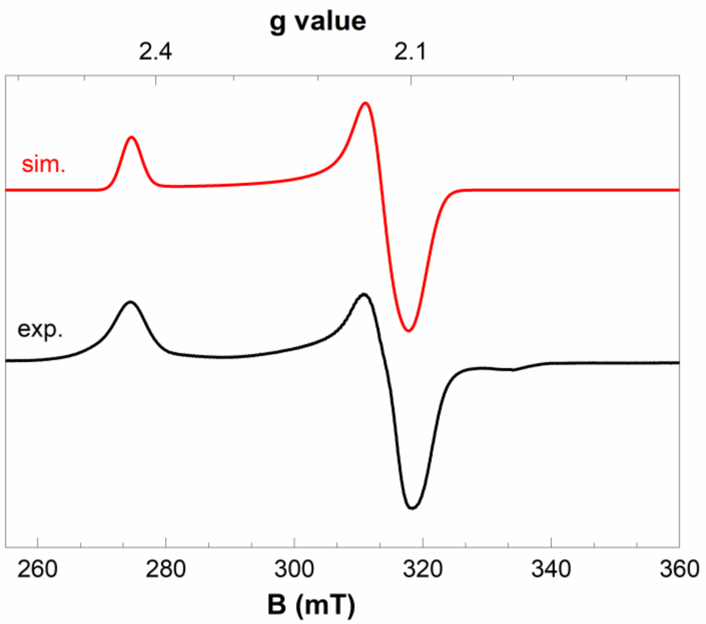


**Figure S49.** X-band EPR spectrum of **13**. Temperature = 15 K, solvent = THF, microwave frequency = 9.351799 GHz, power = 0.47430 mW, modulation amplitude = 4.0 G, modulation frequency = 1 mT/100 kHz. The simulation was performed using Xepr software. The simulated spectrum (red) uses the following parameters: g = [2.434, 2.132, 2.097].

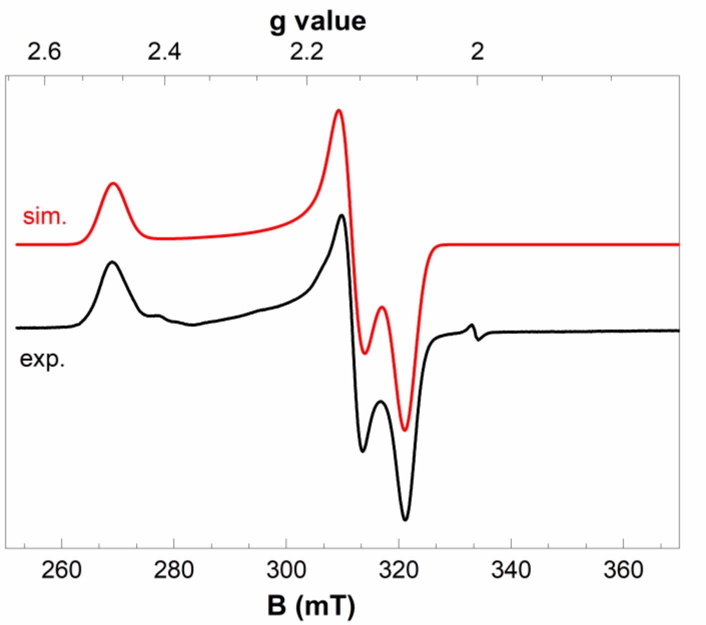


**Figure S50.** X-band EPR spectrum of **14**. Temperature = 30 K, solvent = THF, microwave frequency = 9.349589 GHz, power = 0.47430 mW, modulation amplitude = 2.0 G. modulation frequency = 1 mT/100 kHz. The simulation was performed using Xepr software. The simulated spectrum (red) uses the following parameters: g = [2.485, 2.145, 2.081].

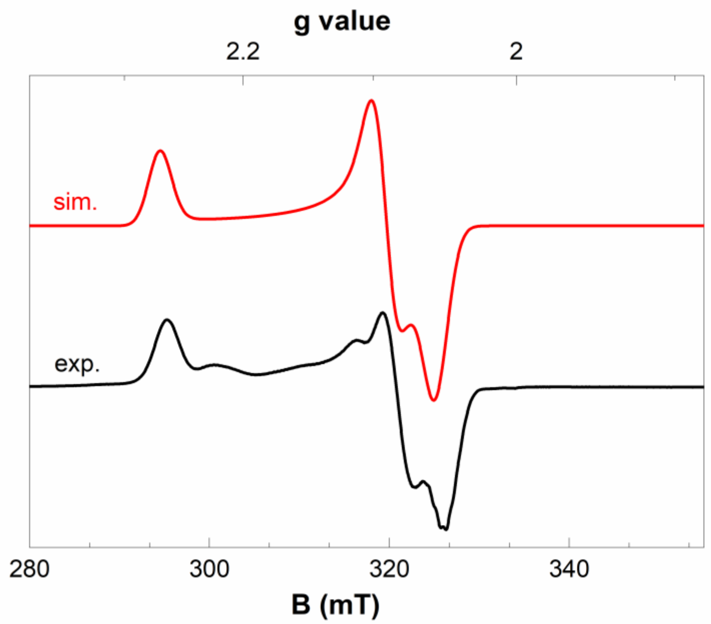


**Figure S51.** X-band EPR spectrum of $KC_8$ reduction of **16** (assigned **17**). Temperature = 30 K, solvent = $Et_2O$, microwave frequency = 9.352851 GHz, power = 0.4743 mW, modulation amplitude = 4.0 G. modulation frequency = 1 mT/100 kHz. The simulation was performed using Xepr software. The simulated spectrum (red) uses the following parameters: g = [2.263, 2.086, 2.050].

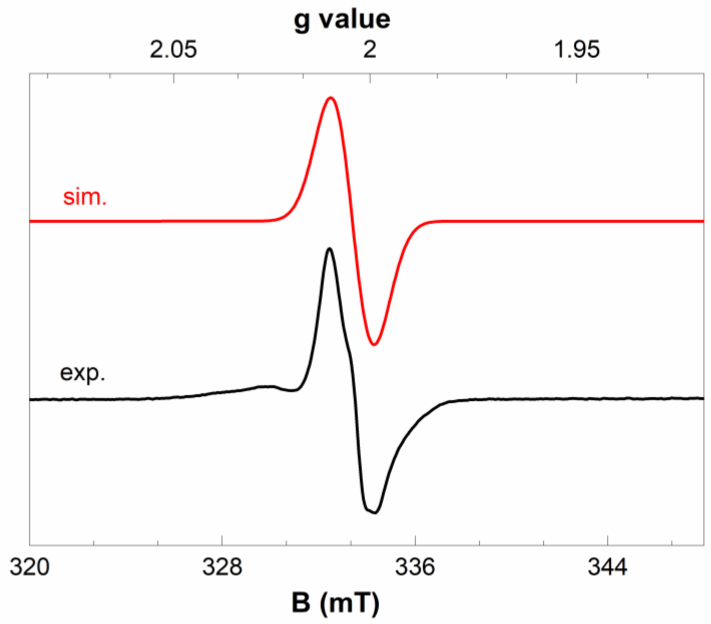


**Figure S52.** X-band EPR spectrum of $KC_8$ reduction of **16** (assigned **18**). Temperature = 100 K, solvent = $Et_2O$/THF, microwave frequency = 9.352807 GHz, power = 0.4743 mW, modulation

amplitude = 1.0 G. modulation frequency = 1 mT/100 kHz. The simulation was performed using Xepr software. The simulated spectrum (red) uses the following parameters: $g_{iso}$ = 2.004.

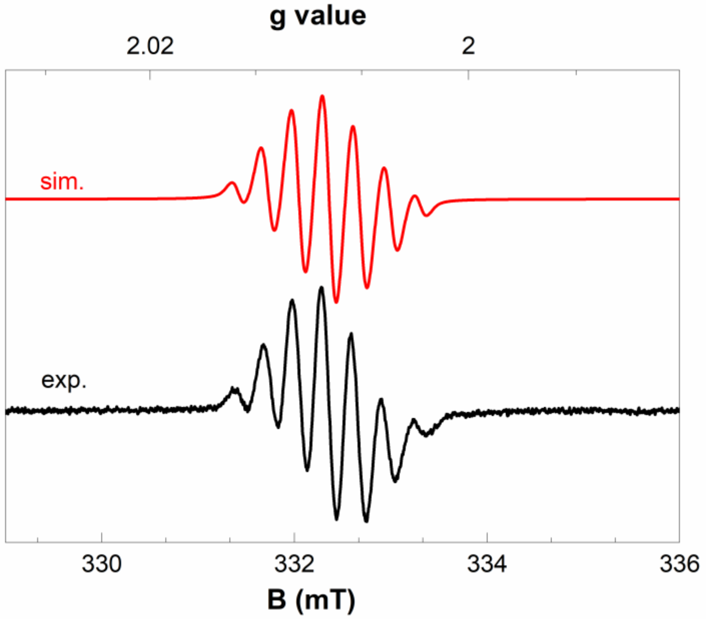


**Figure S53.** X-band EPR spectrum of $KC_8$ reduction of **19** (assigned **20**). Temperature = 295 K, solvent = THF, microwave frequency = 9.34381 GHz, power = 0.4743 mW, modulation amplitude = 0.5 G. modulation frequency = 1 mT/100 kHz. The simulation was performed using Xepr software. The simulated spectrum (red) uses the following parameters: $g_{iso}$ = 2.007, $A_{N,N,H,H}$ = [3.200, 3.200, 2.800, 2.800] G.

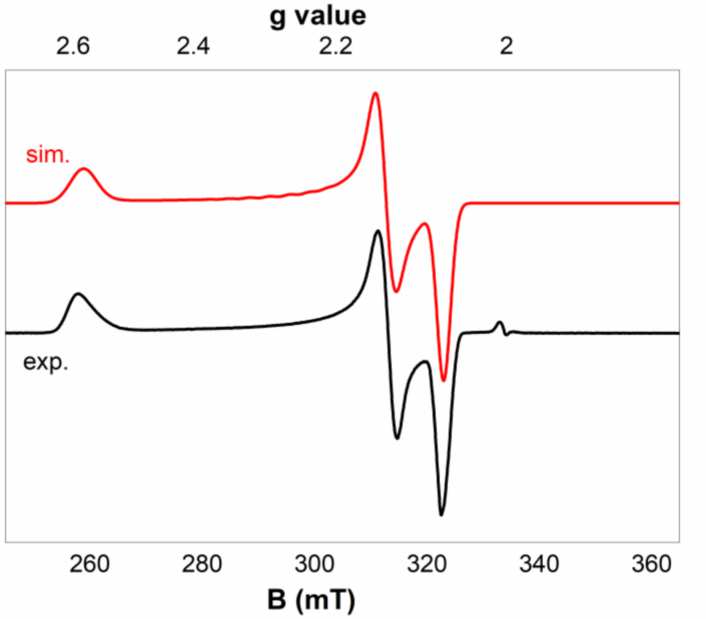

**Figure S54.** X-band EPR spectrum of **21**. Temperature = 30 K, solvent = THF, microwave frequency = 9.353993 GHz, power = 0.47430 mW, modulation amplitude = 4.0 G. modulation frequency = 1 mT/100 kHz. The simulation was performed using Xepr software. The simulated spectrum (red) uses the following parameters: g = [2.585, 2.139, 2.070].

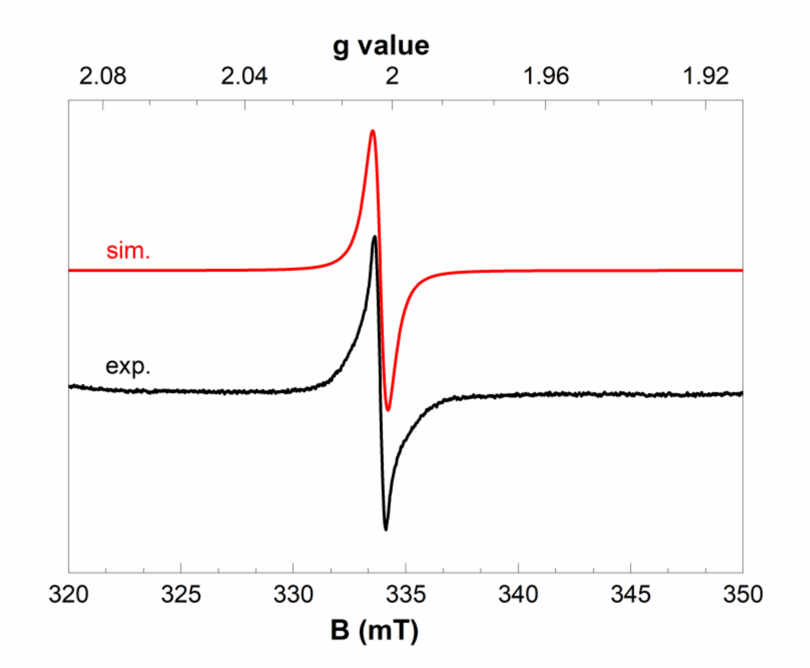


**Figure S55.** X-band EPR spectrum of $KC_8$ reduction of **22** (assigned **23**). Temperature = 5 K, solvent = toluene, microwave frequency = 9.36022 GHz, power = 0.47430 mW, modulation amplitude = 10.0 G. modulation frequency = 1 mT/100 kHz. The simulation was performed using Xepr software. The simulated spectrum (red) uses the following parameters: $g_{iso}$ = 2.003.

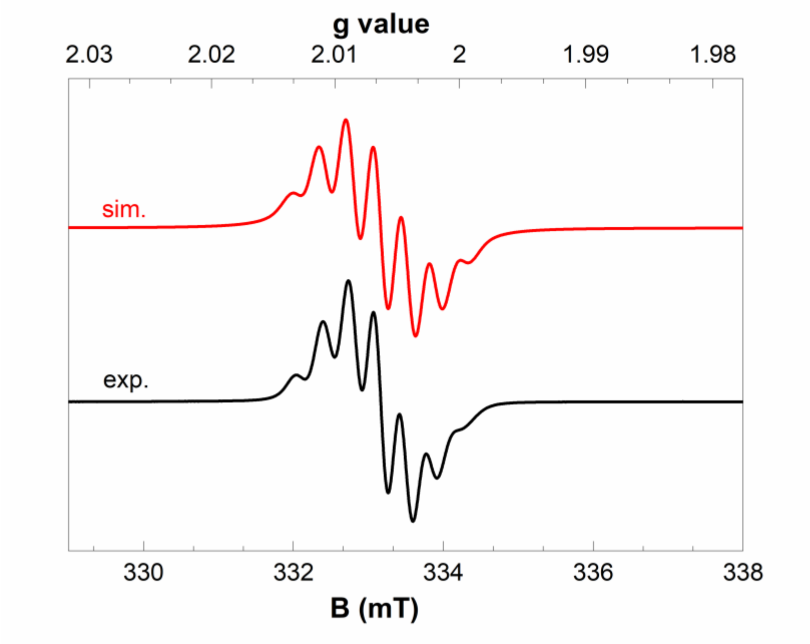

**Figure S56.** X-band EPR spectrum of **26**. Temperature = 295 K, solvent = THF, microwave frequency = 9.355269 GHz, power = 0.01500 mW, modulation amplitude = 1.0 G, modulation frequency = 1 mT/100 kHz. The simulation was performed using Xepr software. The simulated spectrum (red) uses the following parameters: $g_{iso}$ = 2.006, $A_{N,N,H,H}$ = [3.500, 3.500, 3.800, 3.800] G.

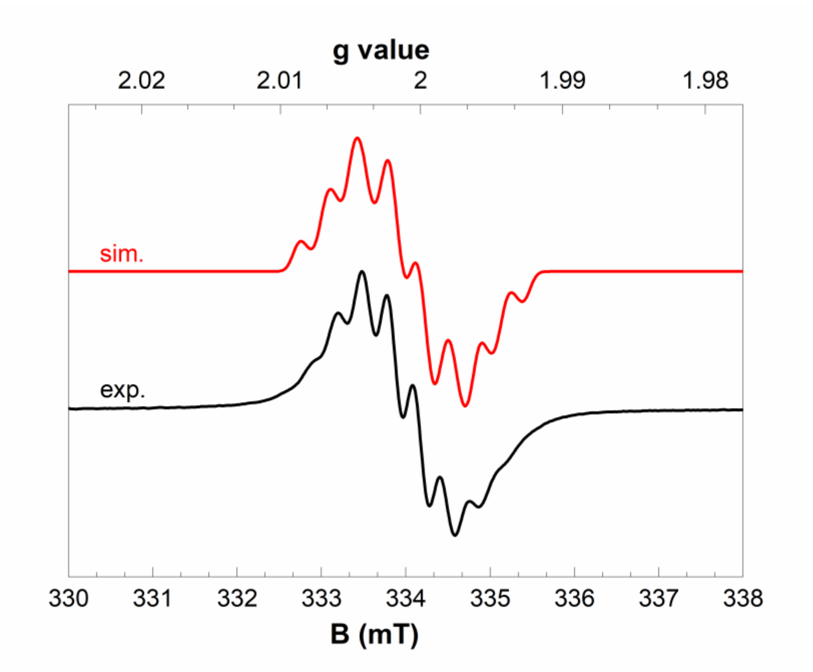


**Figure S57.** X-band EPR spectrum of **28**. Temperature = 295 K, solvent = THF, microwave frequency = 9.354101 GHz, power = 0.06325 mW, modulation amplitude = 1.0 G, modulation frequency = 1 mT/100 kHz. The simulation was performed using Easyspin in MATLAB. The simulated spectrum (red) uses the following parameters: g = 2.001, $A_{N,N,H}$ = [9.0626, 9.0000, 12.1276] MHz.

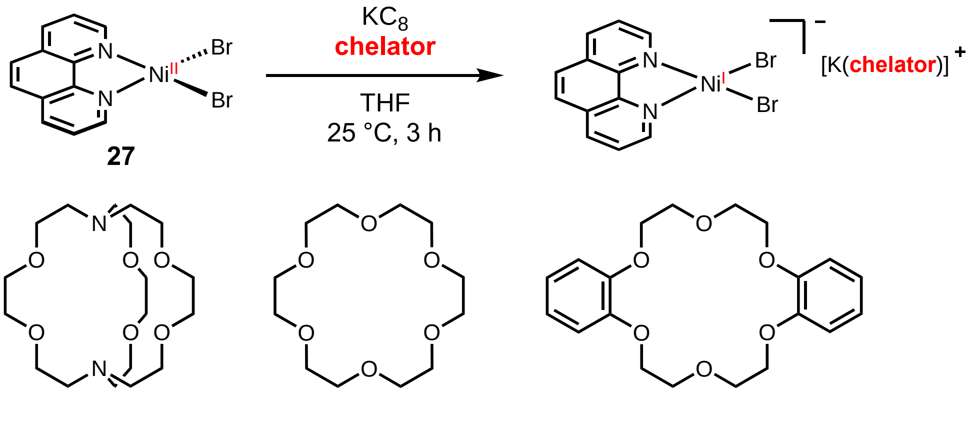


**Effect of chelating agents for stabilization of [(phen)NiBr$_2$]$^-$.** A drastic effect in the stabilization of the reduced $NiBr_2$ was observed when using different chelators for trapping the potassium cation. Cryptand chelators proved ineffective, with little to no paramagnetic signal observed when

Kryptofix 222 was applied to the reduction of (phen)$NiBr_2$. The standard 18-crown-6 ether, used for stabilizing (dtbpy)$Ni(CH_2TMS)_2^-$ and (phen)$Ni(CH_2TMS)_2^-$, displayed some amount of stabilization with the presence of an organic radical but in low intensity. The use of dibenzo-18-crown-6 ether proved to be imperative for stabilizing this species as this produced a signal with a high signal to noise ratio and the observation of hyperfine coupling.

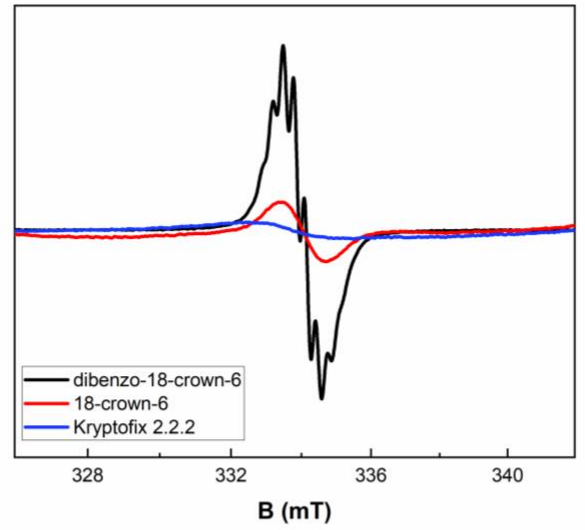


**Figure S58.** Comparison X-band EPR spectra of reduction of (phen)$NiBr_2$ in the presence of different chelators. Temperature = 295 K, solvent = THF.

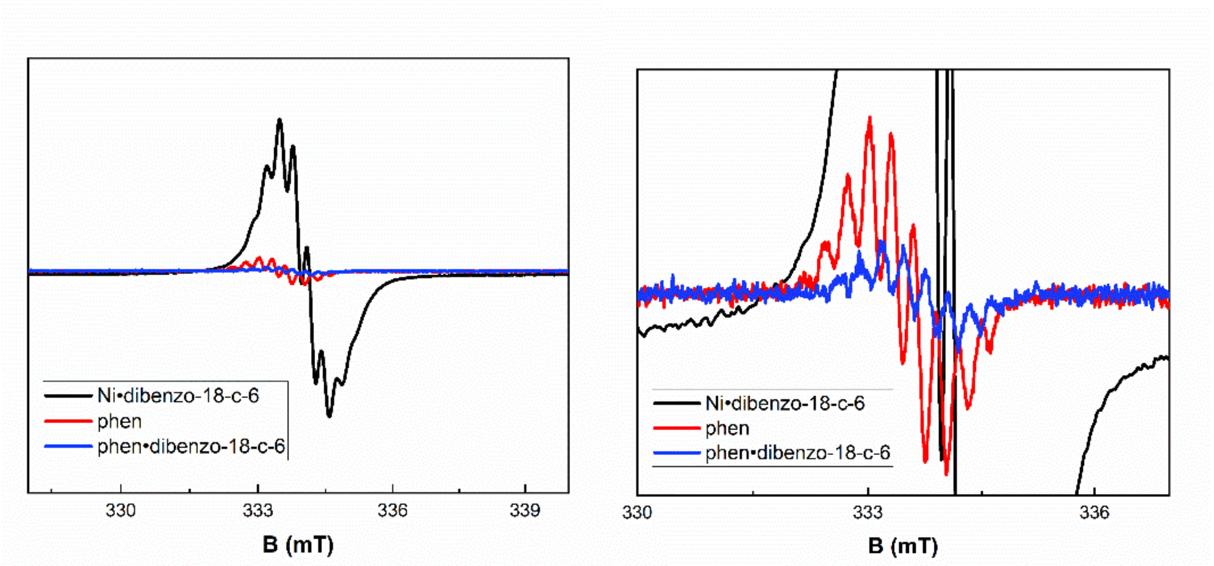


**Figure S59.** Comparison X-band EPR spectra of reduction of (phen)$NiBr_2$ or 1,10-phenanthroline with and without the presence of dibenzo-18-crown-6 ether. Temperature = 295 K, solvent = THF.

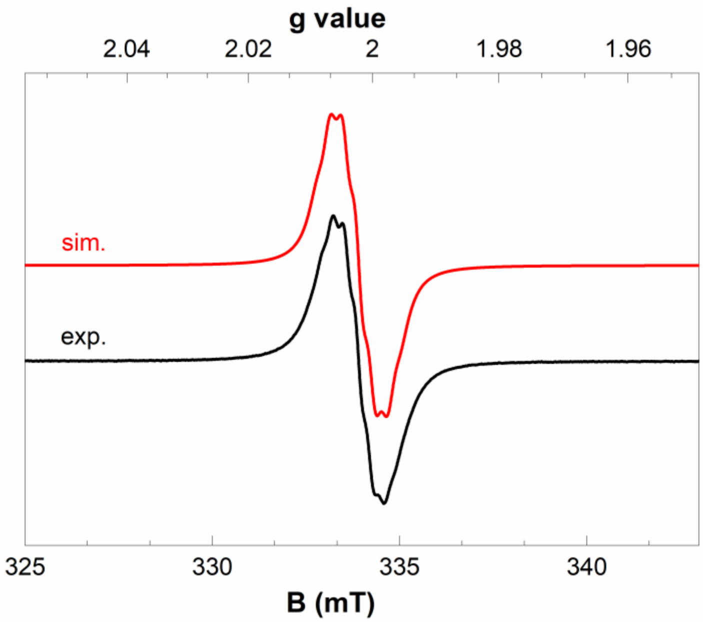


**Figure S60.** X-band EPR spectrum of **30**. Temperature = 295 K, solvent = THF, microwave frequency = 9.356883 GHz, power = 0.04743 mW, modulation amplitude = 1.0 G. modulation frequency = 1 mT/100 kHz. The simulation was performed using Xepr software. The simulated spectrum (red) uses the following parameters: $g_{iso}$ = 2.002, $A_{N,N,H,H}$ = [3.09031, 3.09031, 4.00000, 4.00000] G.

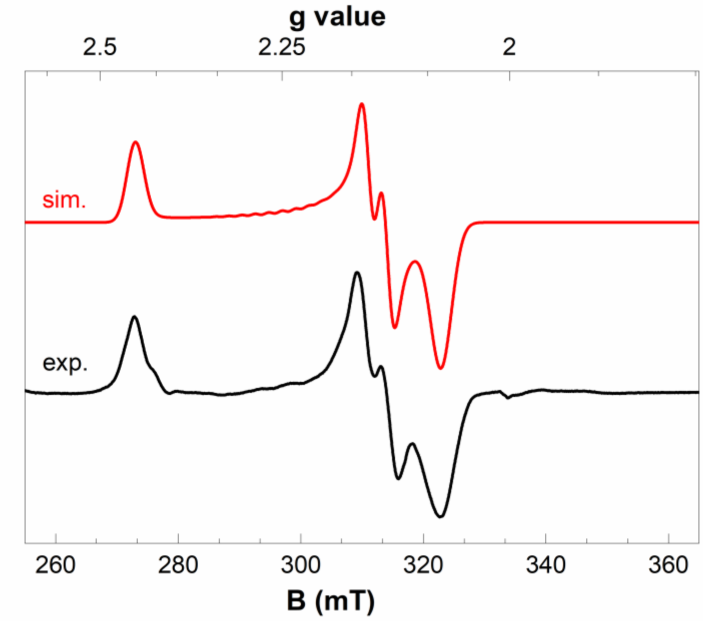


**Figure S61.** X-band EPR spectrum of $KC_8$ reduction of **31** (assigned **32**). Temperature = 30 K, solvent = THF, microwave frequency = 9.350497 GHz, power = 0.4743 mW, modulation amplitude = 10.0 G. modulation frequency = 1 mT/100 kHz. The simulation was performed using Xepr software. The simulated spectrum (red) uses the following parameters: g = [2.448, 2.138, 2.070], $A_{Hx}$ = 10.0 G. $A_{Hy}$ = 30.0 G, $A_{Hz}$ = 0.000 G.

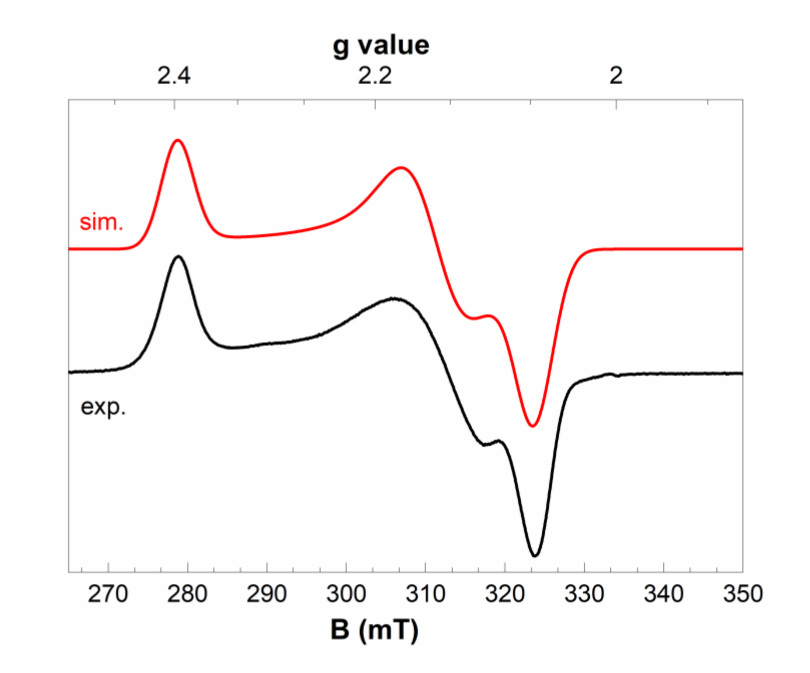


**Figure S62.** X-band EPR spectrum of **S10**. Temperature = 15 K, solvent = THF, microwave frequency = 9.349294 GHz, power = 0.04743 mW, modulation amplitude = 1.0 G, modulation frequency = 1 mT/100 kHz. The simulation was performed using Xepr software. The simulated spectrum (red) uses the following parameters: g = [2.398, 2.148, 2.065].

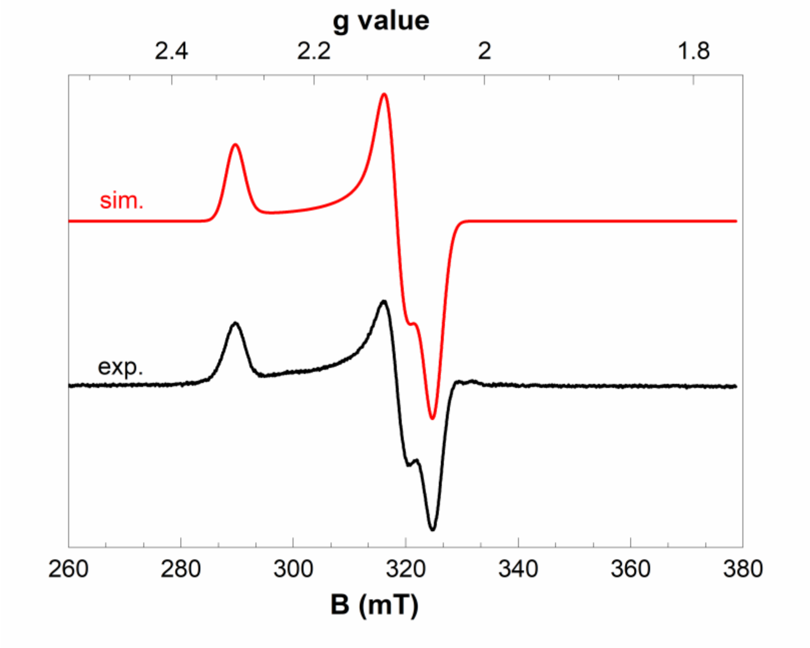


**Figure S63.** X-band EPR spectrum of $KC_8$ reduction of **34** (assigned **35**). Temperature = 30 K, solvent = $Et_2O$, microwave frequency = 9.350579 GHz, power = 0.47430 mW, modulation

amplitude = 10.0 G. modulation frequency = 1 mT/100 kHz. The simulation was performed using Xepr software. The simulated spectrum (red) uses the following parameters: g = [2.308, 2.101, 2.057].

## 7. Single-Crystal Structure Determination

For X-ray data sets collected on a Bruker D8 VENTURE (**S2**, **14**, **21**, **26**, **32**): The structures were solved using a dual-space method and standard difference map techniques and were refined by full-matrix least-squares procedures on $F^2$ with SHELXTL (Version 2018/3).[31] All hydrogen atoms were placed in calculated positions and refined with a riding model [$U_{iso}$(H) = 1.2–1.5$U_{eq}$(C)].

For X-ray data sets collected on a Bruker D8 SMART APEXII (**12**, **19**, **22**, **30'**): Data sets were processed with the INTEGRATE program of the APEX3 software[32,33] for reduction and cell refinement. Multi-scan absorption corrections were applied by the SCALE program for the area detector. All structures were solved by intrinsic phasing methods (SHELXT)[31b] and the structure models were completed and refined using the full-matrix least-square methods on $F^2$ (SHELXL).[34,35] Non-hydrogen atoms in the structures were refined with anisotropic displacement parameters, and hydrogen atoms on carbons were placed in idealized positions (C—H = 0.95-1.00 Å) and included as riding with $U_{iso}$ (H) = 1.2 or 1.5 $U_{eq}$(non-H).

All structures were validated by the IUCr checkCIF10 for consistency and integrity. Crystallographic information files (CIFs), including the HKL and RES data, are deposited in the CCDC with nos. 2271481—9. Selected crystallographic parameters are listed in Table S2. The molecular graphics (Fig. S64-S72) in ORTEP style were drawn by the Olex2 program.[36] Atom labels were standardized between structures in the main text for clarity, but the atom labels used in the CIFs are the ones shown below for ease of reference to the original data.

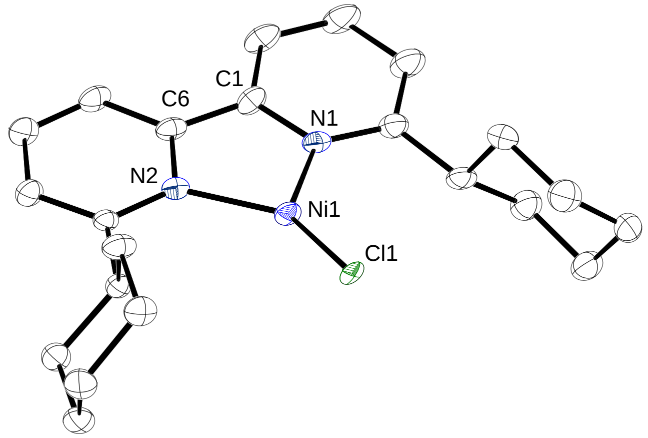


**Figure S64.** Molecular structure of ($^{Cy}$bpy)NiCl **12**. The thermal ellipsoids are shown at 50% probability. Hydrogen atoms have been omitted for clarity. Selected bond angles (°) and distances (Å): Ni1–N1 1.954(4), Ni1–N2 1.950(4), Ni1–Cl1 2.1741(12), C1–C6 1.466(7), C1–N1 1.361(6), C6–N2 1.359(6), C6–C1–N1 114.0(4), C1–C6–N2 114.3(4).

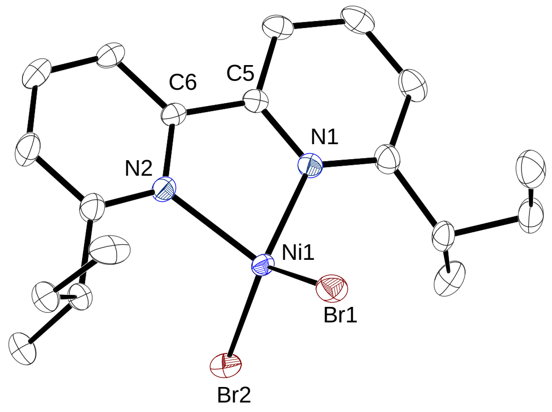


**Figure S65.** Molecular structure of ($^{sBu}$bpy)$NiBr_2$ **S2**. The thermal ellipsoids are shown at 50% probability. Hydrogen atoms, disorder, and $Et_2O$ have been omitted for clarity. Selected bond angles (°) and distances (Å): Ni1–N1 1.997(2), Ni1–N2 2.002(2), Ni1–Br1 2.3665(4), Ni1–Br2 2.3323(4), C5–C6 1.479(3), C5–N1 1.351(3), C6–N2 1.353(3), C6–C5–N1 115.52(19), C5–C6–N2 115.26(19).

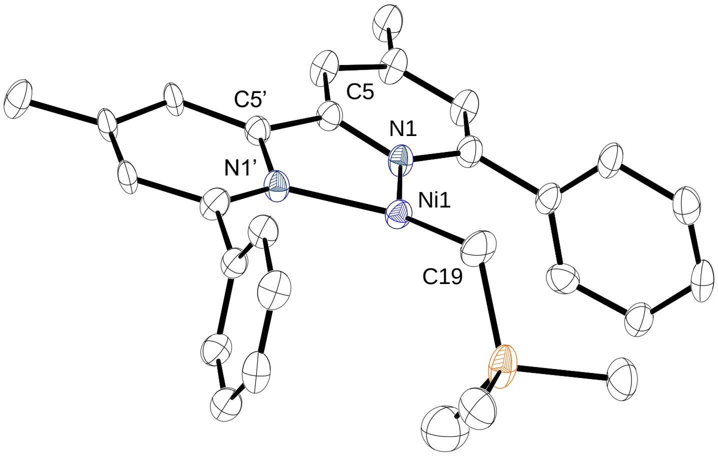


**Figure S66.** Molecular structure of ($^{Mes}$dtbpy)Ni($CH_2$TMS) **14**. The thermal ellipsoids are shown at 50% probability. Magnesium bromide impurity and Ni($CH_2$TMS) disorder are present. Hydrogen atoms have been omitted and *t*Bu- and Mes-groups truncated for clarity. Selected bond angles (°) and distances (Å): Ni1–N1 1.985(3), Ni1–N1' 1.985(3), Ni1–C19 1.957(18), C5–C5' 1.484(3), C5–N1 1.364(2), C5'–N1' 1.364(2), C5'–C5–N1 113.15(9), C5–C5'–N1' 113.15(9).

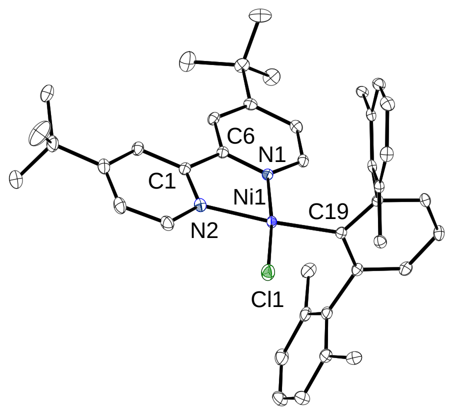


**Figure S67.** Molecular structure of (dtbpy)Ni(Dipp*)Cl **19** (one of two crystographically independent molecules). The thermal ellipsoids are shown at 30% probability. Hydrogen atoms have been omitted and *i*Pr-groups truncated for clarity. Selected bond angles (°) and distances (Å): Ni1–N1 1.926(2), Ni1–N2 1.975(2), Ni1–Cl1 2.1551(8), Ni1–C19 1.897(2), C1–C6 1.472(3), C6–N1 1.364(3), C1–N2 1.348(3), C1–C6–N1 113.8(2), C6–C1–N2 113.3(2).

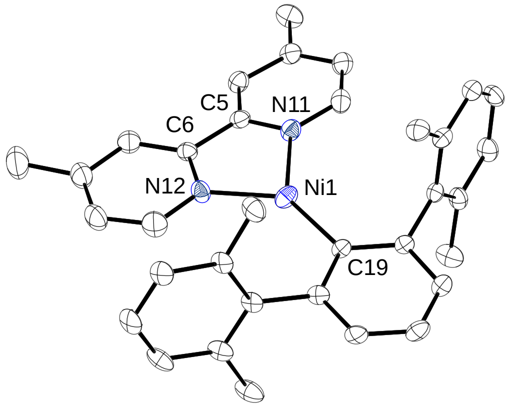


**Figure S68.** Molecular structure of (dtbpy)Ni(Dipp*) **21** (one of two crystographically independent molecules). The thermal ellipsoids are shown at 50% probability. Hydrogen atoms have been omitted and *t*Bu- and *i*Pr-groups truncated for clarity. Selected bond angles (°) and distances (Å): Ni1–N11 1.977(3), Ni1–N12 1.989(3), Ni1–C19 1.979(3), C5–C6 1.470(5), C5–N11 1.354(5), C6–N12 1.342(5), C6–C5–N11 113.4(3), C5–C6–N12 113.9(3).

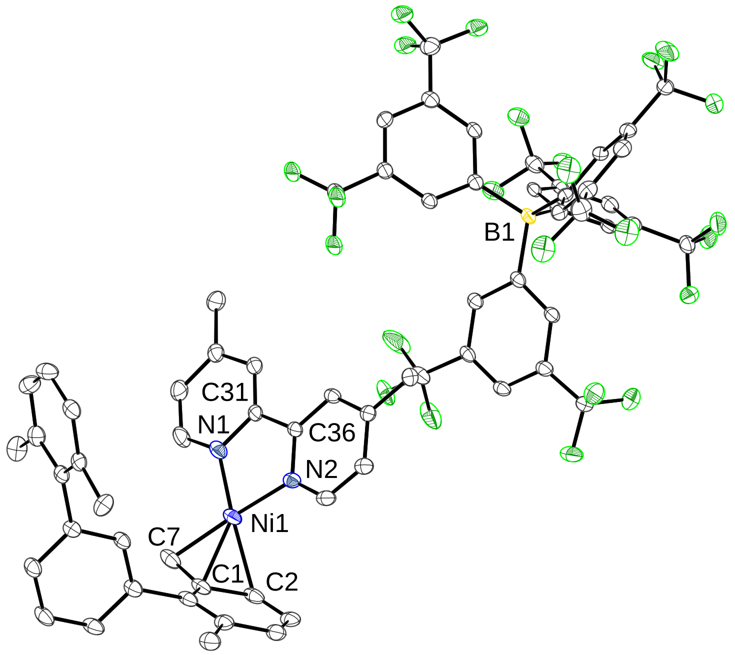


**Figure S69.** Molecular structure of [(dtbpy)Ni(η-3 Dipp*)][BAr$^{F}{}_{24}$] **22**. The thermal ellipsoids are shown at 30% probability. Hydrogen atoms have been omitted and *t*Bu- and *i*Pr-groups truncated for clarity. Selected bond angles (°) and distances (Å): Ni1–N1 1.920(4), Ni1–N2 1.955(4), Ni1–C7 2.000(5), Ni1–C1 2.009(4), Ni1–C2 2.099(5) C31–C36 1.472(6), C31–N1 1.370(5), C36–N2 1.353(5), C36–C31–N1 113.8(4), C31–C36–N2 114.2(3), C7–C1–C2 114.4(4).

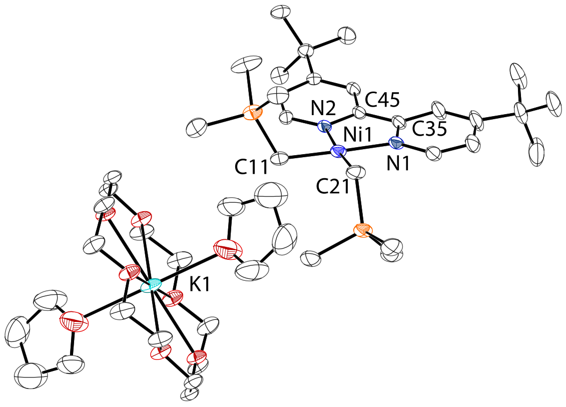


Figure **S70**. Molecular structure of ([K(18-crown-6)][(dtbpy)Ni($CH_2$TMS)$_2$] **26**. The thermal ellipsoids are shown at 50% probability. Though there appears to be 2 K units vs 1 Ni unit in structure, that is an artifact or symmetry as both K units are on inversion centers and only contribute ½ of a cation each to the asymmetric unit. Overall, the K:Ni ratio is 1:1. Hydrogen atoms and one K(18-crown-6) omitted for clarity. Selected bond angles (°) and distances (Å): Ni1–N1 1.955 (2), Ni1–N2 1.958(2), Ni1–C11 1.955(3), Ni1–C21 1.953(3), C35–C45 1.414(4), C35–N1 1.387(3), C45–N2 1.383(4), C45–C35–N1 114.3(2), C35–C45–N2 114.4(2).

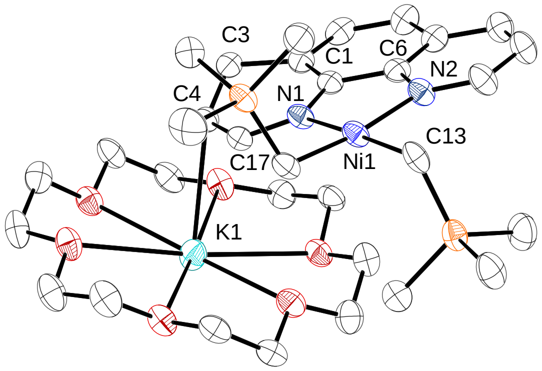


**Figure S71.** Molecular structure of ([K(18-crown-6)]$_2$[(phen)Ni(CH$_2$TMS)$_2$]$_2$ **30'**. The thermal ellipsoids are shown at 50% probability. Compound exists as dimer but only monomer shown, hydrogen atoms omitted, and Et$_2$O-coordination omitted for clarity. Selected bond angles (°) and distances (Å): Ni1–N1 1.956(3), Ni1–N2 1.971(3), Ni1–C17 1.949(4), Ni1–C13 1.960(4), C1–C6 1.423(5), C4–C3 1.500(5), C4–K1 3.105(4), C1–N1 1.358(4), C6–N2 1.383(5), C6–C1–N1 116.1(3), C1–C6–N2 114.3(3).

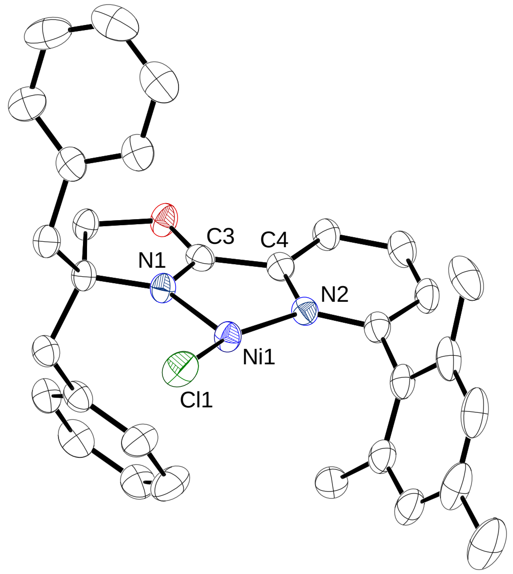


**Figure S72.** Molecular structure of (6-Mes-$^{dBn}$pyrox)NiCl **32**. The thermal ellipsoids are shown at 50% probability. Hydrogen atoms have been omitted for clarity. Selected bond angles (°) and distances (Å): Ni1–N1 1.978(4), Ni1–N2 1.960(4), Ni1–Cl1 2.1443(14), C3–C4 1.459(7), C3–N1 1.288(6), C4–N2 1.362(7), C4–C3–N1 119.3(5), C3–C4–N2 111.7(4).

**Table S2.** Crystallographic data.

| Compound name | **12** | **S2** | **14** | **19** |
|---|---|---|---|---|
| **Lab code** | 22tnd6g | sBuNiBr2 | 201_287 | 21tnd11h |
| **CCDC no.** | 2271481 | 2271486 | 2271482 | 2271483 |
| **Formula by X-ray** | $C_{22}H_{28}ClN_2Ni$ | $C_{20}H_{29}Br_2N_2NiO_{0.5}$ | $C_{47.60}H_{73.89}Br_{0.16}Mg_{0.10}N_2Ni_{0.90}O_2Si_{0.90}$ | $C_{56.75}H_{82}ClN_2Ni$ |
| **Formula weight** | 414.62 | 523.98 | 798.98 | 886.40 |
| **Crystal size (mm)** | 0.02 x 0.04 x 0.34 | 0.31 x 0.11 x 0.06 | 0.21 x 0.26 x 0.32 | 0.09 x 0.33 x 0.37 |
| **Crystal system** | Orthorhombic | Monoclinic | Orthorhombic | Triclinic |
| **Space group (no.)** | $Pca2_1$ | $P2_1/n$ | $Cmc2_1$ | P-1 |
| ***a*** **(Å)** | 20.710(3) | 10.5267(5) | 14.2296(4) | 13.4186(11) |
| ***b*** **(Å)** | 9.1728(11) | 16.2089(7) | 29.2007(8) | 19.5707(15) |
| ***c*** **(Å)** | 10.4641(13) | 13.1463(5) | 11.4945(4) | 22.1414(17) |
| $\alpha$ **(°)** | 90 | 90 | 90 | 103.999(3) |
| $\beta$ **(°)** | 90 | 100.5480(10) | 90 | 103.125(3) |
| $\gamma$ **(°)** | 90 | 90 | 90 | 103.282(3) |
| $V$ **(Å$^3$)** | 1987.9(4) | 2205.20(16) | 4776.1(3) | 5239.2(7) |
| ***Z, Z'*** | 4, 1 | 4, 1 | 4, 0.5 | 4, 2 |
| $D_c$ **(g cm$^{-3}$)** | 1.385 | 1.578 | 1.111 | 1.124 |
| ***F*****(000)** | 876 | 1060 | 1735 | 1926 |
| $\mu$ **(mm$^{-1}$)** | 1.118 | 4.512 | 0.557 | 0.457 |
| **Total reflections** | 27140 | 70449 | 56578 | 95191 |
| **Unique reflections** | 4378 | 7781 | 5893 | 21525 |
| $R_{int}$ | 0.0621 | 0.0653 | 0.0465 | 0.0503 |
| $R_1$$^a$ **[$I > 2\sigma(I)$]** | 0.0431 | 0.0398 | 0.0439 | 0.0551 |
| $wR_2$$^b$ **(all data)** | 0.0940 | 0.0990 | 0.1293 | 0.1282 |
| **GOF (all data)** | 1.063 | 1.025 | 1.084 | 1.088 |
| **Flack parameter** | 0.134(10) | - | 0.52(5) | - |
| **Restraints** | 1 | 352 | 119 | 116 |
| **Diffractometer** | Bruker D8 SMART APEXII | Bruker D8 VENTURE | Bruker D8 VENTURE | Bruker D8 SMART APEXII |

$^aR_1 = \Sigma||F_o|-|F_c||/\Sigma|F_o|$; $^bwR_2=\{\Sigma[w(F_o^2-F_c^2)^2]/\Sigma[w(F_o^2)^2]\}^{1/2}$.

**Table S2 (cont.).** Crystallographic data.

| Compound name | **21** | **22** | **26** | **30'** |
|---|---|---|---|---|
| **Lab code** | niterphen | 22tnd3h | nins2 | 23tnd3h |
| **CCDC no.** | 2271487 | 2271489 | 2271488 | 2271484 |
| **Formula by X-ray** | $C_{48}H_{61}N_2Ni$ | $C_{81.57}H_{76.14}BCl_{3.14}F_{23.99}N_2Ni$ | $C_{46}H_{86}KN_2NiO_8Si_2$ | $C_{76}H_{138}K_2N_4Ni_2O_{15}Si_4$ |
| **Formula weight** | 724.69 | 1721.25 | 949.15 | 1655.88 |
| **Crystal size (mm)** | 0.15 x 0.07 x 0.02 | 0.11 x 0.25 x 0.45 | 0.24 x 0.14 x 0.06 | 0.10 x 0.21 x 0.36 |
| **Crystal system** | Triclinic | Monoclinic | Triclinic | Orthorhombic |
| **Space group (no.)** | P-1 | $P\ 1\ 2_1/n\ 1$ | P-1 | Pbcn |
| ***a*** **(Å)** | 14.1824(4) | 14.5135(5) | 11.0102(4) | 17.0071(10) |
| ***b*** **(Å)** | 14.3424(4) | 31.6289(10) | 13.6066(4) | 20.7644(13) |
| ***c*** **(Å)** | 23.6619(6) | 18.3661(7) | 20.0564(7) | 25.4726(15) |
| $\alpha$ **(Å)** | 76.612(2) | 90 | 76.424(2) | 90 |
| $\beta$ **(Å)** | 81.635(2) | 98.4080(13) | 77.185(2) | 90 |
| $\gamma$ **(Å)** | 67.752(2) | 90 | 70.192(2) | 90 |
| $V$ **(Å$^3$)** | 4324.7(2) | 8340.3(5) | 2714.47(16) | 8995.5(9) |
| ***Z*** | 4, 2 | 4, 1 | 2, 1 | 4, 0.5 |
| $D_c$ **(g cm$^{-3}$)** | 1.113 | 1.371 | 1.161 | 1.223 |
| ***F*****(000)** | 1564 | 3528 | 1030 | 3568 |
| $\mu$ **(mm$^{-1}$)** | 0.875 | 0.430 | 1.994 | 0.623 |
| **Total reflections** | 97563 | 43340 | 73619 | 89323 |
| **Unique reflections** | 17055 | 16828 | 10960 | 8029 |
| $R_{int}$ | 0.1537 | 0.0389 | 0.0492 | 0.0967 |
| $R_1$$^a$ **[$I > 2\sigma(I)$]** | 0.0927 | 0.0836 | 0.0592 | 0.0630 |
| $wR_2$$^b$ **(all data)** | 0.2525 | 0.2305 | 0.1667 | 0.1623 |
| **GOF (all data)** | 1.038 | 1.016 | 1.043 | 1.085 |
| **Flack parameter** | - | - | - | - |

| | | | | |
|---|---|---|---|---|
| **Restraints** | 96 | 135 | 586 | 42 |
| **Diffractometer** | Bruker D8 VENTURE | Bruker D8 SMART APEXII | Bruker D8 VENTURE | Bruker D8 SMART APEXII |

**Table S2 (cont.).** Crystallographic data.

| **Compound name** | **32** |
|---|---|
| **Lab code** | pyoxnicl |
| **CCDC no.** | 2271485 |
| **Formula by X-ray** | $C_{31}H_{30}ClN_2NiO$ |
| **Formula weight** | 540.73 |
| **Crystal size (mm)** | 0.34 x 0.08 x 0.02 |
| **Crystal system** | Orthorhombic |
| **Space group (no.)** | Aba2 |
| ***a* (Å)** | 18.9218(4) |
| ***b* (Å)** | 19.9779(5) |
| ***c* (Å)** | 14.0511(3) |
| **$\alpha$ (Å)** | 90 |
| **β (Å)** | 90 |
| **γ (Å)** | 90 |
| **$V$ (Å$^3$)** | 5311.6(2) |
| ***Z, Z'*** | 8, 1 |
| **$D_c$ (g cm$^{-3}$)** | 1.352 |
| ***F*(000)** | 2264 |
| **μ (mm$^{-1}$)** | 2.177 |
| **Total reflections** | 59248 |
| **Unique reflections** | 5012 |
| **$R_{int}$** | 0.0609 |
| **$R_1^a$ [$I > 2\sigma(I)$]** | 0.0596 |
| **$wR_2^b$ (all data)** | 0.1623 |
| **GOF (all data)** | 1.106 |
| **Flack parameter** | 0.00(2) |
| **Restraints** | 1 |
| **Diffractometer** | Bruker D8 VENTURE |

## 8. DFT Calculations

We performed density functional theory (DFT) calculations on the electronic structures of radical nickel complexes described in this study. Due to the potential influence of the DFT method and basis set on computational results, we evaluated various basis sets for geometry optimization using crystal structures of **6**[13], **7**[9], **9**[15], **10**[15], **12**, and **21** as benchmarks.

First, we performed geometry optimization on (neocuproine)Ni(Trip) (**7**)[9] (Trip = 2,4,6-triisopropylphenyl) using its crystal structure as a starting point. The resulting geometry was found to be highly sensitive to the functional and the basis set. The combination of B3LYP-D3 functional[37] with def2-TZVP basis set[38] distorted the geometry from trigonal planar to trigonal pyramidal. Switching to B97D[37c] and BP86[39] functionals provided better agreement on bond lengths but caused the geometry to contort into a T-shape. Using B3LYP/m6-31g* provided reasonable agreement in geometry with the experimental structure, but led to an overestimation in bond lengths. Changing the basis set on Ni from m6-31g* to SDD resulted in a T-shaped geometry. Screening different functionals such as BP86, ωB97X-D3[40] and TPSSh[41] while keeping

the basis set as m6-31g* did not noticeably improve the results. Among the tested basis sets and functions, only the combination of (U)B3LYP//m6-31g* provided reasonable agreement with the experimental structure for [Ni(I)-aryl] complexes.[21, 42] A similar trend was observed for (dtbpy)Ni(Dipp*) (**21**) (Table S4).

**Table S3.** Comparison of DFT computed geometry and X-ray crystal structure on (neocuproine)Ni(Trip) (**7**).

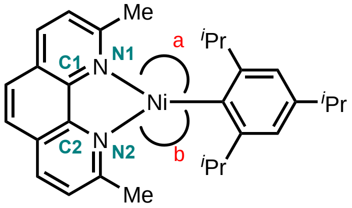


**7**

| Method | N(1)−C(1) (Å) | N(2)−C(2) (Å) | C(1)−C(2) (Å) | Ni-C(3) (Å) | Δ(α−β) (°) | Dihedral angle(°) |
|---|---|---|---|---|---|---|
| Crystal structure | 1.367 | 1.380 | 1.424 | 1.959 | 4.9 | 168.9 |
| B3LYP/def2-TZVP | 2.012 | 2.021 | 1.431 | 1.993 | 2.9 | 137.3 |
| BP86/def2-TZVP | 1.376 | 1.377 | 1.431 | 1.939 | 29.2 | 159.2 |
| B97-D/def2-TZVP | 1.373 | 1.374 | 1.432 | 1.94 | 32.1 | 155.6 |
| **B3LYP/m6-31g*** | **1.418** | **1.408** | **1.476** | **2.122** | **3.9** | **174.1** |
| B3LYP-D3/m6-31g* | 1.416 | 1.408 | 1.476 | 2.119 | 9.1 | 174.5 |
| B3LYP/SDD-6-31g* | 1.387 | 1.400 | 1.417 | 1.998 | 76.8 | 167.0 |
| BP86/m6-31g* | 1.422 | 1.418 | 1.483 | 2.211 | 7.3 | 172.3 |
| ωB97X-D3/m6-31g* | 1.393 | 1.394 | 1.497 | 2.076 | 0.4 | 177.7 |
| TPSSh/m6-31g* | 1.421 | 1.411 | 1.471 | 2.106 | 7.2 | 173.7 |

**Table S4.** Comparison of DFT computed geometry and X-ray crystal structure on (dtbpy)Ni(Dipp*) (**21**).

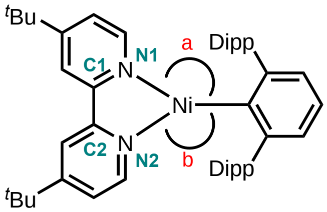


**21**

| Method | N(1)−C(1) (Å) | N(2)−C(2) (Å) | C(1)−C(2) (Å) | Ni-C(3) (Å) | Δ(α−β) (°) | Dihedral angle (°) |
|---|---|---|---|---|---|---|

| | | | | | | |
|---|---|---|---|---|---|---|
| Crystal structure | 1.343 | 1.353 | 1.472 | 1.970 | 13.2 | 158.8 |
| **B3LYP/m6-31g*** | **1.397** | **1.408** | **1.528** | **2.132** | **20** | **167.6** |

When optimizing the structures for [Ni(I)Cl] and $[Ni(I)R_2]^-$, the B3LYP/m6-31g* method performed poorly in reproducing the experimental geometries. The structures of ($^{Cy}$bpy)NiCl (**12**) and ($^{sBu}$phen)NiBr (**6**) deviated from trigonal planar to trigonal pyramidal, while both $[(^{tBu}pyrox)Ni(Dipp)_2]^-$ (**10**) and $[(^{tBu}pyrox)Ni(CH_2TMS)_2]^-$ contorted from square planar to tetrahedral. Conversely, B3LYP-D3/def2-TZVPP yielded the best agreement in geometry and bond lengths for all four compounds (Table S5-S6).

**Table S5.** Comparison of DFT computed geometry and X-ray crystal structure on ($^{Cy}$bpy)NiCl (**12**) and ($^{sBu}$phen)NiBr (**6**)

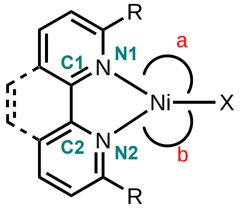


| Method | N(1)−C(1) (Å) | N(2)−C(2) (Å) | C(1)−C(2) (Å) | Ni-X (Å) | Δ(α−β) (°) | Dihedral angle (°) |
|---|---|---|---|---|---|---|
| ($^{Cy}$bpy)NiCl Crystal structure | 1.360 | 1.360 | 1.466 | 2.174 | 2.6 | 170.8 |
| **B3LYP-D3/def2-TZVPP** | **1.351** | **1.353** | **1.478** | **2.172** | **2.0** | **179.0** |
| B3LYP/m6-31g* | 1.409 | 1.408 | 1.519 | 2.234 | 1.1 | 152.5 |
| ($^{sBu}$phen)NiBr Crystal structure | 1.364 | 1.361 | 1.426 | 2.288 | 2.0 | 173.7 |
| **B3LYP-D3/def2-TZVPP** | **1.354** | **1.355** | **1.431** | **2.302** | **1.2** | **172.6** |

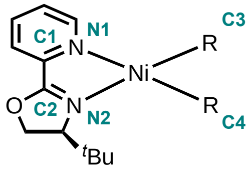


**Table S6.** Comparison of DFT computed geometry and X-ray crystal structure on $[(^{tBu}pyrox)Ni(Dipp)_2]^-$ (**10**) and $[(^{tBu}pyrox)Ni(CH_2TMS)_2]^-$

| Method | Nox−Cox (N(1)−C(1) Å) | Cpyr−Npyr (C(2)−N(2) Å) | Cox−Cpyr (C(1)−C(2) Å) | Ni−C(3) (Å) | Ni−C(4) (Å) | Dihedral angle (°) |
|---|---|---|---|---|---|---|
| $[(^{tBu}pyrox)Ni(Dipp)_2]^-$ Crystal structure | 1.317(12) | 1.378(12) | 1.423(14) | 1.916(9) | 1.940(9) | 161.1 |
| B3LYP/m6-31g* | 1.332 | 1.462 | 1.492 | 2.167 | 2.113 | 137.7 |
| B3LYP/def2-TZVP | 1.321 | 1.398 | 1.398 | 1.988 | 1.96 | 157.2 |
| **B3LYP-D3/def2-TZVPP** | **1.322** | **1.397** | **1.405** | **1.921** | **1.965** | **159.3** |
| $[(^{tBu}pyrox)Ni(CH_2TMS)_2]^-$ Crystal structure | 1.311(7) | 1.402(8) | 1.393(8) | 1.934(6) | 1.952(6) | 170.4 |
| B3LYP/m-6-31g* | 1.368 | 1.457 | 1.452 | 2.105 | 2.121 | 153.9 |
| B3LYP/def2-TZVP | 1.319 | 1.397 | 1.398 | 1.972 | 1.974 | 164.4 |
| **B3LYP-D3/def2-TZVPP** | **1.322** | **1.398** | **1.404** | **1.964** | **1.966** | **172.4** |

In summary, B3LYP-D3/def2-TZVPP was successful in reproducing the experimental structures of $[Ni(I)R_2]^-$ and [Ni(I)X] complexes, but performed poorly in computing the geometry of [Ni(I)–aryl] compounds. The use of B3LYP/m6-31g* was necessary for proper optimization of geometry for [Ni(I)-aryl] to match reported structures. The four-coordinate $[NiX_2]^-$, $[NiArX]^-$, $[NiR_2]^-$ and [Ni(allyl)] complexes were computed to be square planar with highly delocalized spin density on the ligands and elongated C=N bonds (Table S7). The three-coordinate [Ni(I)X] and [Ni(I)-aryl] complexes were computed as being trigonal planar with highly localized spin density on the Ni centers. The C=N bonds on the ligands were computed to be shorter compared to those in the computed four-coordinate molecules. It should be noted that a fair comparison of bond length and spin density can only be achieved if the compounds are computed using the same method.

**Table S7.** Spin-density of Nickel Radical Complexes Derived from DFT Calculations

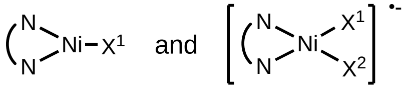


| Ligand | $X^1$ | $X^2$ | Bond length $(C{=}N)_{ligand}$ (Å) | Spin density on Ni | Basis set |
|---|---|---|---|---|---|
| 3-coordinate | | | | | |
| bpy | Cl | | 1.356, 1.355 | 0.997 | B3LYP-D3/def2-TZVPP |
| bpy | Ph | | 1.414, 1.423 | 1.489 | B3LYP/m6-31g* |
| phen | Cl | | 1.360,1.360 | 0.993 | B3LYP-D3/def2-TZVPP |
| pyrox | Cl | | 1.282, 1.354 | 0.996 | B3LYP-D3/def2-TZVPP |
| 4-coordinate | | | | | |
| bpy | Cl | Cl | 1.387, 1.387 | 0.017 | B3LYP-D3/def2-TZVPP |
| bpy | h$^3$-allyl | | 1.428,1.436 | 0.107 | B3LYP/m6-31g* |
| phen | Cl | Cl | 1.364, 1.375 | 0.018 | B3LYP-D3/def2-TZVPP |
| phen | Ph | Br | 1.385, 1.393 | 0.025 | B3LYP-D3/def2-TZVPP |
| phen | Ph | Ph | 1.390, 1.389 | 0.030 | B3LYP-D3/def2-TZVPP |

**Input File for Energy and Mulliken Spin Density Calculation of $^{Cy}$bpyNiCl (12)**

```
# 12
! UKS B3LYP D3 RIJCOSX SlowConv TightSCF def2-SVP def2/j Normalprint UCO OPT

%basis NewGTO 28 "def2-TZVPP" end
NewGTO 7 "def2-TZVPP" end
NewGTO 17 "def2-TZVPP" end
NewAuxGTO 28 "def2/j" end
NewAuxGTO 7 "def2/j" end
NewAuxGTO 17 "def2/j" end
end

%scf
MaxIter 1500
TolE 1E-7
TolErr 1E-6
end

* xyz 0 2
Coordination from X-ray structure*

%plots format cube
dim1 100 dim2 100 dim3 100
SpinDens("12.cube");
end
```

**Input File for Energy and Mulliken Spin Density Calculation of $^{Mes}$bpyNi($CH_2$TMS) (14)**

```
#14
! UKS B3LYP D3 RIJCOSX SlowConv TightSCF def2-SVP def2/j Normalprint UCO OPT FREQ

%basis NewGTO 28 "def2-TZVPP" end
NewGTO 7 "def2-TZVPP" end
NewGTO 14 "def2-TZVPP" end
NewAuxGTO 28 "def2/j" end
NewAuxGTO 7 "def2/j" end
NewAuxGTO 14 "def2/j" end
end

%scf
MaxIter 1500
TolE 1E-7
TolErr 1E-6
end

* xyz 0 2
Coordination from X-ray structure, magnesium bromide impurity omitted*

%plots format cube
dim1 100 dim2 100 dim3 100
SpinDens("14.cube");
end
```

**Input File for Energy and Mulliken Spin Density Calculation of (dtbpy)Ni(Dipp)•THF (16)**

```
#18
! UKS B3LYP D3 RIJCOSX SlowConv TightSCF def2-SVP def2/j Normalprint UCO OPT FREQ

%basis NewGTO 28 "def2-TZVPP" end
NewGTO 7 "def2-TZVPP" end
NewGTO 17 "def2-TZVPP" end
NewAuxGTO 28 "def2/j" end
NewAuxGTO 7 "def2/j" end
NewAuxGTO 17 "def2/j" end
end

%scf
MaxIter 1500
TolE 1E-7
TolErr 1E-6
end

* xyz 0 2
Ni      7.1931230000      9.9717910000      8.6360510000
O       8.0267880000      9.5751270000     10.4399360000
```

| | | | |
|---|---|---|---|
| N | 6.2811270000 | 10.1557290000 | 6.9708470000 |
| N | 7.6494140000 | 8.1880790000 | 7.9259440000 |
| C | 7.0537120000 | 7.9256860000 | 6.7045640000 |
| C | 7.2657730000 | 6.6706780000 | 6.0731000000 |
| C | 8.1392540000 | 5.7375180000 | 6.5964270000 |
| C | 8.7841030000 | 6.0738410000 | 7.8280660000 |
| C | 8.4922030000 | 7.2800950000 | 8.4343420000 |
| C | 6.2918640000 | 9.0058040000 | 6.1836290000 |
| C | 5.6103520000 | 8.9898960000 | 4.9370990000 |
| C | 4.9127310000 | 10.0849480000 | 4.4715210000 |
| C | 4.9394850000 | 11.2526790000 | 5.2982970000 |
| C | 5.6221500000 | 11.2307290000 | 6.4978360000 |
| C | 8.5068220000 | 4.4409730000 | 5.8669820000 |
| C | 7.5144930000 | 4.0932160000 | 4.7461460000 |
| C | 8.5514670000 | 3.2523950000 | 6.8490130000 |
| C | 9.9101970000 | 4.6421180000 | 5.2478680000 |
| C | 4.0723930000 | 10.0609410000 | 3.1891560000 |
| C | 4.3308170000 | 11.3253540000 | 2.3430320000 |
| C | 2.5818440000 | 10.0323790000 | 3.5986250000 |
| C | 4.3643940000 | 8.8307740000 | 2.3168770000 |
| C | 6.9774560000 | 11.8139740000 | 9.1369720000 |
| C | 8.0582000000 | 12.6620990000 | 8.7678850000 |
| C | 8.0589770000 | 14.0145140000 | 9.1353090000 |
| C | 6.9920570000 | 14.5544460000 | 9.8571570000 |
| C | 5.9218240000 | 13.7371230000 | 10.2023100000 |
| C | 5.8947840000 | 12.3717910000 | 9.8545960000 |
| C | 9.2216770000 | 12.1129700000 | 7.9400990000 |
| C | 9.0903910000 | 12.5023710000 | 6.4583780000 |
| C | 10.6125410000 | 12.5065600000 | 8.4571110000 |
| C | 4.6787420000 | 11.5946010000 | 10.3757260000 |
| C | 4.8686820000 | 11.2913420000 | 11.8744090000 |
| C | 4.2911730000 | 10.3291720000 | 9.6127690000 |
| C | 7.3739000000 | 8.6774990000 | 11.3715730000 |
| C | 8.9225350000 | 10.4640620000 | 11.1449590000 |
| C | 9.2705770000 | 9.7129720000 | 12.4204670000 |
| H | 8.3971220000 | 11.4098670000 | 11.3472110000 |
| H | 9.7727700000 | 10.6687070000 | 10.4845670000 |
| C | 7.9401200000 | 9.0243270000 | 12.7504280000 |
| H | 7.6038840000 | 7.6461100000 | 11.0624860000 |
| H | 6.2883280000 | 8.8252270000 | 11.3101710000 |
| H | 9.6177550000 | 10.3894130000 | 13.2152080000 |
| H | 10.0601360000 | 8.9680850000 | 12.2327130000 |
| H | 8.0492620000 | 8.1316350000 | 13.3827380000 |
| H | 7.2719390000 | 9.7274830000 | 13.2725770000 |
| H | 7.4758080000 | 4.8683010000 | 3.9656520000 |
| H | 6.4954300000 | 3.9558640000 | 5.1411430000 |
| H | 7.8200960000 | 3.1546560000 | 4.2568700000 |

```
H       8.8287550000     2.3301870000     6.3127600000
H       7.5715330000     3.0912480000     7.3264650000
H       9.2912810000     3.3984270000     7.6491540000
H       9.8955530000     5.4570230000     4.5074150000
H      10.2504520000     3.7224440000     4.7431470000
H      10.6488660000     4.9055250000     6.0203050000
H       3.7485030000    11.2773540000     1.4096440000
H       5.3955430000    11.4187100000     2.0753710000
H       4.0302780000    12.2438490000     2.8680630000
H       1.9371930000    10.0309300000     2.7038320000
H       2.3208580000    10.9108220000     4.2086790000
H       2.3570220000     9.1344540000     4.1950630000
H       3.7683860000     8.8802220000     1.3928470000
H       4.0951680000     7.8912910000     2.8284450000
H       5.4278060000     8.7770390000     2.0340390000
H       8.0967420000    12.2469370000     6.0670170000
H       9.8394070000    11.9745580000     5.8462870000
H       9.2463980000    13.5866620000     6.3259500000
H      10.8039610000    13.5827720000     8.3152460000
H      10.7403140000    12.2847510000     9.5285450000
H       5.7297330000    10.6269740000    12.0220520000
H       3.9836130000    10.7874820000    12.3011390000
H       4.0636670000    10.5349420000     8.5598760000
H       5.1040700000     9.5869080000     9.6464990000
H       3.8208540000    12.2855170000    10.2859240000
H       3.4040120000     9.8624370000    10.0726970000
H       5.0652230000    12.2057660000    12.4544020000
H       8.8957610000    14.6602490000     8.8555500000
H       6.9977250000    15.6082280000    10.1475900000
H       5.0792470000    14.1604930000    10.7594310000
H      11.3903360000    11.9606870000     7.9001750000
H       9.1477850000    11.0104510000     7.9841200000
H       5.6564850000     8.0721150000     4.3529540000
H       4.4229180000    12.1675790000     5.0122960000
H       5.6497340000    12.1141730000     7.1340710000
H       8.9655620000     7.5639530000     9.3765190000
H       6.7535940000     6.4774810000     5.1322970000
H       9.5003540000     5.4003690000     8.2974590000
*

%plots format cube
dim1 100 dim2 100 dim3 100
SpinDens("18.cube");
end
```

**Input File for Energy and Mulliken Spin Density Calculation of (dtbpy)Ni(Dipp*) (21)**

```
#21
! UKS B3LYP RIJCOSX SlowConv TightSCF 6-31g* Normalprint UCO OPT FREQ

%basis NewGTO 28 "m6-31G*" end
NewGTO 7 "6-31g*" end
NewGTO 6 "6-31g*" end
NewGTO 1 "6-31g*" end
NewAuxGTO 28 "m6-31G*" end
NewAuxGTO 7 "6-31g*" end
NewAuxGTO 6 "6-31g*" end
NewAuxGTO 1 "6-31g*" end
end


%scf
MaxIter 1500
TolE 1E-7
TolErr 1E-6
end

* xyz 0 2
Coordination from X-ray structure*

%plots format cube
dim1 100 dim2 100 dim3 100
SpinDens("21.cube");
end
```

**Input File for Energy and Mulliken Spin Density Calculation of (dtbpy)Ni(η-3 Dipp*) (23)**

```
#23
! UKS B3LYP d3 RIJCOSX SlowConv TightSCF 6-31g* Normalprint UCO OPT

%basis NewGTO 28 "m6-31G*" end
NewGTO 7 "6-31g*" end
NewGTO 6 "6-31g*" end
NewGTO 1 "6-31g*" end
NewAuxGTO 28 "m6-31G*" end
NewAuxGTO 7 "6-31g*" end
NewAuxGTO 6 "6-31g*" end
NewAuxGTO 1 "6-31g*" end
end

%scf
MaxIter 1500
TolE 1E-7
TolErr 1E-6
```

end

* xyz 0 2
Coordination from X-ray Structure for [(dtbpy)Ni(η-3 Dipp*)][BAr$^F{}_{24}$] (**22**) (the [BAr$^F{}_{24}$]$^-$ counter ion is omitted)*

%plots format cube
dim1 100 dim2 100 dim3 100
SpinDens("23.cube");
end

**Input File for Energy and Mulliken Spin Density Calculation of [(dtbpy)Ni($CH_2$TMS)$_2$]$^-$ (26)**

#26
! UKS B3LYP D3 RIJCOSX SlowConv TightSCF def2-SVP def2/j Normalprint UCO OPT FREQ

%basis NewGTO 28 "def2-TZVPP" end
NewGTO 7 "def2-TZVPP" end
NewGTO 14 "def2-TZVPP" end
NewAuxGTO 28 "def2/j" end
NewAuxGTO 7 "def2/j" end
NewAuxGTO 14 "def2/j" end
end

%scf
MaxIter 1500
TolE 1E-7
TolErr 1E-6
end

* xyz -1 2
Coordination from X-ray Structure (the [K(crown)]$^+$ counter ion is omitted)*

%plots format cube
dim1 100 dim2 100 dim3 100
SpinDens("26.cube");
end

**Input File for Energy and Mulliken Spin Density Calculation of [(phen)NiBr$_2$]$^-$ (28)**

%Chk=28.chk
#n B3LYP empiricaldispersion=gd3 def2TZVPP Opt

28

-1 2
Ni 0.0000000000 0.0000000000 0.0000000000
Br -2.1623623317 -0.0075113373 -0.7383648670

| | | | |
|---|---|---|---|
| Br | 0.3763005876 | -2.2924302311 | 0.0187803515 |
| N | -0.0000000000 | 2.1541541062 | -0.0000000000 |
| N | 1.7870104908 | 0.4197058659 | 0.8597414871 |
| C | -0.9386313338 | 2.9763003202 | -0.4451778890 |
| C | -0.8472319782 | 4.3739908142 | -0.3133389716 |
| C | 0.2645418301 | 4.9292886117 | 0.3219316011 |
| C | 1.0997289150 | 2.6786961437 | 0.6140848762 |
| C | 1.2756270576 | 4.0812772654 | 0.8066140717 |
| C | 2.4649626598 | 4.5223602615 | 1.4808199827 |
| C | 3.4061801068 | 3.6267500453 | 1.9111130637 |
| C | 3.8918374779 | -0.1139925779 | 1.9016173863 |
| C | 2.7045899983 | -0.4781958088 | 1.2748835827 |
| C | 2.0656922413 | 1.7493887969 | 1.0723487163 |
| C | 3.2482761559 | 2.2065538143 | 1.7221227583 |
| C | 4.1807583124 | 1.2426043300 | 2.1417371685 |
| H | -1.7957489645 | 2.4709276131 | -0.9019010819 |
| H | -1.6475962282 | 5.0059176068 | -0.7049851834 |
| H | 0.3551799731 | 6.0115722473 | 0.4564549083 |
| H | 2.6033750988 | 5.5945539907 | 1.6499503700 |
| H | 4.3086105549 | 3.9795116475 | 2.4202684251 |
| H | 4.5906865758 | -0.8966377307 | 2.2075943265 |
| H | 2.4259201714 | -1.5149198217 | 1.0661301557 |
| H | 5.1012108764 | 1.5505445296 | 2.6449729655 |

**Input File for Energy and Mulliken Spin Density Calculation of [(phen)Ni($CH_2$TMS)$_2$]$^-$ (30)**

```
#30
! UKS B3LYP D3 RIJCOSX SlowConv TightSCF def2-SVP def2/j Normalprint UCO OPT FREQ

%basis NewGTO 28 "def2-TZVPP" end
NewGTO 7 "def2-TZVPP" end
NewGTO 14 "def2-TZVPP" end
NewAuxGTO 28 "def2/j" end
NewAuxGTO 7 "def2/j" end
NewAuxGTO 14 "def2/j" end
end

%scf
MaxIter 1500
TolE 1E-7
TolErr 1E-6
end

* xyz -1 2
Ni    10.03351    16.93856    9.66660
Si    10.23096    13.94433    8.75952
Si    12.66740    18.60553    9.37978
```

| | | | |
|---|---|---|---|
| N | 9.47142 | 18.06025 | 11.16719 |
| N | 8.12174 | 17.05006 | 9.19841 |
| C | 8.16341 | 18.42176 | 11.11548 |
| C | 7.50183 | 19.27684 | 12.03427 |
| C | 8.34198 | 20.03723 | 13.01981 |
| C | 9.59711 | 19.25732 | 13.28218 |
| C | 10.10052 | 18.42571 | 12.34759 |
| H | 10.96181 | 18.05890 | 12.51204 |
| C | 7.39469 | 17.84576 | 10.06473 |
| C | 6.01201 | 18.07790 | 9.93712 |
| C | 5.35894 | 17.48508 | 8.83008 |
| H | 4.42887 | 17.61924 | 8.69283 |
| C | 6.07494 | 16.72572 | 7.96961 |
| H | 5.64712 | 16.32300 | 7.22291 |
| C | 7.44571 | 16.53054 | 8.17900 |
| H | 7.92131 | 15.99596 | 7.55443 |
| C | 6.11065 | 19.45230 | 11.91608 |
| H | 5.66046 | 19.97961 | 12.56579 |
| C | 5.37424 | 18.89021 | 10.89540 |
| H | 4.43870 | 19.04978 | 10.83826 |
| C | 10.48318 | 15.68127 | 8.23249 |
| H | 9.91823 | 15.86948 | 7.44210 |
| H | 11.42940 | 15.80952 | 7.96920 |
| C | 8.43212 | 13.38681 | 8.73965 |
| H | 8.05035 | 13.54177 | 7.85119 |
| H | 7.92429 | 13.89523 | 9.40690 |
| H | 8.38338 | 12.43067 | 8.95372 |
| C | 10.80461 | 13.64429 | 10.51152 |
| H | 10.73680 | 12.68931 | 10.71836 |
| H | 10.24066 | 14.15376 | 11.13099 |
| H | 11.73582 | 13.93430 | 10.60432 |
| C | 11.15496 | 12.72858 | 7.64611 |
| H | 10.83614 | 12.82343 | 6.72408 |
| H | 10.99068 | 11.81308 | 7.95547 |
| H | 12.11518 | 12.91822 | 7.68124 |
| C | 11.93388 | 17.06045 | 10.07747 |
| H | 12.06019 | 17.05073 | 11.05934 |
| H | 12.40358 | 16.27431 | 9.70068 |
| C | 14.54447 | 18.58206 | 9.42996 |
| H | 14.87497 | 17.78394 | 8.96862 |
| H | 14.84662 | 18.56858 | 10.36312 |
| H | 14.89380 | 19.38407 | 8.98694 |
| C | 12.21450 | 18.88730 | 7.58498 |
| H | 12.61151 | 19.72776 | 7.27581 |
| H | 11.23923 | 18.93497 | 7.49804 |
| H | 12.55454 | 18.14561 | 7.04203 |
| C | 12.19239 | 20.14251 | 10.33169 |

```
H        12.64835       20.91965        9.94537
H        12.45901       20.04113       11.26951
H        11.22319       20.27289       10.27952
H         8.09707       20.94480       13.44568
H        10.08051       19.35863       14.18832
*

%plots format cube
dim1 100 dim2 100 dim3 100
SpinDens("30.cube");
end
```

**Input File for Energy and Mulliken Spin Density Calculation of (6-Mes-$^{dBn}$pyrox)NiCl (32)**

```
#32
! UKS B3LYP D3 RIJCOSX SlowConv TightSCF def2-SVP def2/j Normalprint UCO OPT FREQ

%basis NewGTO 28 "def2-TZVPP" end
NewGTO 7 "def2-TZVPP" end
NewGTO 8 "def2-TZVPP" end
NewGTO 17 "def2-TZVPP" end
NewAuxGTO 28 "def2/j" end
NewAuxGTO 7 "def2/j" end
NewAuxGTO 8 "def2/j" end
NewAuxGTO 17 "def2/j" end
end

%scf
MaxIter 1500
TolE 1E-7
TolErr 1E-6
end

* xyz 0 2
Coordination from X-ray structure*

%plots format cube
dim1 100 dim2 100 dim3 100
SpinDens("32.cube");
end
```

**Input File for Energy and Mulliken Spin Density Calculation of ($^{tBu}$pyrox)Ni(Dipp) (35)**

```
#35
! UKS B3LYP D3 RIJCOSX SlowConv TightSCF def2-SVP def2/j Normalprint UCO OPT FREQ

%basis NewGTO 28 "def2-TZVPP" end
```

```
NewGTO 7 "def2-TZVPP" end
NewGTO 8 "def2-TZVPP" end
NewAuxGTO 28 "def2/j" end
NewAuxGTO 7 "def2/j" end
NewAuxGTO 8 "def2/j" end
end

%scf
MaxIter 1500
TolE 1E-7
TolErr 1E-6
end

* xyz 0 2
Ni     0.237497   -0.188376    0.097484
O      2.338794   -2.583928   -2.563249
N      1.370130   -1.824968   -0.663187
N      0.258307    0.266676   -1.853391
C     -3.657065   -0.212895    1.541986
C      3.276211    0.762382    2.708970
C      2.291105    3.060921    2.319535
C     -2.690850   -2.340080    2.541152
C     -2.368963   -0.997140    1.857415
C      2.065653    1.547131    2.170119
C      0.745443    1.066481    2.777004
C      0.443775    1.408665    4.106036
C     -0.748828    0.984801    4.697563
C     -1.642034    0.205033    3.960955
C     -1.353829   -0.151633    2.631875
C     -0.149321    0.272501    2.005812
C      2.205937   -5.244455    0.946200
C      1.245611   -3.243307    2.111377
C     -0.032242   -4.309596    0.224374
C      1.372230   -3.959456    0.751623
C      2.549630   -3.651285   -1.590769
C      2.130686   -3.024865   -0.234665
C      1.577416   -1.671761   -1.917827
C      0.599051    0.821963   -4.582261
C      1.211887   -0.294523   -4.007553
C      1.011155   -0.537445   -2.648119
C     -0.337894    1.326652   -2.419955
C     -0.199650    1.639035   -3.772843
H     -4.187863    0.080135    2.467360
H     -3.428556    0.712902    0.983786
H     -4.354580   -0.819078    0.932016
H      3.393245    0.906828    3.799994
H      3.160163   -0.321859    2.524622
```

```
H      4.213892    1.094884    2.222399
H      2.446520    3.355556    3.373893
H      1.427596    3.634126    1.937118
H      3.187759    3.381036    1.755141
H     -3.239731   -2.197306    3.491207
H     -1.768912   -2.902715    2.770558
H     -3.321644   -2.972051    1.887055
H     -1.886427   -1.232741    0.887493
H      1.995698    1.329498    1.086594
H      1.142869    2.016256    4.693269
H     -0.984908    1.269436    5.729239
H     -2.574967   -0.125347    4.432735
H      2.304475   -5.835637    0.016890
H      1.725769   -5.896015    1.698219
H      3.223249   -5.011769    1.313936
H      0.693272   -2.292516    2.027499
H      2.242315   -3.013020    2.530920
H      0.716899   -3.888995    2.835802
H     -0.646896   -3.401684    0.102480
H     -0.002694   -4.838606   -0.747226
H     -0.549935   -4.972802    0.939969
H      1.915062   -4.499551   -1.895430
H      3.604766   -3.954491   -1.644423
H      3.028952   -2.656286    0.295671
H      0.744798    1.054722   -5.641090
H      1.838072   -0.973463   -4.590947
H     -0.938479    1.946736   -1.749168
H     -0.707391    2.517211   -4.180473
*

%plots format cube
dim1 100 dim2 100 dim3 100
SpinDens("35.cube");
end
```

**Spin-Density Plots**

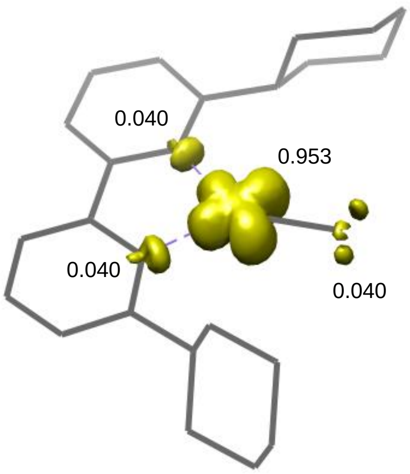


**Figure S73**. Spin density plot for $^{Cy}$bpyNiCl (**12**) obtained from Mulliken population analysis.

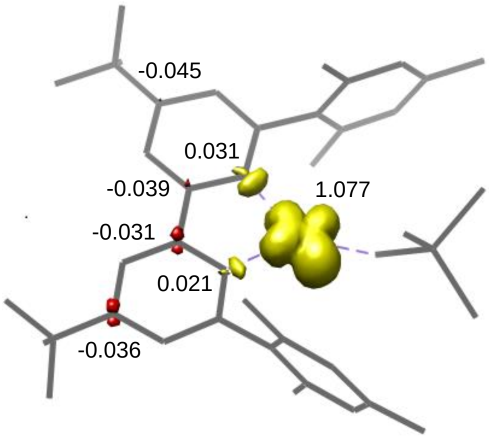


**Figure S74**. Spin density plot for ($^{Mes}$bpy)Ni($CH_2$TMS) (**14**) obtained from Mulliken population analysis.

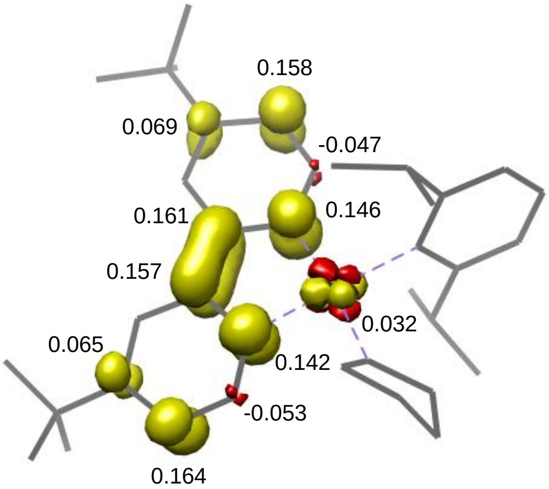


**Figure S75**. Spin density plot for (dtbpy)Ni(Dipp)•THF (**16**) obtained from Mulliken population analysis.

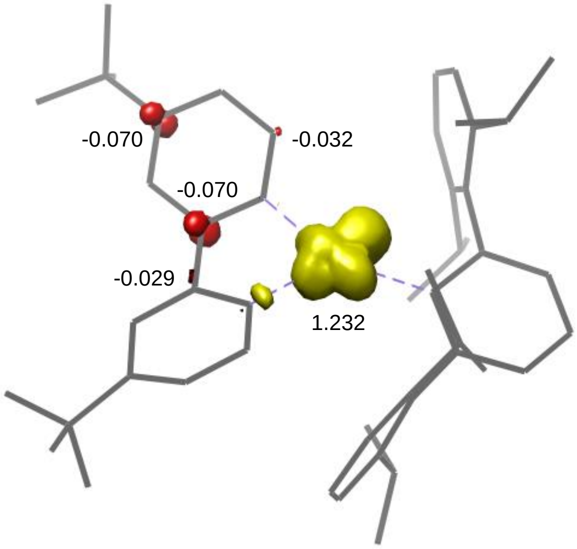


**Figure S76**. Spin density plot for (dtbpy)Ni(Dipp*) (**21**) obtained from Mulliken population analysis.

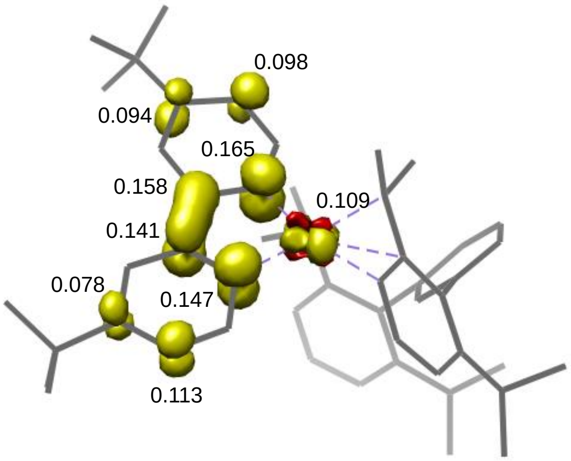


**Figure S77**. Spin density plot for (dtbpy)Ni(η-3 Dipp*) (**23**) obtained from Mulliken population analysis.

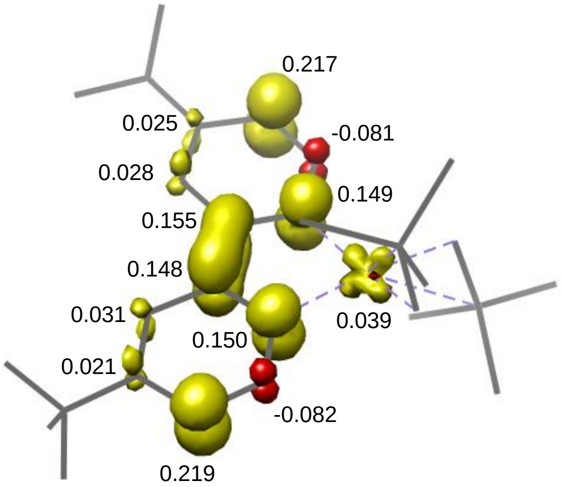


**Figure S78**. Spin density plot for $[(dtbpy)Ni(CH_2TMS)_2]^-$ (**26**) obtained from Mulliken population analysis. Coordination of $[K(crown)]^+$ counter ion is omitted.

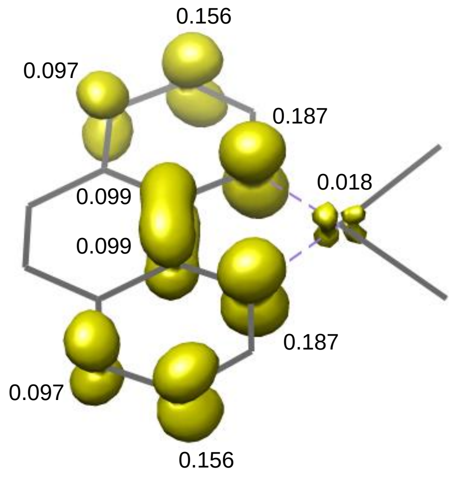


**Figure S79**. Spin density plot for $[(phen)NiBr_2]^-$ (**28**) obtained from Mulliken population analysis. Coordination of $[K(crown)]^+$ counter ion is omitted.

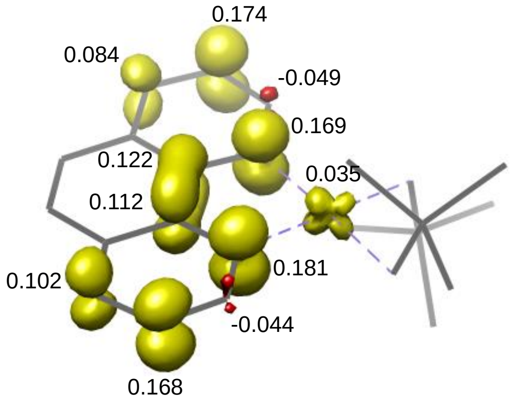


**Figure S80**. Spin density plot for $[(phen)Ni(CH_2TMS)_2]_2^-$ (**30**) obtained from Mulliken population analysis. Coordination of $[K(crown)]^+$ counter ion is omitted.

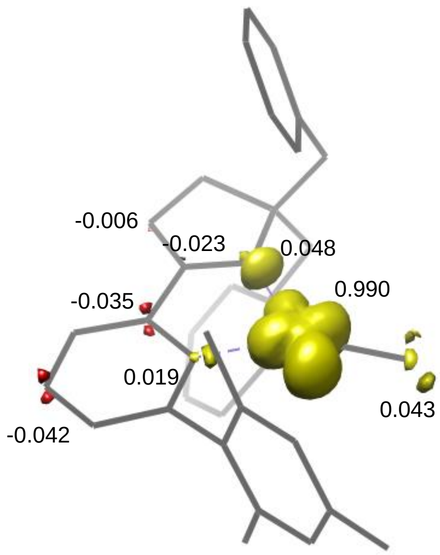


**Figure S81**. Spin density plot for (6-Mes-$^{dBn}$pyrox)NiCl (**32**) obtained from Mulliken population analysis.

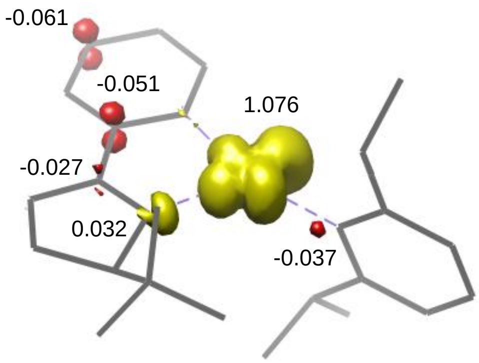


**Figure S82**. Spin density plot for ($^{tBu}$pyrox)Ni(Dipp) (**35**) obtained from Mulliken population analysis.

## 9. References

1. Ward, L.; Pipal, J. R. Anhydrous Nickel (II) Halides and their Tetrakis(ethanol) and 1,2-Dimethoxyethane Complexes. In *Inorganic Syntheses, Volume 13*; Cotton, F. A.; McGraw-Hill, **1972**; pp. 158–163.

2. Lam, Y. H.; Bobbio, C.; Cooper, I.; Gouverneur, V. A Concise Synthesis of Enantioenriched Fluorinated Carbocycles. *Angew. Chem. Int. Ed.* **2007**, *119*, 5198–5202.

3. Wallasch, M.; Weismann, D.; Riehn, C.; Ambrus, S.; Wolmershäuser, G.; Lagutschenkov, A.; Niedner-Schatteburg, G.; Sitzmann, H. Reactive Sigma-Aryliron Complexes or Iron-Promoted

Coupling of Two Phenyl Anions to One Bis(cyclohexadienylidene) Ligand: Synthesis, Structure, Mass Spectrometry, and DFT Calculations. *Organometallics* **2010**, *29*, 806–813.

4. Schiemenz, B.; Power, P. Synthesis and Structure of a Unique Monomeric σ-Bonded Aryllithium Compound Stabilized by a Weak Li-Benzene π interaction. *Angew. Chem. Int. Ed.* **1996**, *35*, 2150–2152.

5. Qi, X.; Chen, C.; Hou, C.; Fu, L.; Chen, P.; Liu, G. Enantioselective Pd(II)-Catalyzed Intramolecular Oxidative 6-endo Aminoacetoxylation of Unactivated Alkenes. *J. Am. Chem. Soc.* **2018**, *140*, 7415–7419.

6. Li, Z. Q.; Fu, Y.; Deng, R.; Tran, V.; Gao, Y.; Liu, P.; Engle, K. Ligand-Controlled Regiodivergence in Nickel-Catalyzed Hydroarylation and Hydroalkenylation of Alkenyl Carboxylic Acids. *Angew. Chem. Int. Ed.* **2020**, *59*, 23306–23312.

7. Frisch, M. J.; Trucks, G. W.; Schlegel, H. B.; Scuseria, G. E.; Robb, M. A.; Cheeseman, J. R.; Scalmani, G.; Barone, V.; Petersson, G. A.; Nakatsuji, H.; Li, X.; Caricato, M.; Marenich, A. V.; Bloino, J.; Janesko, B. G.; Gomperts, R.; Mennucci, B.; Hratchian, H. P.; Ortiz, J. V.; Izmaylov, A. F.; Sonnenberg, J. L.; Williams; Ding, F.; Lipparini, F.; Egidi, F.; Goings, J.; Peng, B.; Petrone, A.; Henderson, T.; Ranasinghe, D.; Zakrzewski, V. G.; Gao, J.; Rega, N.; Zheng, G.; Liang, W.; Hada, M.; Ehara, M.; Toyota, K.; Fukuda, R.; Hasegawa, J.; Ishida, M.; Nakajima, T.; Honda, Y.; Kitao, O.; Nakai, H.; Vreven, T.; Throssell, K.; Montgomery Jr. J. A.; Peralta, J. E.; Ogliaro, F.; Bearpark, M. J.; Heyd, J. J.; Brothers, E. N.; Kudin, K. N.; Staroverov, V. N.; Keith, T. A.; Kobayashi, R.; Normand, J.; Raghavachari, K.; Rendell, A. P.; Burant, J. C.; Iyengar, S. S.; Tomasi, J.; Cossi, M.; Millam, J. M.; Klene, M.; Adamo, C.; Cammi, R.; Ochterski, J. W.; Martin, R. L.; Morokuma, K.; Farkas, O.; Foresman, J. B.; Fox, D. J. *Gaussian 16 Rev. B.01,* Gaussian, Inc.: Wallingford, CT, **2016**.

8. (a) Neese, F. The ORCA program system. *Wiley Interdiscip. Rev.: Comput. Mol. Sci.* **2011**, *2*, 73-78. (b) Neese, F. Software update: the ORCA program system, version 4.0. *Wiley Interdiscip. Rev.: Comput. Mol. Sci.* **2017**, *8*. e1327.

9. Mohadjer Beromi, M.; Brudvig, G. W.; Hazari, N.; Lant, H. M. C.; Mercado, B. Q. Synthesis and Reactivity of Paramagnetic Nickel Polypyridyl Complexes Relevant to C(sp2)–C(sp3)Coupling Reactions. *Angew. Chem. Int. Ed.* **2019**, *131*, 6155–6159.

10. Klein, A.; Kaiser, A.; Sarkar, B.; Wanner, M.; Fiedler, J. The Electrochemical Behaviour of Organonickel Complexes: Mono-, Di- and Trivalent Nickel. *Eur. J. Inorg. Chem.* **2007**, *2007*, 965–976.

11. Irwin, M.; Doyle, L. R.; Krämer, T.; Herchel, R.; McGrady, J. E.; Goicoechea, J. M. A Homologous Series of First-Row Transition-Metal Complexes of 2,2'-Bipyridine and their Ligand Radical Derivatives: Trends in Structure, Magnetism, and Bonding. *Inorg. Chem.* **2012**, *51*, 12301–12312.

12. Humphrey, E. L. B. J.; Kennedy, A. R.; Sproules, S.; Nelson, D. J. Evaluating a Dispersion of Sodium in Sodium Chloride for the Synthesis of Low-Valent Nickel Complexes. *Eur. J. Inorg. Chem.* **2022**, *2022*, No. e202101006.

13. Lin, Q.; Diao, T. Mechanism of Ni-Catalyzed Reductive 1,2-Dicarbofunctionalization of Alkenes. *J. Am. Chem. Soc.* **2019**, *141*, 17937–17948.

14. Somerville, R.; Odena, C.; Obst, M.; Hazari, N.; Hopmann, K.; Martin, R. Ni(I)-Alkyl Complexes Bearing Phenanthroline Ligands: Experimental Evidence for $CO_2$ Insertion at Ni(I) Centers. *J. Am. Chem. Soc.* **2020**, *142*, 10936–10941.

15. Wagner, C.; Herrera, G.; Lin, Q.; Hu, C.; Diao, T. Redox Activity of Pyridine-Oxazoline Ligands in the Stabilization of Low-Valent Organonickel Radical Complexes. *J. Am. Chem. Soc.* **2021**, *143*, 5295–5300.

16. Ju, L.; Lin, Q.; LiBretto, N. J.; Wagner, C. L.; Hu, C. T.; Miller, J. T.; Diao, T. Reactivity of (bi-Oxazoline)organonickel Complexes and Revision of a Catalytic Mechanism. *J. Am. Chem. Soc.* **2021**, *143*, 14458–14463.

17. Kuang, Y. Anthony, D.; Katigbak, J.; Marrucci, F.; Humagain, S.; Diao, T. Ni(I)-Catalyzed Reductive Cyclization of 1,6-Dienes: Mechanism-Controlled *trans* Selectivity. *Chem* **2017**, *3*, 268–280.
18. Dong, Q.; Zhao, Y.; Su, Y.; Su, J.-H.; Wu, B.; Yang, X.-J. Synthesis and Reactivity of Nickel Hydride Complexes of an a-Diimine Ligand. *Inorg. Chem.* **2012**, *51*, 13162–13170.
19. Zarate, C.; Yang, H.; Bezdek, M. J.; Hesk, D.; Chirik, P. J. Ni(I)-X Complexes Bearing a Bulky a-Diimine Ligand: Synthesis, Structure, and Superior Catalytic Performance in the Hydrogen Isotope Exchange in Pharmaceuticals. *J. Am. Chem. Soc.* **2019**, *141*, 5034–5044.
20. Jones, G. D.; Martin, J. L.; McFarland, C.; Allen, O. R.; Hall, R. E.; Haley, A. D.; Brandon, R. J.; Konovalova, T.; Desrochers, P. J.; Pulay, P.; Vicic, D. A. Ligand Redox Effects in the Synthesis, Electronic Structure, and Reactivity of an Alkyl-Alkyl Cross-Coupling Catalyst. *J. Am. Chem. Soc.* **2006**, *128*, 13175–13183.
21. Ciszewski, J. T.; Mikhaylov, D. Y.; Holin, K. V.;; Kadirov, M. K.; Budnikova, Y. H.; Sinyashin, O.; Vicic, D. A. Redox Trends in Terpyridine Nickel Complexes. *Inorg. Chem.* **2011**, *50*, 8630–8635.
22. Anderson, T. J.; Jones, G. D.; Vicic, D. A. Evidence for a $Ni^{I}$ Active Species in the Catalytic Cross-Coupling of Alkyl Electrophiles. *J. Am. Chem. Soc.* **2004**, *126*, 8100–8101.
23. Bismuto, A.; Müller, P.; Finkelstein, P.; Trapp, N.; Jeschke, G.; Morandi, B. One to Find Them All: A General Route to Ni(I)-Phenolate Species. *J. Am. Chem. Soc.* **2021**, *143*, 10642–10648.
24. Schley, N. D.; Fu, G. C. Nickel-Catalyzed Negishi Arylations of Propargylic Bromides: A Mechanistic Investigation. *J. Am. Chem. Soc.* **2014**, *136*, 16588–16593.
25. Hintermann, L.; Xiao, L.; Labonne, A. A General and Selective Copper-Catalyzed Cross-Coupling of Tertiary Grignard Reagents with Azacyclic Electrophiles. *Angew. Chem. Int. Ed.* **2008**, *47*, 8246–8250.
26. (a) Katz, H. E. Chelate and macrocycle effects in the 2,2'-bipyridine N,N'-dioxide complexation of alkyltin trichlorides. *J. Org. Chem.* **1985**, *50*, 2086–2091. (b) Fujita, K. I.; Wada, T.; Shiraishi, T. Reversible Interconversion between 2,5-Dimethylpyrazine and 2,5-Dimethylpiperazine by Iridium-Catalyzed Hydrogenation/Dehydrogenation for Efficient Hydrogen Storage. *Angew. Chem. Int. Ed.* **2017**, *56*, 10886–10889.
27. Kohler, L.; Hayes, D.; Hong, J.; Carter, T.; Shelby, M.; Fransted, K.; Chen, L.; Mulfort, K. Synthesis, structure, ultrafast kinetics, and light-induced dynamics of CuHETPHEN chromophores. *Dalton Trans.* **2016**, *45*, 9871–9883.
28. Ting, S.; Garakyaraghi, S.; Taliaferro, C.; Shields, B.; Scholes, G.; Castellano, F.; Doyle, A. $^{3}$d-d Excited States of Ni(II) Complexes Relevant to Photoredox Catalysis: Spectroscopic Identification and Mechanistic Implications. *J. Am. Chem. Soc.* **2020**, *142*, 5800–5810.
29. Lin, Q.; Spielvogel, E.; Diao, T. Carbon-centered radical capture at nickel(II) complexes: Spectroscopic evidence, rates, and selectivity. *Chem* **2023**, *9*, 1295–1308.
30. (a) Guizzetti, S.; Benaglia, M.; Rossi, S. *Org. Lett.* **2009**, *11*, 2928–2931. (b) Holder, J. Shockley, S.; Wiesenfeldt, M.; Shimizu, H.; Stoltz, B.; Cochran, B. Preparation of (S)-*tert*-ButylPyOx and Palladium-Catalyzed Asymmetric Conjugate Addition of Arylboronic Acids. *Org. Syn.* **2015**, *92*, 247–266.
31. (a) Sheldrick, G. M. SHELXTL, An Integrated System for Solving, Refining, and Displaying Crystal Structures from Diffraction Data; University of Göttingen, Göttingen, Federal Republic of Germany,1981.
(b) Sheldrick, G. M. SHELXT-Integrated Space-Group and Crystal-Structure Determination. *Acta Cryst.* **2015**, *A71*, 3–8.
32. Bruker (**2020**). *APEX3*. Bruker AXS LLC, Madison, WI, USA.
33. Bruker (**2020**). *SAINT*. Bruker AXS LLC, Madison, WI, USA.
34. Sheldrick, G. M. Crystal structure refinement with SHELXL. *Acta. Cryst.* **2015**, *C71*, 3–8.

35. Hübschle, C.B.; Sheldrick, G. M.; Dittrich, B. ShelXle: a Qt graphical user interface for SHELXL. *J. Appl. Cryst.* **2011**, *44*, 1281–1284.

36. Dolomanov, O. V.; Bourhis, L. J.; Gildea, R. J.; Howard, J. A. K.; Puschmann, H. OLEX2: A complete structure solution, refinement and analysis program. *J. Appl. Cryst.* **2009**, *42*, 339–341.

37. (a) Becke, A. D. Density-Functional Thermochemistry. III. The Role of Exact Exchange. *J. Chem. Phys.* **1993**, *98*, 5648–5652. (b) Lee, C.; Yang, W.; Parr, R. G. Development of the Colle-Salvetti Correlation-Energy Formula into a Functional of the Electron Density. *Phys. Rev. B* **1988**, *37*, 785–789. (c) Grimme, S. Semiempirical GGA-Type Density Functional Constructed with a Long-Range Dispersion Correction. *J. Comput. Chem.* **2006**, *27*, 1787–1799.

38. (a) Grimme, S.; Ehrlich, S.; Goerigk, L. Effect of the damping function in dispersion corrected density functional theory. *J. Comput. Chem.* **2011**, *32*, 1456–1465. (b) Grimme, S.; Antony, J.; Ehrlich, S.; Krieg, H. A consistent and accurate ab initio parametrization of density functional dispersion correction (DFT-D) for the 94 elements H-Pu. *J. Chem. Phys.* **2010**, *132*. (c) Weigend, F.; Ahlrichs, R. Balanced basis sets of split valence, triple zeta valence and quadruple zeta valence quality for H to Rn: Design and assessment of accuracy. *Phys. Chem. Chem. Phys.* **2005**, *7*, 3297–3305. (d) Weigend, F. Accurate Coulomb-fitting basis sets for H to Rn. *Phys. Chem. Chem. Phys.* **2006**, *8*, 1057.

39. (a) Becke, A. D. Density-functional exchange-energy approximation with correct asymptotic behavior. *Phys. Rev. A* **1988**, *38*, 3098–3100. (b) Perdew, J. P. Density-functional approximation for the correlation energy of the inhomogeneous electron gas. *Phys. Rev. B* **1986**, *33*, 8822–8824.

40. Chai, J.-D.; Head-Gordon, M. Long-Range Corrected Hybrid Density Functionals with Damped Atom–Atom Dispersion Corrections. *Phys. Chem. Chem. Phys.* **2008**, *10*, 6615–6620.

41. (a) Tao, J.; Perdew, J. P.; Staroverov, V. N.; Scuseria, G. E. Climbing the Density Functional Ladder: Nonempirical Meta–Generalized Gradient Approximation Designed for Molecules and Solids. *Phys. Rev. Lett.* **2003**, *91*, 146401. (b) Staroverov, V. N.; Scuseria, G. E.; Tao, J.; Perdew, J. P. Comparative assessment of a new nonempirical density functional: Molecules and hydrogen-bonded complexes. *J. Chem. Phys.* **2003**, *119*, 12129–12137.

42. Mitin, A. V.; Baker, J.; Pulay, P. An improved 6-31G* basis set for first-row transition metals. *J. Chem. Phys.* **2003**, 118, 7775–7782.
